# Presenting a new fluorescent probe, methyl(10-phenylphenanthren-9-yl)sulfane sensitive to the polarity and rigidity of the microenvironment: applications toward microheterogeneous systems†

**DOI:** 10.1039/d4ra05565a

**Published:** 2024-08-16

**Authors:** Shalini Dyagala, Nilanjana Mukherjee, Sayantan Halder, Heena Charaya, Mohammed Muzaffar-Ur-Rehman, Sankaranarayanan Murugesan, Shamik Chakraborty, Tanmay Chatterjee, Subit Kumar Saha

**Affiliations:** a Department of Chemistry, Birla Institute of Technology & Science (BITS) Pilani, Hyderabad Campus Hyderabad Telangana 500078 India sksaha@hyderabad.bits-pilani.ac.in sksaha@pilani.bits-pilani.ac.in subitksaha@gmail.com tanmay@hyderabad.bits-pilani.ac.in +91-40-66303643 +91-40-66303-680; b Department of Chemistry, Birla Institute of Technology & Science (BITS) Pilani, Pilani Campus Rajasthan 333031 India shamik@pilani.bits-pilani.ac.in +91 1596 515716; c Department of Pharmacy, Birla Institute of Technology & Science (BITS) Pilani, Pilani Campus Rajasthan 333031 India

## Abstract

A molecule, methyl(10-phenylphenanthren-9-yl)sulfane (MPPS), with a straightforward structure, has been synthesized, characterized, and explored as a new fluorescent probe for microheterogeneous systems. The photophysical properties of MPPS have been studied through experimental and theoretical calculations using the range-separated hybrid functional CAM-B3LYP in conjunction with a 6-311++g(d,p) basis set. Theoretical calculations show that the freely rotating phenyl ring forms a 94° dihedral angle with the phenanthrene ring in the ground state. Experimentally found two absorption bands correspond to the n → π^*^ and π → π^*^ transitions supported by the frontier molecular orbital calculations. Two excited singlet states, E-1 and E-2 (the former being more stable than the latter in the gas phase), exist with dihedral angles between the phenyl and phenanthrene rings as 142° and 133°, respectively, in the gas phase. Two emitting states in a condensed medium of varying polarities are supported by the steady-state fluorescence and fluorescence intensity decay data. Emission energies, fluorescence intensities, and excited singlet state lifetimes change with the polarity of the solvents. To support that the free rotation in the molecule is responsible for these changes, the fluorescence properties of another molecule, methyl(10-(*o*-tolyl)phenanthren-9-yl)sulfane (MTPS), with restricted rotation of the substituted benzene, *i.e.*, *o*-tolyl ring have been studied. The fast-intensity decay component of MPPS is ascribed to the conformer in the E-1 state. The molecule has proved to be an excellent polarity probe explored to determine the critical micelle concentrations (cmc) values of different surfactants, which agree well with the literature reports. Different regions of binding isotherm (specific, non-cooperative, cooperative, and massive binding) of a gemini surfactant, 12-6-12,2Br^−^ with bovine serum albumin (BSA) have been successfully demonstrated by the steady-state and time-resolved fluorescence and fluorescence anisotropic properties of MPPS. Docking results show that MPPS resides in the hydrophobic pocket of BSA. The fluorescence quenching of BSA by MPPS reveals the location of Trp residues of BSA. Thus, a polarity and molecular rigidity-sensitive fluorescent molecule, MPPS has been presented here that can potentially be used to monitor the changes in the microenvironment of biomolecules in different processes.

## Abstract 2

Methyl(10-phenylphenanthren-9-yl)sulfane (MPPS) is synthesized, characterized and explored as a new fluorescent molecule to probe micelle formation, microenvironment of BSA's tryptophan residues, and BSA-gemini surfactant (12-6-12,2Br^−^) interactions.

## Graphical abstract

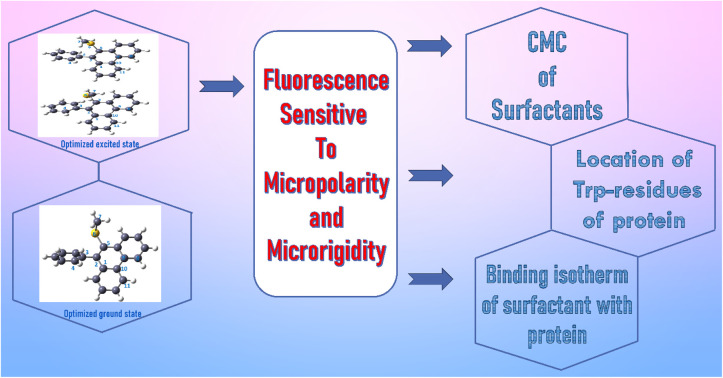

## 1. Introduction

Fluorescent probes have been extensively used to characterize different microheterogeneous systems such as micelles,^1–6^ reverse micelles,^7–11^ vesicles,^12^ proteins,^4,13,14^ and their structure and dynamics,^6,15^ and protein's interactions with several bioactive molecules.^4,16–18^ Molecules with intramolecular charge transfer (ICT) fluorescence properties have been utilized to demonstrate proteins' unfolding in the presence of denaturing substances.^19–22^ The fluorescence probe technique has garnered immense popularity in studying a surfactant's micellization/adsorption properties,^23,24^ polymer–surfactant interactions,^25,26^ protein–surfactant interactions,^19,27^*etc.* Saha and co-workers^13^ have used *trans*-2-[4-(dimethylamino)styryl]benzthiazole (DMASBT), showing TICT properties as a fluorescent biomarker to demonstrate the unfolding of a protein, bovine serum albumin (BSA) by gemini surfactants, 12-4-12,2Br^−^ and 12-8-12,2Br^−^ and their binding isotherms with BSA. The choice of a suitable probe to study a particular system is important. Depending on the polarity of a probe molecule, the binding/solubilizing site of the probe is changed, and accordingly, the fluorescence properties are altered. For example, the polarity of the solubilizing medium affects the fluorescence quantum yields of molecules like pyrene and pyrene-3-carboxaldehyde,^28^ and therefore, the fluorescence behavior in micellar and nonmicellar solutions alters. Such fluorescence fluctuations concerning surfactant concentration were used to determine the critical micelle concentration (cmc) of different surfactants.^29^

Protein-surfactant interactions form a substantial basis for pharmaceutical, food processing, and biochemistry applications.^30,31^ Globular proteins, denatured by different surfactants, undergo changes in protein conformation, which often interrupt biological functions, leading to diseases like neurological disorders.^32^ BSA (molecular weight ∼66.5 kDa) possesses 583 amino acid residues within a single polypeptide chain. It contains nine loops supported by 17 disulfide bonds, which fringes in three domains, I, II, and III. Each domain again has two sub-domains, namely, A and B. Out of the two tryptophan (Trp) residues, Trp-134 exists in a hydrophilic subdomain, and Trp-213 resides in the hydrophobic pocket.^33^ There is negligible fluorescence from the tyrosine (Tyr) residues in BSA, and BSA's fluorescence is majorly due to the Trp residues having an excitation at 295 nm.^34^ BSA interacts with various amphiphilic biological molecules and plays a pivotal role in physiological function.^35^ Substantial research has been carried out on protein–surfactant interaction.^36–39^ Although the binding isotherm related to the binding between conventional surfactants and protein has been broadly studied, there is ample scope for exploring the binding isotherm of ionic gemini surfactants because they possess unusual structures corresponding to their conventional counterparts. Gemini surfactants comprise two hydrophobic chains and two polar headgroups. The headgroups remain covalently linked by a spacer group at heads.^40,41^ As they are more surface-active, they bear a greater affinity towards protein than their conventional single-chain counterparts.^41^ Several extrinsic fluorescent probes have often been used to investigate protein–surfactant interactions.^4,42^ The polarity probes demonstrated the binding interactions between protein and surfactants, emphasizing the microenvironment changes of the tryptophan residues of the protein involved, frequently supported by fascinating theoretical approaches^4^ and biological activity studies.^43^

Geometry optimization and electronic structure calculations for a synthesized fluorescent probe molecule at both ground and excited states, considering the effect of solvent polarities, have implications for supporting the experimental observations. Computational approaches using density functional theory (DFT) have often been proposed and utilized to shed light on the ground and excited state calculations of large systems.^44–48^

Phenanthrene is one of the essential classes of polycyclic aromatic hydrocarbons found in numerous biologically active molecules, including natural products.^49,50^ Notably, phenanthrene derivatives indeed possess intriguing electronic and optical properties, making them valuable components in various materials and devices.^51–55^ Moreover, phenanthrenes are also utilized as fluorophores or fluorescent probes for various systems.^56–58^

**Fig. 1:**
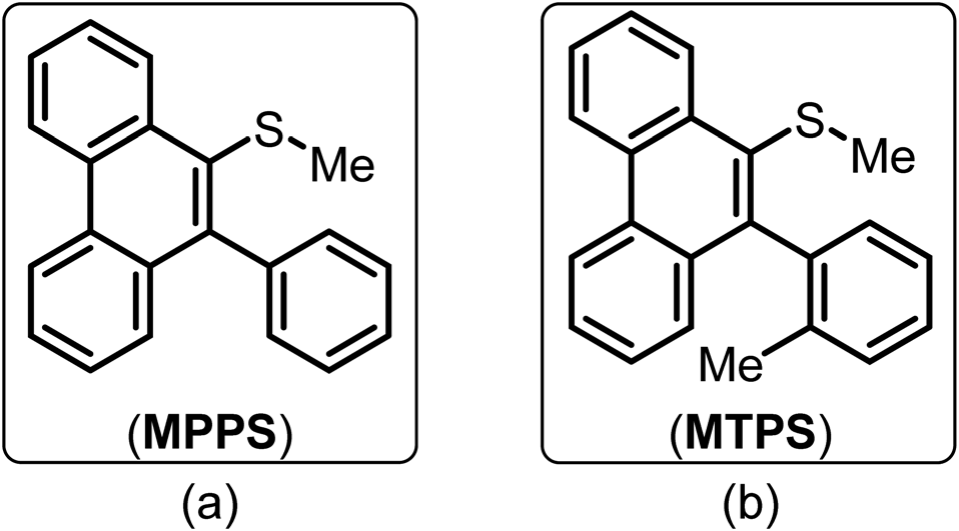
Molecular structures of (a) methyl(10-phenylphenanthren-9-yl)sulfane (MPPS) and (b) methyl(10-(*o*-tolyl)phenanthren-9-yl)sulfane (MTPS).

Being motivated by the unique characteristics of phenanthrenes and their wide range of applications, in this work, a couple of phenanthrene-based molecules, *i.e.*, methyl(10-phenylphenanthren-9-yl)sulfane (MPPS) and methyl(10-(*o*-tolyl)phenanthren-9-yl)sulfane (MTPS) have been synthesized and applied as new fluorescent probes (Fig. 1). Although the synthesis of MPPS was reported earlier,^59^ the synthesis of MTPS is reported here for the first time. Both the molecules are simple phenanthrene derivatives bearing a –SMe and an aryl functional group in the 9, and 10 positions, respectively. The only difference between these two molecules is an extra methyl group in MTPS in the phenyl ring, which is purposefully designed to increase the rigidity in the molecule due to the high energy barrier of the C–C single bond rotation in between the phenanthrene and *o*-tolyl (substituted phenyl) ring.

The molecule MPPS was initially utilized to explore its solvatochromic properties. The probe's absorption and fluorescence properties altered based on the polarity of the medium, which were further explained by some theoretical calculations using Density Functional Theory (DFT) and Time-Dependent Density Functional Theory (TD-DFT) methods. The ground and excited state geometry optimization and electronic structure calculations were performed using the range-separated hybrid functional CAM-B3LYP in conjunction with the 6-311++g(d,p) basis set. Along with the calculations in the gas phase, the effect of solvents of different polarities, carbon tetrachloride (CCl_4_), methanol (MeOH), acetonitrile (ACN), and water (H_2_O) have been investigated using the polarisable continuum model (PCM) based on the integral equation formalism (IEFPCM) variant. The frontier molecular orbital (FMO) calculations depict the electronic configurations in the gas phase. The two conformers of MPPS are identified in the excited states, E-1 and E-2, with the former being more stable than the latter in the gas phase.

The molecule MPPS has been utilized through experimental, theoretical, and docking studies to present it as an efficient polarity and rigidity probe to demonstrate the aggregation of surfactants of various types (cationic, anionic, and non-ionic) and estimation of their critical micellar concentration (cmc), the location of Trp residues of BSA and their accessibility to a fluorescence quencher, and the binding isotherm of a gemini surfactant, 12-6-12,2Br^−^ with BSA. The steady-state fluorescence and fluorescence anisotropy and the time-resolved fluorescence and fluorescence anisotropy data of MPPS have been successfully utilized to further support the binding isotherm at different regions, such as specific, non-cooperative, cooperative, and massive binding. The theoretical results on the changes in the dihedral angles of MPPS in two excited states, E-1 and E-2 with the polarity of solvents have been utilized to explain variation in fluorescence intensity, decay components, and their weightages during denaturation of BSA by 12-6-12,2Br^−^. To support the fact that free rotation of the phenyl ring of MPPS leading to the coplanarity between the phenyl and phenanthrene rings is responsible for the enhancement of fluorescence, another molecule, methyl(10-(*o*-tolyl)phenanthren-9-yl)sulfane (MTPS) (Fig. 1) with restricted free rotation has been synthesized. Its theoretical calculations are carried out, and lifetimes in native BSA, and BSA in the presence of different concentrations of surfactants have been measured.

It has been noted that the fluorescence properties of MPPS are sensitive not only to the polarity but also to the rigidity of the microenvironment. Thus, the molecule can be successfully utilized as a fluorescent probe for various microheterogeneous systems. Despite notable advancements in this area, the development of a single fluorescent probe to sense both the polarity and the rigidity of various microenvironments is not very common in the literature.

## 2. Experimental section

### 2.1. Materials & methods

#### 2.1.1. Materials used for solvatochromic, and microheterogeneous system study

Bovine serum albumin (BSA) with a purity of 98%, dodecyltrimethylammonium bromide (DTAB) having >97% purity, sodium dodecyl sulfate (SDS) with >99% purity, Triton-X 100 and quinine sulfate [purity = 99%] were all procured from Sigma Aldrich and used as received. The different spectroscopic grade solvents, such as hexane [purity > 99%], cyclohexane [purity = 99.5%], carbon tetrachloride [purity = 99.9%] were obtained from SRL, India and dioxane [purity = 99.8%], ethyl acetate [purity = 99.8%], methanol [purity = 99.8%], and ethanol [purity = 99.9%] were obtained from Spectrochem Pvt. Ltd. 2-(4-(2-hydroxyethyl)-1-piperazinyl)-ethanesulfonic acid (HEPES) buffer was obtained from SRL, India. Sulfuric acid (H_2_SO_4_) [purity = 99.99%] and sodium hydroxide (NaOH), received from SRL, India, were used as obtained. The gemini surfactant, 12-6-12,2Br^−^, was prepared using the reported method, supported by suitable FT-IR and NMR data.^60^

#### 2.1.2 Procedures for the synthesis of probes

Materials used for the synthesis and characterization of the probes are given in the ESI† under general experimental information. The designed 9-methylsulfonyl phenanthrene derivatives *i.e.*, MPPS and MTPS were synthesized by following one^59^ of our recently developed synthetic methods to access chalcogenyl phenanthrenes^61–63^ using DMSO as the source of methylsulfenyl group (SMe). The synthetic route to MPPS and MTPS is presented in Scheme S1 in the ESI†.

The structures of MPPS^59^ and MTPS were confirmed by ^1^H and ^13^C Nuclear Magnetic Resonance (NMR) (Fig. S1†) spectroscopy and High-Resolution Mass Spectrometry (HRMS).^37,59,64^ The detailed experimental procedures for all three steps to access MPPS and MTPS are provided in the ESI.†

#### 2.1.3 Methods for theoretical calculations

All the electronic structure calculations were performed using the Gaussian 16 program package^65^ at the Density Functional Theory (DFT) and Time-Dependent Density Functional Theory (TD-DFT) levels.^66–68^ The calculations are performed using the range-separated hybrid functional, CAM-B3LYP,^69^ in conjunction with 6-311++g(d,p) basis set.^70,71^ The cartesian coordinates of the optimized geometries obtained at the CAM-B3LYP level using 6-311++G(d,p) basis set are given at the end of the ESI (Table S8†). Default convergence criteria and parameters as available in the Gaussian 16 program package have been used for all calculations.^65^ The ground state geometries were optimized at the CAM-B3LYP/6-311++g(d,p) level. Single-point energy calculations were performed to determine the vertical excitation energy using the TD-DFT method at the CAM-B3LYP/6-311++g(d,p) level. Geometry optimization of the lowest singlet excited state (S_1_) was carried out using the TD-DFT method at the CAM-B3LYP/6-311++g(d,p). Harmonic vibrational frequency analysis was performed to ensure that the optimized geometries are the minima on the potential energy surfaces. Emission energy has been calculated with zero-point energy correction. The effect of solvation with acetonitrile, carbon tetrachloride, methanol, and water has been investigated using the polarisable continuum model (PCM) based on the integral equation formalism (IEFPCM) variant.^72^

#### 2.1.4 Preparation of samples for solvatochromic study

For the solvatochromic study, a 0.5 mM stock solution of the MPPS molecule was prepared in pure methanol. Then, this solution was poured into a 2 mL sample vial and left for a few hours for complete evaporation of methanol, and then the compound was dissolved in respective solvents with its concentration uniformly maintained at 5 µM. Each sample had a final volume of 2 mL. Lastly, their fluorescence spectra were recorded, and the data was analyzed to investigate the solvatochromic effects of the MPPS molecule in detail.

#### 2.1.5 Preparation of samples for the determination of critical micelle concentration (cmc)

Stock solutions of DTAB (300 mM), SDS (300 mM), Triton-X 100 (5 mM), and 12-6-12,2Br^−^ (10 mM) were prepared in the Milli-Q water. Then, maintaining a constant concentration of 5 µM MPPS from its 0.5 mM methanolic stock solution, the surfactant concentration was subsequently increased for each of the MPPS-surfactant systems. The final volume of each sample was adjusted to 2 mL using the same Milli-Q water. Once the samples are prepared, they are analyzed spectrofluorimetrically to determine the value of each surfactant's critical micelle concentration (cmc).

#### 2.1.6 Preparation of samples for the study with BSA and BSA-surfactant systems

Freshly prepared 10 mM HEPES buffer solution in Milli-Q water was used for the BSA sample preparation after adjusting to pH 7.4. NaOH and H_2_SO_4_ were used for pH adjustments of the buffer solution. Stock solutions of BSA (0.5 mM) and 12-6-12,2Br^−^ (10 mM) were prepared in the pH-adjusted HEPES buffer. Then, each stock solution was carefully degassed for 15 minutes by purging pure nitrogen gas before its usage. For the quenching studies, maintaining BSA at 5 µM, the concentration of MPPS was increased from (0–25) µM from its 0.5 mM methanolic stock solution. This was performed at two different conditions, one in the absence of 12-6-12,2Br^−^ and the other in the presence of 0.3 mM of 12-6-12,2Br^−^. As for the 25 µM MPPS solution, 5% methanol was present, therefore, for all other samples used for the quenching study, 5% methanol was added to make the uniform effect of methanol to the system. Each sample was made up to 2 mL using the HEPES buffer. For the BSA unfolding experiment, BSA and MPPS concentrations were fixed at 5 µM each, and 12-6-12,2Br^−^ was increased in the range of (0–0.9) mM. Each sample was adjusted to 2 mL with a presence of only 1% methanol. Then these samples were utilized for steady-state fluorescence and fluorescence anisotropy, time-resolved fluorescence, and fluorescence anisotropy of MPPS to characterize the unfolding/binding isotherm of 12-6-12,2Br^−^ with BSA. Samples for the other molecule MTPS for measuring intensity decays and lifetimes in BSA at different concentrations of 12-6-12,2Br^−^ were prepared following the same procedures.

#### 2.1.7. UV-visible absorption and fluorescence measurements

Each stock solution underwent meticulous degassing using pure N_2_ gas for 15 minutes before utilization to remove dissolved oxygen. JASCO (Model V-650) UV-vis spectrophotometer was used for absorption measurements of 190–800 nm, and Fluorolog-TM (Horiba Scientific) spectrofluorimeter was used for steady-state emission measurements. A quartz cuvette with a Teflon stopper is used in each case to avoid the evaporation of volatile solvents. Every fluorescence spectrum was corrected for instrument sensitivity and to avoid any possible inner filter effect. The slit width for both slits was set at 3 nm for fluorescence measurements with a fixed scan rate, and the emission range was 340–600 nm with an excitation wavelength in a particular solvent corresponding to a longer wavelength absorption peak maximum of MPPS/MTPS. For some samples, the spectra shoot up if a slit width greater than 3 nm is set. The steady-state anisotropy measurements were carried out on the same spectrofluorimeter using polarizers with excitation and emission wavelengths of 330 and 395 nm, respectively. The time-correlated single photon counting (TCSPC) fluorescence measurements were conducted on a Horiba DeltaFlex™ Modular fluorescence lifetime system. A picosecond diode laser of 330 nm (NanoLED 330L, IBH, UK) was utilized as the light source, having an instrument response function (IRF) of about 177 ps. The fluorescence signals (*λ*_em_ = 395 nm and 365 nm for MPPS and MTPS, respectively) were detected at magic angle (54.7°) polarization using a Picosecond Photon Detection Module (PPD850). A software called EzTime was utilized to analyze the decays. *χ*^2^ criterion significantly gave an overview of the accuracy of the fits while the residuals of the fitted function to the data were visually inspected to reconfirm it. The time-resolved fluorescence anisotropy, *r*(*t*) measurements were carried out on the same TCSPC instrument using polarizers. Other detailed information about the TCSPC measurements is given elsewhere.^73,74^ The fluorescence quantum yields were determined with respect to that of quinine sulfate in 0.1 N H_2_SO_4_ by calculating the area under the fluorescence bands of both MPPS/MTPS in different solvents and quinine sulfate.^19^

#### 2.1.8. Circular dichroism (CD) spectral measurements

The far-UV CD spectra were recorded on Jasco-J 1500 spectropolarimeter in the 190–260 nm wavelength range, maintaining a scan speed of 50 nm min^−1^ and a spectral bandwidth of 2.5 nm for each spectrum. A quartz cuvette with a path length of 0.1 cm was used to record the CD measurements. The background correction was done by subtracting the buffer spectrum from each spectrum recorded for all BSA systems.

All measurements were carried out at room temperature (298.15 ± 1 K).

#### 2.1.9. Molecular docking

A molecular docking study was carried out using a glide module in the Maestro interface of the Schrodinger software (Schrödinger LLC., NY, v2022).^75,76^ The protein selected for the docking study (PDB ID: 4F5S) was downloaded from the protein databank (https://rcsb.org) and prepared using the protein preparation wizard of the software.^77^ The preparation was carried out by adding hydrogens and missing residues using a prime module^78^ and optimizing the 3D structure by minimizing the protein using an Epik module at pH 7.0 ± 2.0.^79^ Since the protein lacks a co-crystal ligand, active site search was carried out using the site-map analysis module of the software, and the highly scored site was used to generate a grid box of 10 Å^3^ dimensions using receptor-grid generation wizard.^75^ The ligands (MPPS and MTPS) were sketched in the 2D sketcher of the maestro interface and prepared using the LigPrep module to obtain a 3D structure, which was further utilized to carry out the molecular docking studies in extra precision (XP) mode^78^ using the optimized potential for liquid simulations 2005 (OPLS_2005) force field.^80^

## 3. Results and discussions

### 3.1. UV-vis absorption and fluorescence study

**Fig. 2:**
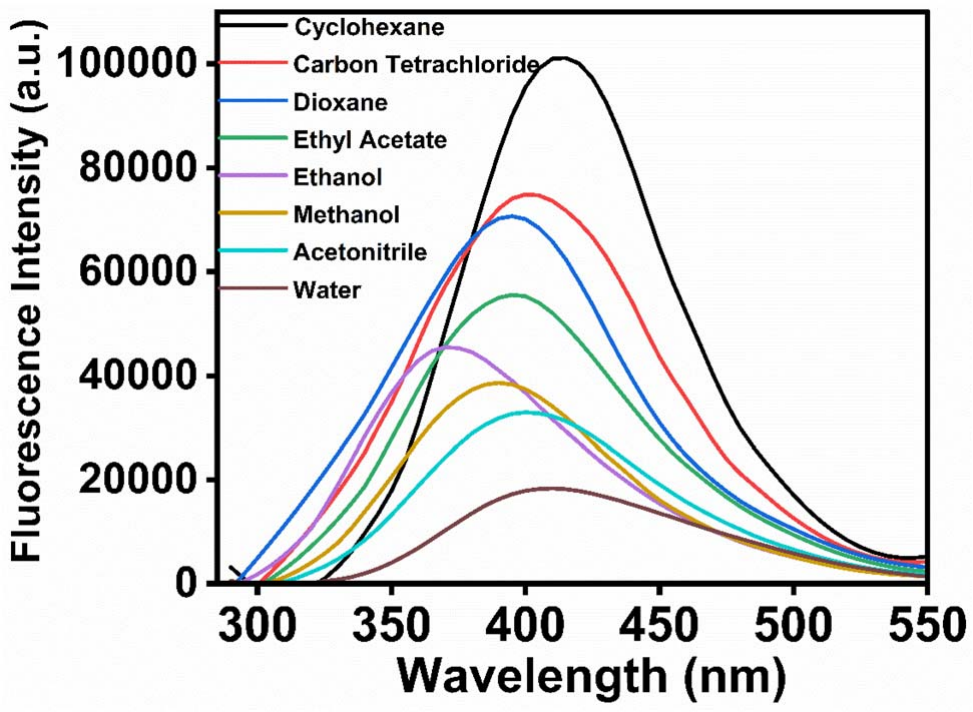
Fluorescence spectra of MPPS in different solvents. [MPPS] = 5.0 µM. *λ*_ex_ for fluorescence measurements of MPPS in a particular solvent corresponds to a longer wavelength absorption peak maximum of MPPS as given in Table 1.

UV-vis absorption spectra of MPPS in solvents of different polarities are recorded and displayed in Fig. S2.† There is a band with a prominent peak at a shorter wavelength, and the other band/shoulder with comparatively lower absorbance appears at a longer wavelength. These two bands correspond to the π → π^*^ and n → π^*^ transitions, respectively, as supported by the theoretical calculations discussed below. The values of peak maxima and the corresponding molar extinction coefficients for shorter and longer wavelength absorption bands are tabulated in Table 1, along with the dielectric constants of solvents. Solvents of three classes are chosen: non-polar (cyclohexane, carbon tetrachloride, dioxane), polar aprotic (ethyl acetate, acetonitrile), and polar protic (ethanol, methanol, water). The general trend is that the longer wavelength absorption peak maxima are blue-shifted with increasing polarity of the solvents. It depicts that the ground state dipole moment is greater than the excited state dipole moment, supported by the results of the theoretical calculations given below. The fluorescence spectra of MPPS in different solvents have been recorded by excitation at corresponding absorption peak maxima of the longer wavelength band and are presented in Fig. 2. The fluorescence peak maxima values are also given in Table 1. Initially, the fluorescence peak maxima are blue-shifted with increasing polarity of the solvents from cyclohexane to ethanol. The fluorescence peak maximum in ethanol is blue-shifted by 42 nm compared to cyclohexane. The fluorescence peak maxima are found to be red-shifted with further increasing polarity of solvents. The *λ*^fl^_max_ of MPPS in water as a solvent is 38 nm red-shifted compared to ethanol as a solvent. The Lippert–Mataga plot of Stokes shifts of fluorophore *versus* the polarity parameters of non-hydrogen bonding solvents is not shown here as that is done in the cases of red-shifts of the fluorescence bands with increasing polarities of solvents which is not the case here. The fluorescence intensities in polar solvents are found to be much lower than that in non-polar solvents. The relative fluorescence quantum yields of MPPS in different solvents have been calculated and presented in Table 1. It can be seen that there is a systematic decrease in fluorescence quantum yield with increasing polarity of the solvents. Compared to cyclohexane as a solvent, the fluorescence quantum yield of MPPS is decreased by 84.35% in water as a solvent. Thus, the significant changes in the fluorescence quantum yields of MPPS with changing the polarity of the solvents, irrespective of which class they belong to, demand the molecule MPPS to be an efficient polarity probe. Notably, low fluorescence in water makes the molecule a potential polarity probe for biological and other microheterogeneous systems because the contribution of fluorescence from the fraction of the probe molecules present in the bulk water would be insignificant. To explain the changes in the fluorescence properties of MPPS with the variation in the polarity of the solvents, electronic structure calculations have been performed at the Density Functional Theory (DFT) and Time-Dependent Density Functional Theory (TD-DFT) levels.

**Table 1:** Polarity parameters (Δ*f*) and dielectric constants (*ε*) of solvents, and absorption peak maxima (*λ*^max^_ab_), fluorescence peak maxima (*λ*^max^_fl_), molar extinction coefficients at absorption peak maxima (log *∈*_max_), fluorescence quantum yields (*ϕ*), lifetimes of fast (*τ*_1_) and slow (*τ*_2_) components, and average lifetime (〈*τ*_f_〉), radiative (*k*_r_) and nonradiative (*k*_nr_) rate constants of MPPS in different solvents. *λ*_ex_ for steady-state fluorescence measurements of MPPS in a particular solvent corresponds to a longer wavelength absorption peak maximum of MPPS. For TCSPC measurements, *λ*_ex_ = 330 nm, and *λ*_em_ = 395 nm

Solvents (Δ*f*)	*ε*	*λ* ^max^_ab_ (nm) (log *∈*_max_)	*λ* ^max^_fl_ (nm)	*ϕ*	*τ*_1_ (ps) (*a*_1_)	*τ*_2_ (ps) (*a*_2_)	〈*τ*_f_〉 (ps)	*χ* ^2^	*k*_r_ (10^8^) s^−1^	*k*_nr_ (10^9^) s^−1^
Cyclohexane (−0.001)	2.02	246 (4.59) 329 (3.17)	414	0.1802	231 ± 8 (0.94 ± 0.05)	1467 ± 32 (0.06 ± 0.02)	305	1.04	5.91 ± 0.78	2.69 ± 0.64
Carbon tetrachloride (0.009)	2.24	254 (4.70) 323 (3.45)	402	0.1716	166 ± 4 (0.91 ± 0.03)	1607 ± 12 (0.09 ± 0.03)	296	0.96	5.80 ± 0.73	2.80 ± 0.67
Dioxane (0.024)	2.25	247 (4.20) 321 (3.43)	396	0.1576	211 ± 22 (0.93 ± 0.03)	1312 ± 32 (0.07 ± 0.02)	288	0.99	5.47 ± 0.63	2.93 ± 0.71
Ethyl acetate (0.201)	6.02	254 (4.52) 318 (3.62)	394	0.1338	177 ± 17 (0.91 ± 0.02)	1173 ± 21 (0.09 ± 0.04)	266	1.14	5.03 ± 0.54	3.26 ± 0.73
Ethanol (0.288)	24.55	228 (4.64) 313 (3.82)	372	0.0926	139 ± 36 (0.96 ± 0.04)	1288 ± 33 (0.04 ± 0.03)	185	1.15	5.01 ± 0.56	4.90 ± 0.79
Methanol (0.308)	32.66	256 (4.38) 312 (3.29)	390	0.0882	123 ± 23 (0.95 ± 0.03)	1220 ± 17 (0.05 ± 0.03)	177	1.18	4.98 ± 0.49	5.15 ± 0.84
Acetonitrile (0.305)	37.50	254 (4.06) 310 (3.04)	402	0.0796	111 ± 30 (0.95 ± 0.03)	1201 ± 18 (0.05 ± 0.04)	165	1.14	4.82 ± 0.48	5.58 ± 0.87
Water (0.319)	78.36	253 (4.62) 307 (3.60)	410	0.0282	87 ± 18 (0.94 ± 0.03)	699 ± 55 (0.06 ± 0.05)	124	1.12	2.27 ± 0.33	7.84 ± 0.92

### 3.2. Theoretical calculations

**Fig. 3:**
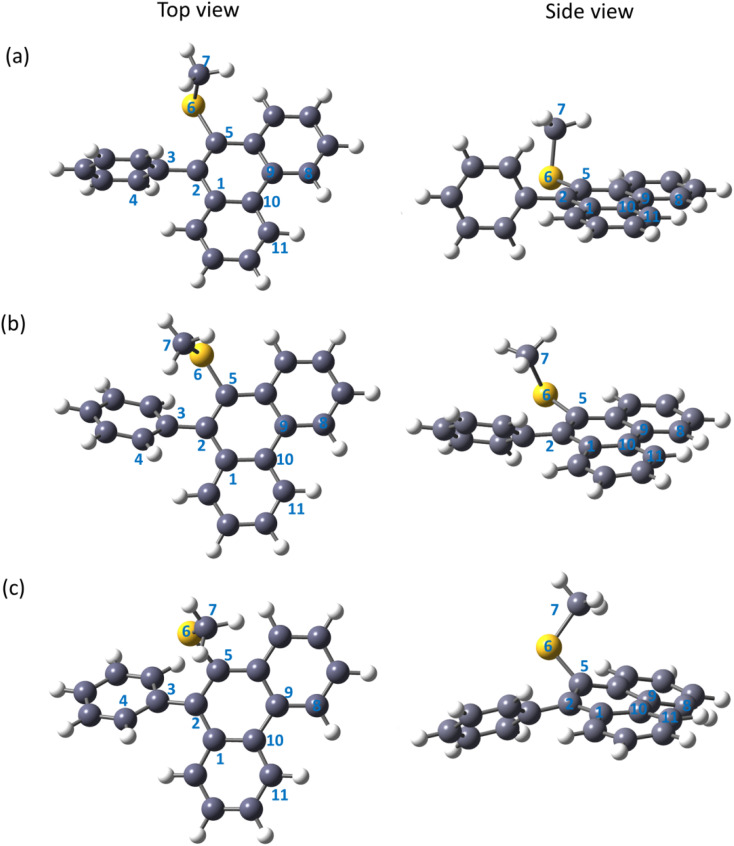
Optimized geometry of the MPPS molecule in the electronic ground state (a), the excited state of conformer E-1 (b), andthe excited state of conformer E-2 (c) in the gas phase obtained at CAM-B3LYP/6-311++g(d,p) level of theory.

The global minimum structure of MPPS in the ground electronic state is presented in Fig. 3(a). The phenanthrene and phenyl rings in the ground electronic state are almost perpendicular to each other with a dihedral angle *φ*_1_, ∠C1–C2–C3–C4 is of 94° in the gas phase. The –S(CH_3_) group is found to be nearly perpendicular to the phenanthrene plane with a dihedral angle *φ*_2_, ∠C2–C5–C6–C7 is of 101°. Two different conformers are obtained as minima in the electronic excited state and are presented in Fig. 3(b) and (c). The lower energy conformer in the excited state (E-1) (Fig. 3(b)) is characterized by *φ*_1_ and *φ*_2_ of 142° and 59°, respectively, and is more stable compared to the other conformer (E-2) by 4 kJ mol^−1^ of energy. The structure of E-2 (Fig. 3(c)) in the gas phase is characterized by *φ*_1_ and *φ*_2_ of 133° and 124°, respectively. The values of dipole moments and dihedral angles of MPPS in both ground and excited states in gas phase and condensed media are tabulated in Table 2. As mentioned, the phenyl and phenanthrene rings are almost perpendicular in the ground state. However, at both E-1 and E-2 states, the dihedral angles between the phenyl and phenanthrene rings have been reduced, taking them towards coplanarity. Though in the gas phase, the E-1 conformer is more stable than the E-2 conformer, under solvation, the situation gets reversed, *i.e.*, E-2 becomes more stable compared to E-1. For example, under solvation in MeOH solvent, the E-2 conformer is found to be 5 kJ mol^−1^ more stable than the E-1 conformer. The variation in the energy difference (Δ*E*) between E-2 and E-1 energy levels and the relative energy of E-1 and E-2 in the gas phase and in the solvents of different polarities are listed in Table S1.† There is an increasing trend in the stabilization of E-1 and E-2 states with increasing solvent polarity with respect to those states in the gas phase. The stabilization in a given solvent compared to the gas phase is higher for E-2 compared to that of E-1. The dihedral angles for MPPS at the excited states (E-1 and E-2) in the gas phase and using PCM in MeOH solvent obtained at the CAM-B3LYP/6-311++g(d,p) level are presented in Table S2.† The ∠C10–C1–C2–C5 and ∠C8–C9–C10–C11 dihedral angle values of the E-1 and E-2 solvated (MeOH) state are presented in Table S2.† These angles specify the planarity of the aromatic phenanthrene ring. In the solvated E-1 state using the CAM-B3LYP functional, the dihedral angle ∠C10–C1–C2–C5 value is −4°, *i.e.*, the carbon atom (C5) is slightly out of a plane to the aromatic ring. Similarly, the benzene ring gets distorted from planarity by 8° as the ∠C8–C9–C10–C11 dihedral angle value is 8°.

**Table 2:** Dipole moments and dihedral angles for MPPS at the ground state and excited state (conformers at E-1 and E-2 states) in the gas phase and using PCM in CCl_4_, ACN, MeOH, and H_2_O solvents obtained using DFT and TD-DFT method at the CAM-B3LYP/6-311++g(d,p) level

Medium		Ground state			Excited state					
					E-1			E-2		
		Dipole moment (D)	Dihedral angle C1–C2–C3–C4 (*φ*_1_)	Dihedral angle C2–C5–S6–C7 (*φ*_2_)	Dipole moment (D)	Dihedral angle C1–C2–C3–C4 (*φ*_1_)	Dihedral angle C2–C5–S6–C7 (*φ*_2_)	Dipole moment (D)	Dihedral angle C1–C2–C3–C4 (*φ*_1_)	Dihedral angle C2–C5–S6–C7 (*φ*_2_)
Gas	S_1_ state	1.400	94°	101°	1.191	142°	59°	0.977	133°	124°
PCM	CCl_4_	1.647	92°	102°	1.414	140°	59°	1.101	132°	142°
	MeOH	2.145	90°	104°	1.783	134°	52°	1.537	134°	139°
	ACN	2.150	90°	104°	1.787	134°	67°	1.541	134°	139°
	H_2_O	2.183	90°	104°	1.802	134°	50°	1.570	135°	140°

Absorption and emission energies have been obtained from the theoretical calculations (Table 3) and compared with the experimental results (Table 1). In the gas phase, the theoretical excitation energy is 4.26 eV. The absorption maximum in CCl_4_ is the same as in the gas phase. The absorption maxima are slightly blue-shifted (4.27 eV) in the other three solvents, MeOH, ACN, and water, compared to the gas phase/CCl_4_ absorption maximum. Experimentally, the absorption peak maxima are blue-shifted with increasing polarity of the solvents (Table 1). This is because the ground state dipole moments of MPPS in all solvents are higher than those in the excited state, and the dipole moment increases with increasing polarity of the solvents (Table 2). Thus, the theoretical results are in reasonable agreement with the experimental results.

**Table 3:** Theoretical excitation energy (Ex), oscillator strength (*f*), and emission energies of MPPS for E-1 and E-2 conformers in the gas phase and using PCM in CCl_4_, MeOH, ACN, and H_2_O solvents obtained using TD-DFT method at the CAM-B3LYP/6-311++g(d,p) level

Medium			Excitation energy (Ex) and oscillator strength (*f*)		Emission energy	
		Dielectric constants (*ε*)	Ex eV (nm)	*f*	E-1 eV (nm)	E-2 eV (nm)
Gas	S_1_ state	—	4.26 (291)	0.001	3.69 (336)	3.73 (332)
PCM	CCl_4_	2.24	4.26 (291)	0.002	3.68 (337)	3.68 (337)
	MeOH	32.66	4.27 (290)	0.002	3.64 (341)	3.59 (345)
	ACN	35.95	4.27 (290)	0.003	3.64 (341)	3.59 (345)
	H_2_O	78.36	4.27 (290)	0.003	3.64 (341)	3.58 (346)

The theoretical results provide two emission wavelengths corresponding to the two conformers in the electronically excited state. The emission energies of the E-1 and E-2 conformers in the gas phase are 3.69 eV (*λ* = 336 nm) and 3.73 eV (*λ* = 332 nm), respectively (Table 3). As stated above, the E-1 conformer has lower energy than the E-2 conformer in the gas phase; however, in the condensed phase, it is reversed, and the E-2 has lower energy than the E-1. The emission energies of both the E-1 and E-2 conformers are red-shifted under solvation in CCl_4_, MeOH, ACN, and water solvents, and this red-shift increases with increasing polarity of the solvents. Experimentally observed fluorescence peak maxima are in a trend as follows: there is a blue shift from cyclohexane to ethanol as solvents followed by a red shift from methanol to water as solvents (Table 1). To support the fact that the emission occurs from two different emitting states, the deconvolutions of fluorescence spectra of MPPS have been done and shown for a few solvents as representatives in Fig. S3.† It can be seen that in each case, the fluorescence band is a combination of two bands. Table S3† presents the peak maxima of two bands in solvents of different polarities obtained after deconvolution of experimental fluorescence bands. One can see that both the fluorescence bands are blue-shifted till ethanol compared to cyclohexane and then red-shifted with further increasing polarity of solvents.

Experimentally, it is observed that the fluorescence quantum yield decreases with increasing polarity of the solvents (Table 1). To explain this fact, the dihedral angles, *φ*_1_ and *φ*_2_ of MPPS have been calculated in the solvents of different polarities in both ground and excited states (E-1 and E-2 conformers), and are tabulated in Table 2. It can be seen that for the E-1 conformer, *φ*_1_ reduces, whereas, for the E-2 conformer, *φ*_1_ increases with an increase in the polarity of the solvent. Therefore, for the conformer in the E-1 state, the coplanarity between the phenyl and phenanthrene rings is reduced in a polar medium. Therefore, the deactivation of the E-1 excited state of the molecule through non-radiative processes occurs, which is enhanced by increasing the solvent's polarity. Though for the E-2 conformer, the coplanarity is increased with increasing polarity, the quantum yield for the combined emission process is calculated. Thus, the fluorescence intensity is reduced with an increase in the polarity of the solvent.^4^ To estimate the radiative (*k*_r_) and non-radiative (*k*_nr_) rate constants of MPPS, and the effect of solvent's polarity on these processes, the fluorescence lifetimes of MPPS in different solvents have been measured. Fluorescence intensity decays in some solvents are shown in Fig. S4.† In each case, it is noted that the intensity decay is bi-exponential, with major fast and minor slow components supporting two species in the excited state. The lifetime values of the fast and the slow components, the corresponding weightings, and the average lifetime calculated using eqn S1 (ref. 81) in Note S1† have been given in Table 1. *k*_r_ and *k*_nr_ values have been calculated using eqn S2 (ref. 81) and S3 (ref. 81) in Note S1,† respectively. It can be seen that with increasing polarity of solvents, the *k*_r_ value decreases, and the *k*_nr_ value increases with a systematic decrease in the lifetimes of the fast component, supporting our explanation based on the theoretical results that the rate of nonradiative process increases with decreasing coplanarity.

**Fig. 4:**
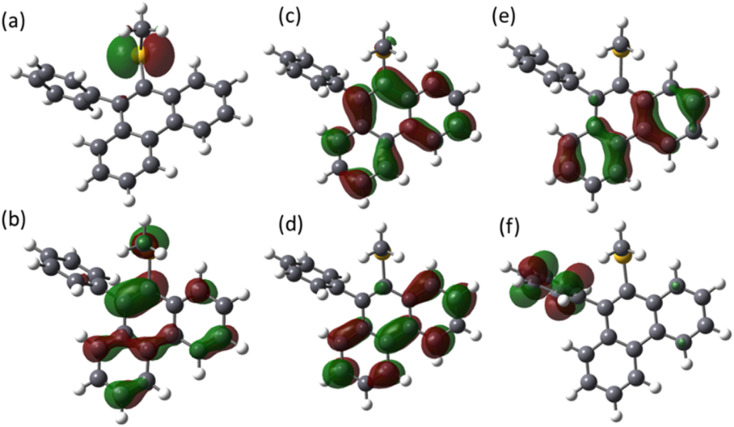
The frontier molecular orbitals (FMO) of the MPPS in the electronic ground state: (a) HOMO, (b) LUMO, (c) HOMO−1, (d) LUMO+1, (e) HOMO−2, and (f) LUMO+2, obtained at CAM-B3LYP/6-311++g(d,p) level of theory.

The molecular orbitals of MPPS are presented in Fig. 4. HOMO is characterized as the lone pair orbital on the sulfur atom of the –S(CH_3_) group. The LUMO (π*) is majorly delocalized over the phenanthrene ring of the molecule. The theoretical excitation energies, corresponding wavelengths, oscillator strengths, and configurations of excited states are listed in Table 4. It has been observed that all the excitations are due to the combined contribution of multiple orbital transitions. The transition from the S_0_ state to the S_1_ state involves two orbital transitions, HOMO to LUMO+1 (n to π*), HOMO−1 to LUMO (π to π*), and HOMO−2 to LUMO (π to π*). The contributions of transitions are 45%, 26%, and 18%, respectively. This transition has a very low oscillator strength of 0.001 with an absorption energy of 4.26 eV. The transition from the S_0_ to the S_2_ state has a theoretical absorption energy of 4.41 eV. This band is mainly associated with the HOMO → LUMO configuration with a contribution of 56%, indicating a transition from n to π* orbital with an oscillator strength of 0.07. The lower value of oscillator strength indicates that the n → π* type of transition is expected to be less intense. The other transitions from the S_0_ to S_2_ state are HOMO−1 → LUMO (π to π*) and HOMO−2 → LUMO (π to π*), with contributions of 15% and 8%, respectively. The excitation to the S_3_ state consists of multiple orbital transitions, such as HOMO−2 → LUMO (π to π*), HOMO−1 → LUMO (π to π*), HOMO → LUMO (n to π*), HOMO−1 → LUMO (π to π*), and HOMO−2 → LUMO+1 (π to π*). The oscillator strength of S_0_ to S_3_ is 0.04. The excitation S_0_–S_5_ consists of two kinds of orbital excitation features, HOMO → LUMO (n to π*) and HOMO−1 → LUMO+1, HOMO−2 → LUMO+1 (π to π*). The oscillator strength of S_0_ to S_5_ (*f* = 0.73) is higher compared to the S_0_ to S_4_ (*f* = 0.33) and S_0_ to S_3_ (*f* = 0.04) transitions. The calculated oscillator strength values are in consistent with the experimentally found molar extinction coefficients (log *∈*_max_) (Table 1). The log *∈*_max_ value of MPPS for the long wavelength n → π* transition is smaller than that for the shorter wavelength π → π* transition in each solvent.

**Table 4:** Theoretical excitation energy *E* (eV), wavelength *λ* (nm), oscillator strength (*f*), and electronic configurations for MPPS in the gas phase obtained using the TD-DFT method at the CAM-B3LYP/6-311++g(d,p) level. The contribution in the percentage of the transitions is written in parentheses

State	*E* (eV)	*λ* (nm)	*f*	Configuration
S_1_	4.26	290	0.001	HOMO → LUMO+1 (45%)
				HOMO−1 → LUMO (26%)
				HOMO−2 → LUMO (18%)
				HOMO−2 → LUMO+1 (2%)
S_2_	4.41	281	0.07	HOMO → LUMO (56%)
				HOMO−1 → LUMO (15%)
				HOMO−1 → LUMO+1 (4%)
				HOMO−2 → LUMO (8%)
				HOMO−2 → LUMO+1 (5%)
S_3_	4.53	273	0.04	HOMO−2 → LUMO (32%)
				HOMO−1 → LUMO (27%)
				HOMO → LUMO (16%)
				HOMO−1 → LUMO+1 (7%)
				HOMO−2 → LUMO+1 (5%)
S_4_	5.16	240	0.33	HOMO → LUMO+1 (47%)
				HOMO−2 → LUMO (29%)
				HOMO−1 → LUMO (18%)
S_5_	5.20	238	0.73	HOMO−1 → LUMO+1 (39%)
				HOMO−2 → LUMO+1 (23%)
				HOMO → LUMO (17%)

To support the fact that the free rotation of the phenyl ring enables the molecule to change the dihedral angle between the phenyl and phenanthrene rings by changing the polarity that controls the electronic energy, and fluorescence lifetime (experimental data discussed above and below), *etc.*, we have synthesized another molecule, methyl(10-(*o*-tolyl)phenanthren-9-yl)sulfane (MTPS) (Scheme S1†) and done theoretical calculations. MTPS is the derivative of MPPS wherein a –CH_3_ group is present in the *ortho* position of the phenyl ring of the MPPS. Unlike MPPS, for MTPS, two ground state conformers (G-1 and G-2) are obtained (Fig. S5a and b,† respectively), and one excited state conformer (E-1) is obtained (Fig. S5c†). All geometries are optimized at CAM-B3LYP/6-311++g(d,p) level of theory. The values of dihedral angles ∠C1–C2–C3–C4 (*φ*_1_) and ∠C2–C5–C6–C7 (*φ*_2_) for the conformers of MTPS in G-1, G-2, and E-1 states in the gas phase and using PCM in CCl_4_, MeOH, ACN and water solvents obtained using CAM-B3LYP/6-311++g(d,p) level are tabulated in Table S4.† Notable observations are as follows: (1) *o*-tolyl (substituted phenyl) and phenanthrene rings are almost perpendicular in the ground states, G-1 and G-2, in the gaseous and condensed phases, (2) the E-1 conformer in the electronic excited state is characterized by *φ*_1_ and *φ*_2_ of 140° and 62°, respectively in the gas phase. All efforts to optimize the other structure by changing the relative position of the –CH_3_ group always lead to the E-1 structure, and (3) a single conformer in the excited state is also obtained in the condensed phase. Though the dihedral angle, *φ*_1_ in CCl_4_ is almost the same as that in the gas phase (139°), in the polar aprotic (ACN) and polar protic solvents (MeOH and water), this angle is found to be 74°. The coplanarity between the *o*-tolyl and phenanthrene rings in MTPS is less than in MPPS, which says that the free rotation of the *o*-tolyl (substituted phenyl) ring is relatively restricted in MTPS. Fig. S6† presents the fluorescence spectra of MTPS in the solvents of different polarities. Notably, no extra band is found after deconvolution of fluorescence spectra. The table in Fig. S6† displays the absorption and fluorescence peak maxima and fluorescence quantum yields of MTPS in the solvents of varying polarities. Like MPPS, the fluorescence peak maxima are blue-shifted with increasing polarity up to ethanol and then red-shifted from methanol to water. The quantum yield of MTPS in a given solvent is higher than that of MPPS, supporting the fact that free rotation about the C–C bond is responsible for low fluorescence quantum yield. Table S5† presents theoretical excitation energies for G-1 and G-2 conformers and emission energies (*E*) of MTPS in the gas phase and using PCM in CCl_4_, ACN, MeOH, and water solvents. The emission energies are red-shifted with increasing polarity of solvents.

To present the molecule, MPPS as a potential polarity probe, it has been explored to characterize the micelles of different surfactants (both conventional and gemini surfactants), protein, bovine serum albumin (BSA), and BSA-gemini surfactant binding isotherm.

### 3.3. Binding of MPPS with micelles of different surfactants

**Fig. 5:**
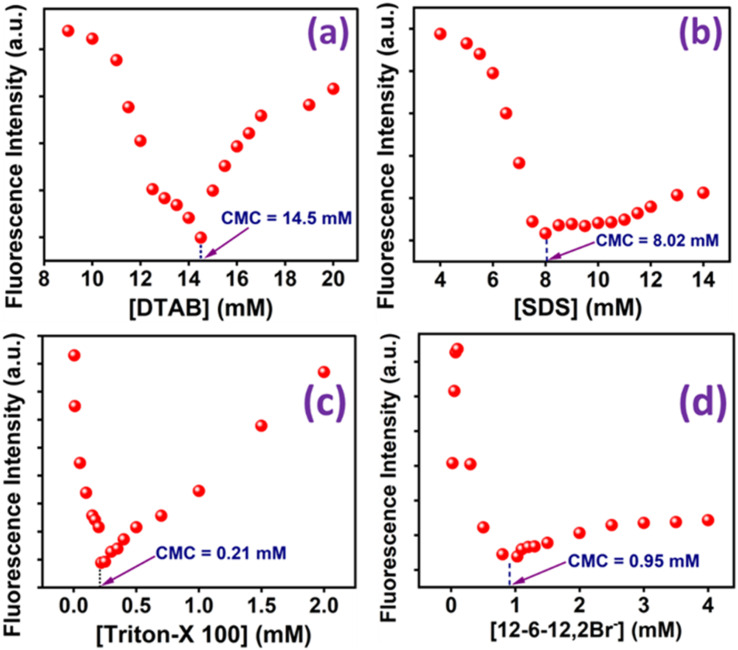
Fluorescence intensities of MPPS at different concentrations of (a) DTAB, (b) SDS, (c) Triton-X 100, and (d) 12-6-12,2Br^−^. [MPPS] = 5 µM, *λ*_ex_ = 330 nm and *λ*_em_ = 395 nm.

Fluorescence spectra (not shown here) of MPPS have been recorded at different concentrations of each of three conventional surfactants: dodecyl trimethylammonium bromide (DTAB), sodium dodecyl sulfate (SDS), Triton X-100, and a gemini surfactant, 12-6-12,2Br^−^ at *λ*_ex_ = 330 nm. These are monomeric cationic, anionic, non-ionic, and dimeric cationic surfactants, respectively. In each case, the fluorescence intensities of MPPS at different concentrations of surfactant have been calculated at 395 nm and are plotted in Fig. 5a–d, respectively. It can be seen from Fig. 5a–d that the fluorescence intensity decreases reaches a minimum, and then increases with increasing concentration of each surfactant. The minimum appears at [DTAB] = 14.5 mM, [SDS] = 8.02 mM, [Triton X-100] = 0.21 mM, and [12-6-12,2Br^−^] = 0.95 mM. These concentrations are critical micellar concentrations (cmc) of the respective surfactants. The values are close to the reported cmc values of 14.7 mM,^82,83^ 8.20 mM,^82^ 0.25 mM,^28^ and 1.03 mM.^60^ for DTAB, SDS, Triton X-100, and 12-6-12,2Br^−^, respectively. Results thus depict that the fluorescence property of MPPS is very sensitive to the changes in the microenvironment around it. In the pre-micellar region, each ionic surfactant acts as a strong electrolyte. Therefore, with increasing surfactant concentration, the number of ions gradually increases, resulting in increasing polarity of the medium. That is why, in this region, the fluorescence intensity of MPPS decreases with increasing surfactant concentration, as supported by the solvatochromic results. In the case of Triton-X 100, in the pre-micellar region, the fluorescence intensity is reduced, which could be because of the interactions between the –OH groups of the surfactant molecules and MPPS, before the MPPS molecules trapped inside the core of the micelles in the post-micellar region. However, once the micelles start to form above the cmc, the fluorescence intensity of MPPS increases because the molecules move from the polar bulk to the non-polar sites of the micelles. The polarity of the microenvironment around MPPS decreases due to the solubilization of the MPPS in the micelles.

Notably, the pyrene molecule has been widely used to determine the cmc of various surfactants.^83–86^ The ratio *I*_3_/*I*_1_ (where *I*_3_ and *I*_1_ are fluorescence intensities at the third (384 nm) and first (374 nm) vibrational emission peaks, respectively) of pyrene depends on the polarity of the environment. In all cases, the plot of *I*_3_/*I*_1_*versus* [surfactant] gives a sigmoid curve, which is not very handy for estimating the cmc. There are several other fluorescent probes used for the determination of the cmc of surfactants,^87–89^ and biomolecule^90^ which are also reported to present sigmoid curves. However, with the present fluorescent probe MPPS, the changes in fluorescence intensities with [surfactant] are found to be V-shaped in all types (cationic, anionic, and non-ionic) of conventional surfactants and a gemini surfactant which is very rare.^4,86^ We could measure the cmc values precisely from the minima of the plots, which counts as an advantage.

Other than fluorescence intensities, the changes in full width at half the maximum (FWHM) of a fluorescence band with varying concentrations of surfactants have been calculated and plotted in Fig. S7.† Interestingly, the minimum in the plot of FWHM (in cm^−1^) *versus* [surfactant] is obtained exactly at the same cmc mentioned above for each surfactant. Initially, in the pre-micellar region, FWHM increases due to the heterogeneity in the locations of MPPS. Just before cmc, FWHM drops due to gradual homogeneity in the locations of MPPS as a result of the solubilization of MPPS in micelles. Above cmc, again, the heterogeneity increase could be because of the formation of micelles of varying sizes with increasing surfactant concentrations. At a very high concentration, FWHM again falls, which could be due to changes in the shapes of micelles.

### 3.4. Probing microenvironment of tryptophan residues of the protein, bovine serum albumin (BSA) using MPPS

**Fig. 6:**
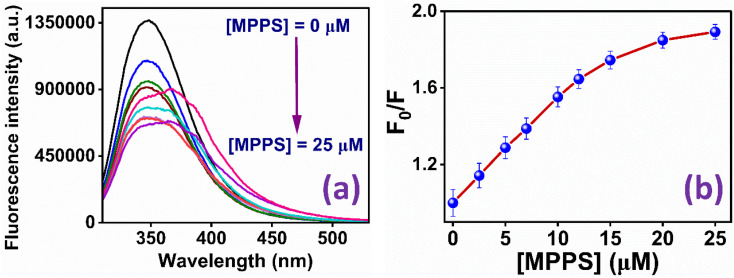
(a) Fluorescence spectra of BSA in the presence of different concentrations of MPPS; (b) Stern–Volmer plot for the quenching of fluorescence of BSA by MPPS. *λ*_ex_ = 295 nm, *λ*_em_ = 350 nm.

The binding of the molecule with BSA has been studied to explore MPPS as a fluorescent probe for a biomolecule. The fluorescence spectra of native BSA have been recorded at *λ*_ex_ = 295 nm at different concentrations of MPPS (0 to 25 µM) and are shown in Fig. 6a. Notably, the excitation of BSA at 295 nm results in fluorescence from the protein's tryptophan (Trp) residues. It can be seen in Fig. 6a that with increasing concentration of MPPS, the fluorescence intensity of BSA decreases, and that of MPPS increases. Therefore, MPPS acts as a quencher. As MPPS also has a little absorption at 295 nm, fluorescence from both BSA and MPPS is seen in this figure. While the fluorescence peak maximum of BSA appears at ∼350 nm, the same for a fluorescence band of MPPS is seen at ∼363 nm. Fig. 6b displays the changes in fluorescence intensity ratios, *F*_o_/*F* (where *F*_o_ and *F* are fluorescence intensities of BSA in the absence and presence of MPPS, respectively) of BSA with increasing concentrations of MPPS. This Stern–Volmer plot shows a downward curvature indicating the fractional accessibility of Trp residues of BSA to the quencher, MPPS (Fig. 6b). Two different fractions of Trp residues, Trp-213 and Trp-134, exist in BSA.^91^ Trp-213 is located in a hydrophobic pocket, and Trp-134 resides in a hydrophilic environment *i.e.*, near the surface of the protein.^91^ Docking results (discussed below) show that MPPS binds to the hydrophobic region of the BSA. Therefore, it is expected that MPPS will be more accessible to Trp-213 than Trp-134. In our earlier study, a blue shift in fluorescence peak maximum of BSA was noted with the increasing concentration of an extrinsic fluorescent probe, dimethylaminopstyryl benzothiazole (DMASBT).^13^ It is due to the accessibility of Trp-134 to DMASBT, as the latter binds to the hydrophilic sites of BSA. As the fluorescence from Trp-134 occurs slightly at a longer wavelength than that of Trp-213, thus with the quenching of fluorescence from Trp-134, the overall peak maximum of BSA gets blue-shifted. However, in the present case, the blue shift in BSA fluorescence peak maximum with increasing concentration of MPPS is not seen, supporting that MPPS quenches fluorescence from Trp-213, not from Trp 134.

**Fig. 7:**
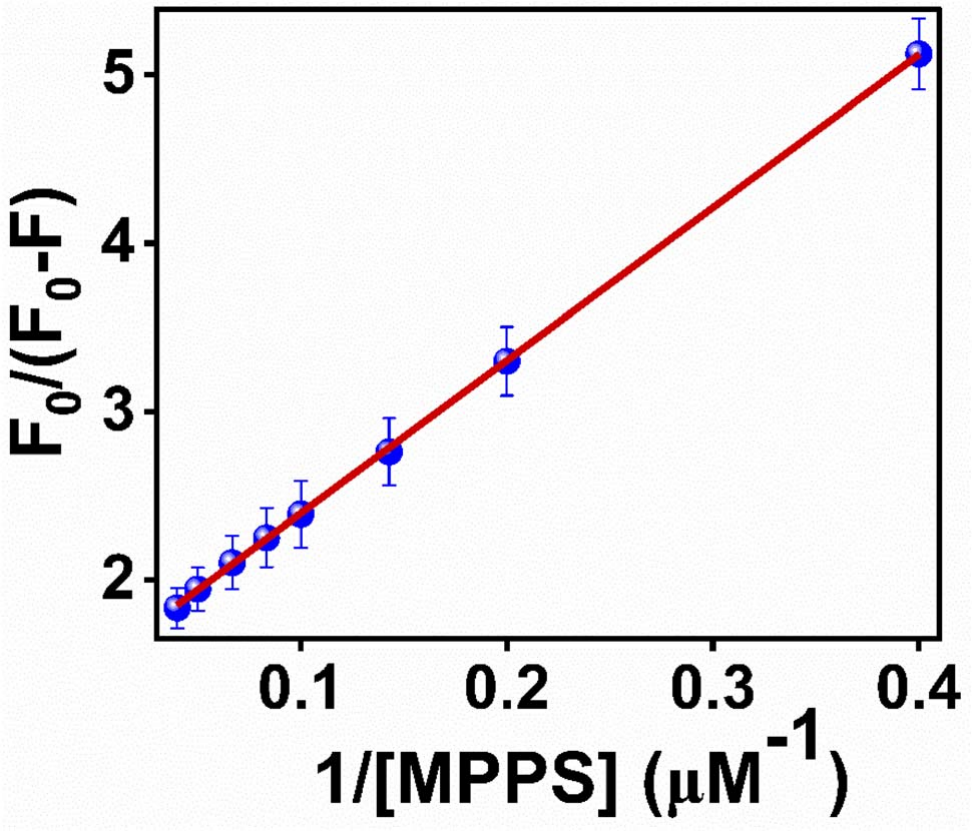
Modified Stern–Volmer plot for the quenching of Trp residues in native BSA by MPPS. *λ*_ex_ = 295 nm, *λ*_em_ = 350 nm.

To quantify the fractional accessibility of Trp residues to MPPS in native BSA, and to calculate the values of the fraction of Trp residues accessible to MPPS (*f*_a_) and the Stern–Volmer constant (*K*_a_) for the quenching of accessible fractions in the native protein, the modified Stern–Volmer plot based on eqn S4 (ref. 81) in Note S1† has been made and shown in Fig. 7. The correlation coefficient of this modified Stern–Volmer plot is found to be 0.999. The mathematical form of *f*_a_ has been shown by eqn S5 (ref. 81) in Note S1.†*f*_a_ and *K*_a_ values calculated from the intercept and the slope of the modified Stern–Volmer plot after linear fitting of the data are found to be 0.68 and 1.63 × 10^8^ M^−1^, respectively. Thus, 68% of Trp residues are only accessible to the MPPS in native BSA. The Stern–Volmer quenching constant of the accessible fraction, *K*_a_ = *k*_q_*τ*_o_ = 1.63 × 10^8^ M^−1^. Here, *k*_q_ is the bimolecular quenching rate constant, and *τ*_o_ is the lifetime of BSA in the absence of any quencher. The *τ*_o_ value for BSA protein is reported to be ∼10^−8^ s.^92–94^ Thus, the *k*_q_ value is calculated to be 1.63 × 10^16^ M^−1^ s^−1^. However, the maximum value of *k*_q_ for a diffusion-controlled fluorescence quenching process with a biopolymer is known to be ∼2.0 × 10^10^ M^−1^ s^−1^.^85^ It depicts that the fluorescence quenching mechanism of the accessible fraction of Trp residues is static in nature.

**Fig. 8:**
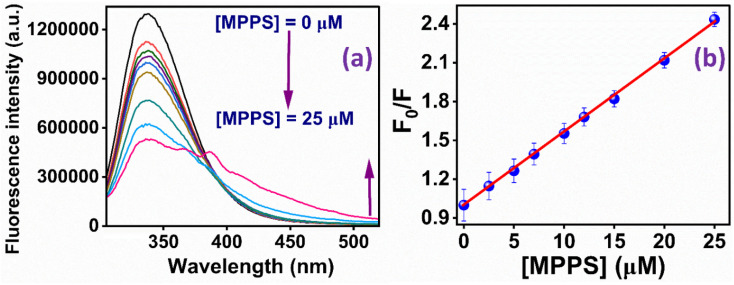
(a) Fluorescence spectra of BSA with increasing concentrations of MPPS in the presence of 0.3 mM of 12-6-12,2Br^−^; (b) Stern–Volmer plot for the quenching of fluorescence of BSA by MPPS in the presence of 0.3 mM of 12-6-12,2Br^−^. *λ*_ex_ = 295 nm, *λ*_em_ = 337 nm.

The fluorescence quenching of BSA by MPPS has also been studied in denatured protein. The denaturation of BSA has been done by using 0.3 mM of 12-6-12,2Br^−^. It has been discussed below that at 0.3 mM concentration of 12-6-12,2Br^−^, BSA exists in denatured form. Fig. 8a displays the fluorescence bands of BSA at varying concentrations of MPPS (0 to 25 µM) in the presence of 0.3 mM of 12-6-12,2Br^−^. The Stern–Volmer plot obtained using fluorescence quenching data is shown in Fig. 8b. The correlation coefficient of this plot = 0.993. Interestingly, no downward curvature is noted in this Stern–Volmer plot depicting equal accessibility of all Trp residues of BSA to the quencher, MPPS. It is expected because the BSA unfolds in the presence of surfactant, and therefore, both Trp residues are exposed to the bulk. The Stern–Volmer constant calculated from the slope of the plot after linear fitting of the data = 1.63 × 10^8^ M^−1^. Therefore, the bimolecular quenching rate constant, *k*_q_ = 1.63 × 10^16^ M^−1^ s^−1^. These results show that the MPPS can potentially probe the localization of intrinsic fluorophores in a protein and the changes in their microenvironment upon denaturation.

The binding constant (*K*′) of MPPS and the number of binding sites (*n*) for both native and denatured BSA have been calculated from the linear fitting of the data in Fig. S8a and b based on eqn S6 (ref. 94) in Note S1,† respectively. For native BSA, the *K*′ and n values are noted to be 2.75 × 10^5^ M^−1^ and 0.57, respectively. Whereas for denatured BSA, the *K*′ and n values are found to be 4.41 × 10^4^ M^−1^ and 0.91, respectively. As compared to native BSA, the binding constant value is decreased, and the number of binding sites is increased for denatured BSA. This is because, in the denatured state of the protein, the binding strength of MPPS molecules is reduced as the hydrophobic area of the protein is decreased.^95^ It is evidenced by the fluorescence peak maximum values of MPPS in two systems: *λ* = ∼350 nm in the presence of native BSA and *λ* = ∼337 nm in the presence of BSA and 0.3 mM concentration of the gemini surfactant, 12-6-12,2Br^−^. The blue shift in the band position of MPPS in the case of denatured BSA is due to its exposure to a comparatively more polar environment. Also, the possibility of binding sites may increase with the protein unfolding, as noted.

### 3.5. Constructing binding isotherm of gemini surfactant, 12-6-12,2Br^−^ with BSA using MPPS as a probe

#### 3.5.1. Steady-state fluorescence of MPPS

**Fig. 9:**
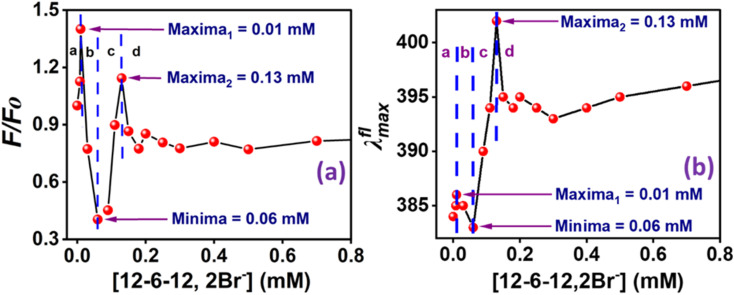
Plots of (a) *F*/*F*_O_ and (b) *λ*^fl^_max_ of MPPS in BSA at different concentrations of 12-6-12,2Br^−^. [MPPS] = 5.0 µM, [BSA] = 5.0 µM. *λ*_ex_ = 330 nm, *λ*_em_ = 395 nm.

Since the fluorescence properties of MPPS respond well to the changes in the microenvironment because of what the cmc of different surfactants and the locations of Trp residues of BSA could be measured, the molecule is explored to demonstrate the binding isotherm of a gemini surfactant, 12-6-12,2Br^−^ with BSA. The fluorescence spectra of MPPS in 5.0 µM of BSA have been recorded at different concentrations of 12-6-12,2Br^−^ at *λ*_ex_ = 330 nm. No absorbance for BSA at *λ* = 330 nm ensures that the fluorescence occurs from MPPS only. The fluorescence intensity ratios, *F*/*F*_o_ (where *F* and *F*_o_ are fluorescence intensities of MPPS in the presence and absence of surfactant at *λ*_em_ = 395 nm) at different concentrations of 12-6-12,2Br^−^ have been calculated and plotted in Fig. 9a. The different regions of the binding isotherm have been depicted by letters *a*, *b*, *c*, and *d*. Region *a* represents the high-energy specific binding of the surfactant with BSA due to coulombic attractive interactions between positive charges of surfactants' headgroups and negative charges on the protein. As because the BSA has become more compact in this region,^96–98^ the MPPS feels a more non-polar environment in the hydrophobic pocket of BSA. As a result, the fluorescence intensity is increased. It has been observed experimentally and supported by theory (discussed above) that the fluorescence intensity of MPPS is increased with decreasing solvent polarity. The transfer of MPPS to a comparatively less polar environment in this region is also supported by the red shift in the fluorescence peak maximum as shown in Fig. 9b. The red shift in the fluorescence peak maximum with decreasing polarity and the blue shift in the peak maximum with increasing polarity of the medium (except for a few highly polar solvents) have been noted experimentally and theoretically, as discussed above. In this region, a maximum fluorescence intensity appears at [12-6-12,2Br^−^] = 0.01 mM.

**Fig. 10:**
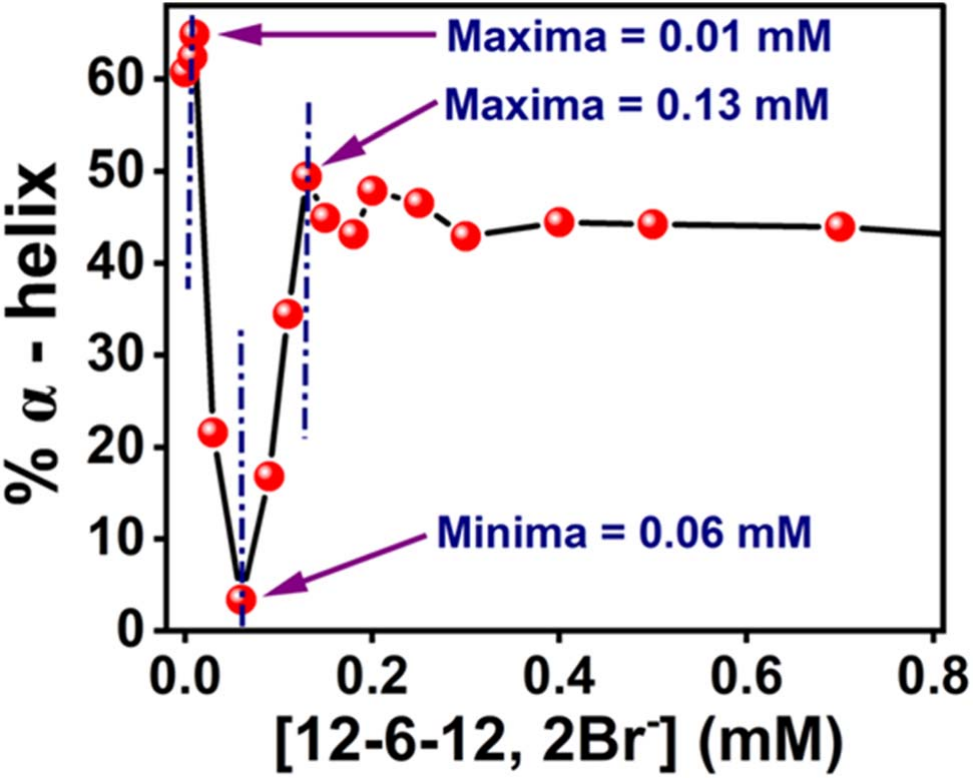
Changes in % α-helix of BSA with increasing concentration of 12-6-12,2Br^−^ in the presence of MPPS. [MPPS] = 5.0 µM, [BSA] = 5.0 µM.

The Far-UV CD spectra with increasing concentrations of 12-6-12,2Br^−^ have been recorded. In the case of native BSA, the CD spectrum displays two distinctive negative bands at 208 and 222 nm, indicative of its α-helical structure^99^ with an α-helical content of 60.7%, aligning well with the literature reports.^100,101^ It suggests a 60–67% range for native BSA's α-helix content. To quantitatively assess the α-helical, β-sheet, and random coil components of BSA's secondary structure, analysis was performed using BestSel software within the 200–260 nm range, and the data obtained are given in Table S6.† In region *a*, as a result of the compaction of BSA, the % α-helix is increased, which can be seen in Fig. 10. With further increasing concentration of 12-6-12,2Br^−^, the fluorescence intensity starts decreasing (Fig. 9a) with a blue shift in peak maximum (Fig. 9b) before reaching a minimum at 0.06 mM of 12-6-12,2Br^−^ (region *b*). The region *b* represents non-cooperative (or competitive) binding between the surfactants and BSA. In this region, the hydrophobic interactions between the surfactant's tails and the non-polar part of the protein mostly play a role.^96^ The protein molecule unfolds with a decrease in % α-helix (Fig. 10) and an increase in β-sheet content (Table S6†) due to these interactions, and as a result, the probe MPPS gets exposed to the polar environment. Thus, the fluorescence intensity is quenched with a blue shift in peak maximum (Fig. 9b). The quenching of fluorescence intensity in the polar environment has been noted experimentally and is supported theoretically as discussed above. Beyond 0.06 mM of 12-6-12,2Br^−^, the fluorescence intensity of MPPS increases (region *c*) before reaching a maximum of 0.13 mM of the surfactant. Region *c* exists due to the cooperative binding wherein the micelles formed by surfactants bind along the protein chain, forming a necklace-bead kind of structure.^20,96^ However, the probe molecules, MPPS, experience a non-polar environment of hydrophobic microdomain created by micelles of surfactants.^96,97^ The hydrophobic microdomain that is created within the protein molecule results in the compaction of the protein structure,^96,97^ which is supported by the increase in the % of α-helix^13^ (Fig. 10) along with a decrease in β-sheet content (Table S6†). Notably, micelle formation starts at 0.06 mM concentration of 12-6-12,2Br^−^, which is much lower than its cmc value for a solution without BSA. Similar results were obtained in our earlier work with the gemini surfactants, 12-4-12,2Br^−^ and 12-8-12,2Br^−^ utilizing some other fluorescent probe molecule.^13^ Thus, 0.06 mM is the critical aggregation concentration (cac) of 12-6-12,2Br^−^ in the HEPES buffer solution (pH = 7.4, 10 mM) in the presence of 5.0 µM of BSA. In region *d*, due to the massive binding of surfactants with the protein, the protein molecules get unfolded, and as a consequence, the fluorescence intensity of MPPS is once again decreased due to its exposure to the polar medium with a decrease in % of α-helix (Fig. 10). With further increases in the concentration of 12-6-12,2Br^−^, no change in the fluorescence intensity occurs as no sites of the protein chain are left for binding, and saturation of surfactant binding occurs. Thus, the MPPS molecule has proved to be an excellent polarity probe for the protein, showing changes in the fluorescence properties with alterations in the microenvironment.

#### 3.5.2. Steady-state fluorescence anisotropy of MPPS

**Fig. 11:**
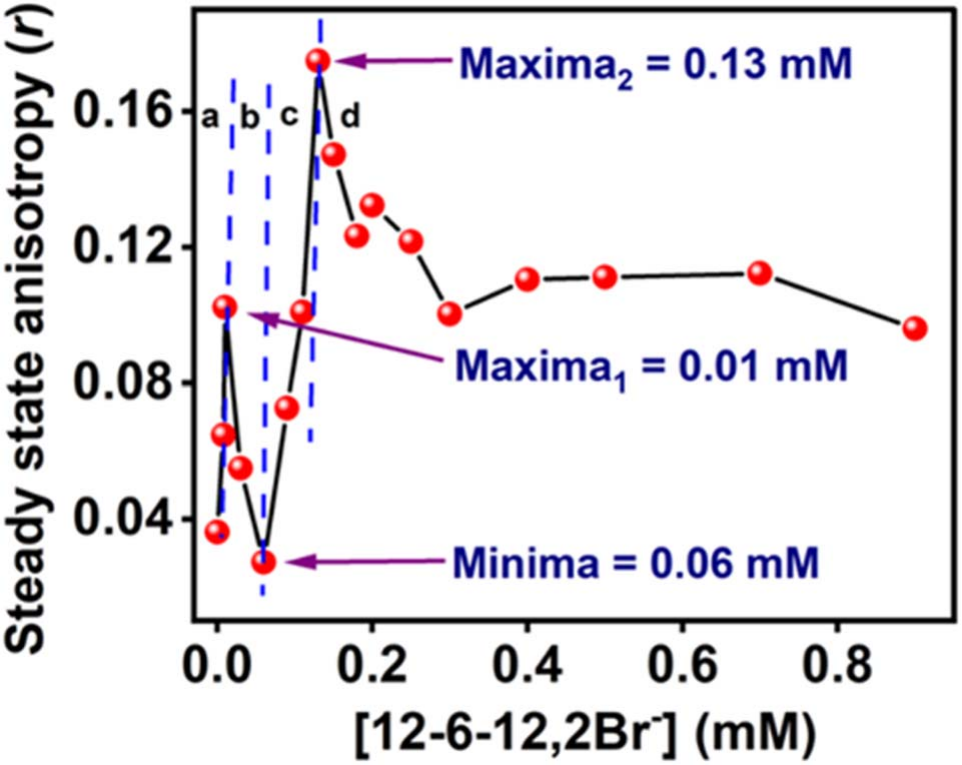
Steady-state anisotropy (*r*) plot of MPPS in the presence of BSA along with increasing concentration of 12-6-12,2Br^−^. [MPPS] = 5.0 µM, [BSA] = 5.0 µM. *λ*_ex_ = 330 nm, *λ*_em_ = 395 nm.

To check whether MPPS can probe the changes in the rigidity of the microenvironment of the protein during binding of 12-6-12,2Br^−^ with the BSA, the steady-state fluorescence anisotropy measurements of MPPS have been carried out. Depending on the changes in the microenvironment of MPPS in the protein during the unfolding process, the rigidity of the environment changes. It affects the free-tumbling motions of the probe. As fluorescence anisotropy is related to the flexibility of the microenvironment^102,103^ therefore, the fluorescence anisotropy values of MPPS in 5.0 µM of BSA have been estimated at different concentrations of 12-6-12,2Br^−^ and displayed in Fig. 11. As expected, the fluorescence anisotropy increases with increasing concentration of the surfactant in the specific binding region (region *a*) as the protein structure gets compacted and the tumbling motion of the probe gets restricted. In the competitive binding region (region *b*), the fluorescence anisotropy is decreased as the BSA unfolds, and the MPPS molecules experience a flexible environment due to the exposure to the bulk. As mentioned above, due to the rigid microdomain formation in the cooperative binding region (region *c*), the fluorescence intensity increases with a concomitant increase in the % α-helical content. These results are supported by the rise in the fluorescence anisotropy with increasing surfactant concentration in this region. Beyond this region, the fluorescence anisotropy decreases before reaching a saturation point due to an increase in the flexibility of the environment around MPPS as a consequence of the massive binding of the surfactants with the protein. Not many changes in the fluorescence anisotropy in the saturation binding region support that no further changes in the flexibility of the microenvironment occur in this concentration range of 12-6-12,2Br^−^. These results thus show that the MPPS is a potential molecule to probe the microrigidity of a biomolecule's environment.

#### 3.5.3. Fluorescence intensity decays of MPPS and lifetime measurements

The excited singlet state lifetime of the probe molecule calculated by the analysis of the fluorescence intensity decay measurements through a time-correlated single photon counting (TCSPC) method gives valuable information about the microenvironment and the changes in the microenvironment during a physical or chemical process.^81^ The intensity decays of MPPS in 10 mM HEPES buffer (pH = 7.4) in the absence and presence of 5.0 µM BSA are shown in Fig. S9(a).† The intensity decays of MPPS in 10 mM HEPES buffer (pH = 7.4) solutions of BSA (5.0 µM) in the presence of different concentrations of 12-6-12,2Br^−^ have been recorded. Some fluorescence intensity decays of MPPS at representative concentrations in non-cooperative, cooperative, and massive binding regions of binding isotherm of 12-6-12,2Br^−^ with BSA are displayed in Fig. S9(b)–(d),† respectively. All decays are biexponential, with a major contribution from the fast component in most cases. The lifetime values of the fast (*τ*_1_) and the slow (*τ*_2_) components, the pre-exponential factors of the fast and the slow components, *a*_1_ and *a*_2_, respectively (except for a few cases that are stated in the discussion), and the average lifetime (〈*τ*_f_〉) calculated using eqn S1 in Note S1† has been given in Table 5:

**Table 5:** Excited singlet state lifetime values of MPPS in different systems of BSA with increasing concentrations of 12-6-12,2Br^−^. [MPPS] = 5.0 µM, [BSA] = 5.0 µM, *λ*_ex_ = 330 nm, *λ*_em_ = 395 nm

[12-6-12,2Br^−^] (mM)	*a*_1_	*τ*_1_ (ps)	*a*_2_	*τ*_2_ (ps)	〈*τ*_f_〉 (ps)	*χ* ^2^
0.000	0.92 ± 0.04	166 ± 9	0.08 ± 0.03	1534 ± 37	281	1.17
0.008	0.92 ± 0.03	143 ± 22	0.08 ± 0.03	1558 ± 12	256	1.14
0.010	0.93 ± 0.04	119 ± 29	0.07 ± 0.02	1319 ± 53	207	1.03
0.030	0.96 ± 0.02	108 ± 34	0.04 ± 0.02	2105 ± 44	188	1.03
0.060	0.99 ± 0.03	96 ± 18	0.01 ± 0.02	1984 ± 36	112	1.03
0.090	0.44 ± 0.04	90 ± 17	0.56 ± 0.02	256 ± 23	182	1.01
0.110	0.44 ± 0.03	94 ± 18	0.56 ± 0.03	259 ± 32	187	1.02
0.130	0.48 ± 0.04	89 ± 14	0.52 ± 0.04	301 ± 41	199	1.05
0.150	0.95 ± 0.04	101 ± 36	0.05 ± 0.04	1270 ± 55	165	1.12
0.180	0.95 ± 0.03	96 ± 19	0.05 ± 0.03	1461 ± 37	164	1.05
0.200	0.95 ± 0.02	95 ± 24	0.05 ± 0.04	1451 ± 31	159	1.03
0.250	0.96 ± 0.03	95 ± 19	0.04 ± 0.04	1791 ± 27	163	1.04
0.300	0.96 ± 0.04	91 ± 27	0.04 ± 0.03	2319 ± 19	181	1.04
0.400	0.96 ± 0.04	94 ± 21	0.04 ± 0.03	2120 ± 28	175	1.01

The lifetimes of the fast and the slow components of MPPS in 10 mM HEPES buffer (pH = 7.4) are recorded from the intensity decay (Fig. S9(a)†) as 107 and 1299 ps with corresponding weightings as 96% and 4%, respectively, and an average lifetime as 155 ps (*χ*^2^ = 1.00). Theoretical calculations reveal the existence of two different MPPS conformers at the excited E-1 and E-2 states. As per the experimental results, the fast component appears to be a major contributor to the decay (except for a few cases described below). As discussed in detail below, we ascribe the fast and slow components to the conformers at E-1 and E-2 states, respectively. Also, the biexponential decay is noted in the presence of 5.0 µM of BSA in 10 mM of HEPES buffer solution (pH = 7.4) (Fig. S9(a)†). The lifetimes of the fast and the slow components are changed to 166 ps and 1534 ps with corresponding weightings of 92% and 8%, respectively, with an average lifetime of 281 ps. The docking study discussed below reveals only one MPPS location, *i.e.*, in the hydrophobic pocket of the BSA. Therefore, it is very unlikely that the two decay components are due to two different locations of MPPS in BSA. The average lifetime becomes longer when MPPS moves from the polar bulk to the hydrophobic pocket of BSA. Experimental solvatochromic data show that the fluorescence quantum yield and average lifetime are increased while the polarity of the environment is reduced. Theoretical results depict that coplanarity between the phenyl and the phenanthrene rings is enhanced for the E-1 conformer (majorly contributing to the decay as a fast component) with decreasing polarity of the MPPS environment, leading to decreased non-radiative deactivation of the excited state. As the rates of nonradiative processes are dropped in the non-polar environment (supported by lifetime data and radiative and nonradiative rate constant values in Table 1), the average lifetime of MPPS is longer in BSA than in the pure buffer. MPPS, being present in the hydrophobic pocket of BSA, feels a comparatively non-polar environment.

**Fig. 12:**
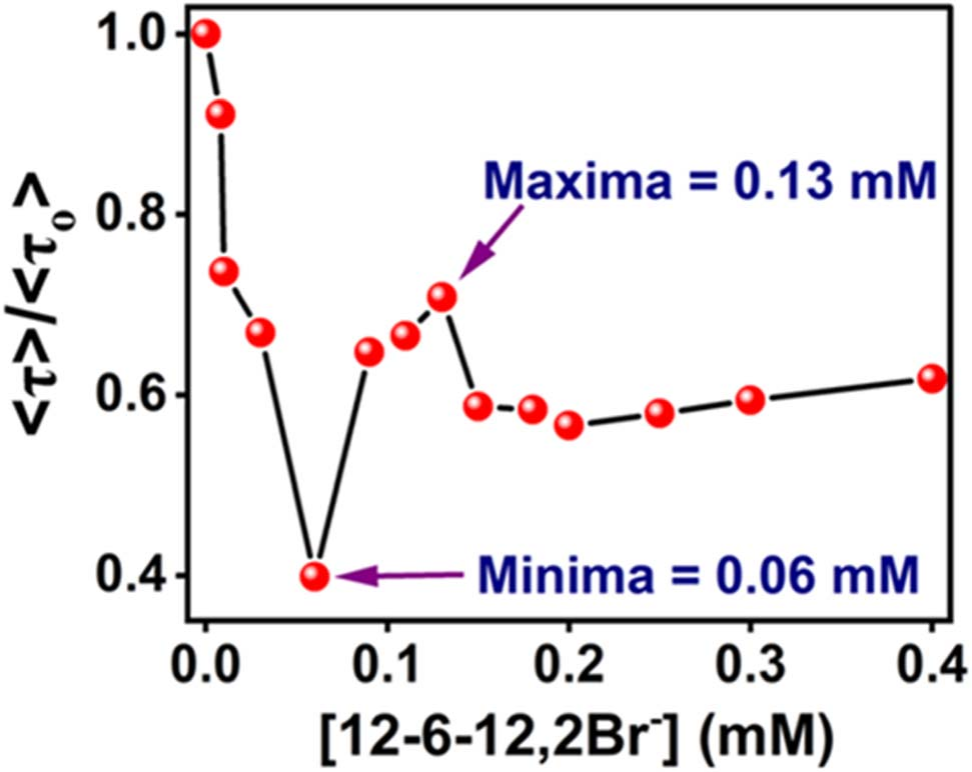
〈*τ*〉/〈*τ*_O_〉 plot of MPPS in the presence of BSA along with the increasing concentration of 12-6-12,2Br^−^. [MPPS] = 5 µM, [BSA] = 5.0 µM. (*λ*_ex_ = 330 nm, *λ*_em_ = 395 nm).

The ratios, 〈*τ*〉/〈*τ*〉_o_ (where, 〈*τ*〉 and 〈*τ*〉_o_ are the average lifetimes in the presence and absence of 12-6-12,2Br^−^, respectively) have been plotted in Fig. 12 shows that the variation in average lifetimes corroborates well with the steady-state fluorescence intensities (Fig. 9a) except for the specific binding region. We did not notice any increase in fluorescence lifetime as expected in the specific binding region; instead, a continuous decrease in lifetime occurred till 0.06 mM of the surfactant. This could be due to the less sensitivity to the microenvironment change in this low concentration range during specific binding. The average lifetime of MPPS is decreased when exposed to the polar bulk medium in the non-cooperative binding region due to the denaturation of the protein in the presence of surfactants. It can be seen from the data in Table 5 that, interestingly, in this binding region, the lifetime of the fast component is decreased with a concomitant increase in its weighting, and that of the slow component has an increasing tendency with a decrease in its weighting upon addition of surfactants up to 0.06 mM. Unless the coplanarity is reduced with increasing polarity of the medium, the fluorescence quantum yield and lifetime probably won't be decreased (Table 1). As per the results of the theoretical calculation, between the E-1 and E-2 states, the coplanarity in the E-1 conformer is reduced, and that of the E-2 conformer is increased with polarity. That is why the fast and slow components are ascribed to the E-1 and E-2 conformers, respectively. Therefore, it is obvious that the lifetime of the fast component (conformer in E-1 state) will be shorter, and that of the slow component (conformer in E-2 state) will be longer upon exposure of MPPS to the polar bulk environment in the non-cooperative binding region resulting in a decrease in an average lifetime (to be noted that the fast component is much more abundant than the slow component). As E-2 is more stable than E-1, there is a possibility that the E-2 conformer is undergoing the non-radiative processes due to the close proximity of the triplet state. That could be why E-1 has a more significant population than E-2, which contributes more to the decay.

In the cooperative binding region, micelles bind along the protein chain, forming a necklace-bead kind of structure.^19,20^ The average lifetime starts increasing with increasing surfactant concentration (Table 5 and Fig. 12). It indicates that the probe molecules, MPPS, are getting transferred to a comparatively non-polar environment, consistent with the steady-state fluorescence intensity changes (Fig. 9(a)) and lifetime data in solvents of different polarities (Table 1). A remarkable decrease in the weighting of the fast component from 99% to 44%, with a reduction in a lifetime from 96 to 90 ps for an increase in the concentration of the surfactant from 0.06 to 0.09 mM in the initial part of the cooperative binding region, is noted. Simultaneously, an increase in the weighting of the slow component from 1% to 56%, with a decrease in the lifetime from 1984 to 256 ps is observed. With a further increase in the concentration of the surfactant to 0.13 mM, the weighting of the fast component is increased to 48% with a decrease in lifetime to 89 ps, and the weighting of the slow component is decreased to 52% with an increase in the lifetime to 301 ps. The average lifetime of MPPS in the presence of BSA and 0.13 mM of the surfactant is 199 ps, which is shorter than that in the native BSA. The increase in the average lifetime above 0.06 mM of surfactant infers that the probe molecules, MPPS are moving to a comparatively non-polar environment. However, that microenvironment is less non-polar than the hydrophobic pocket of BSA. Notably, the average lifetime in native BSA is 281 ps. Also, the lifetime of the slow component is entirely different from that in the non-cooperative binding region of the BSA-surfactant system and even in homogeneous solvents. We suggest that the slow component is not due to the E-2 conformer. However, the fast component is still ascribed to the E-1 conformer, looking at the similarities between the lifetime values. The steady-state anisotropy (Fig. 11) and rotational relaxation time (discussed below) show that the binding region is more rigid than BSA. In this region, micelles are formed along the protein chain. Based on the results given here, it is proposed that MPPS molecules are located in the Stern layer (48%) and the micelles' core (which is relatively rigid) (52%). The E-1 state conformer of the MPPS being present in the Stern layer of micelle gives fluorescence with a lifetime of 89 ps. Another MPPS species located in the micelles' core emits fluorescence with a lifetime of 301 ps. The species present in the micelles' rigid core contribute to high steady-state anisotropy (Fig. 11) and slow rotational diffusion (discussed below). Of course, the hydrophobic microdomain created within the protein molecule leads to the compaction of the protein structure^80,82^ as supported by the increase in the % of α-helix (Table S6† and Fig. 10), which may also have an added role in the changes in fluorescence anisotropy. To support this fact, a control experiment on the lifetime of the MPPS in the micelles formed by 12-6-12,2Br^−^ at a concentration of fifteen times cmc has been conducted. For MPPS, *τ*_1_ = 92 ps, *a*_1_ = 0.46, *τ*_2_ = 329 ps, *a*_2_ = 0.54 (*χ*^2^ = 1.19); and, *τ*_average_ = 219 ps,. Excellent similarities between these values and that in the micelles (0.13 mM 12-6-12,2Br^−^) in the presence of BSA support that MPPS are present in the core, and the Stern layer of micelles. These micelles bind along the BSA chain. Interestingly, the fluorescence properties of MPPS nicely support the well-known necklace-beads types of BSA-surfactant complex in the cooperative binding isotherm region.^19,20^

In the massive binding region (beyond 0.13 mM and up to 0.4 mM in Table 5 and Fig. 12), the average lifetime is decreased because the probe molecules, MPPS, are once again exposed to the polar environment due to the open structure of the protein. Again, the fast component becomes significantly abundant with a decrease in lifetime, and the minor slow component's lifetime is increased as expected from the experimental solvatochromic data and theoretical results, resulting in a reduction of average lifetime before reaching saturation, as noted in steady-state fluorescence results.

It is noteworthy that the possibility of free rotation of the phenyl ring enables the molecule to alter the dihedral angle between the phenyl and phenanthrene rings by changing the environment's polarity. Also, the free rotation has a role in fluorescence intensity. To support this fact, we have synthesized another molecule, methyl(10-(*o*-tolyl)phenanthren-9-yl)sulfane (MTPS) (Scheme S1†), and the results of theoretical calculations performed on this molecule have been discussed above. It has been observed that only one conformer of MTPS exists in the excited state, and the coplanarity between the *o*-tolyl(substituted phenyl) and phenanthrene rings is lower than that in MPPS in all solvents. This is due to the presence of a –CH_3_ group in the *ortho* position of the benzene ring in MTPS; the free rotation of the benzene ring is restricted. The intensity decay of MTPS is monoexponential in 10 mM HEPES buffer (pH = 7.4) with a lifetime of 726 ps, which supports the existence of only one conformer of MTPS in the excited state. Also, this lifetime value is longer than the fast components of MPPS (107 ps). The average lifetime of MPPS is 155 ps, much shorter than the lifetime of MTPS (726 ps). Though the coplanarity of the *o*-tolyl and phenanthrene rings is lesser in MTPS, but due to the restricted rotation of the *o*-tolyl ring, the nonradiative deactivation is less in this case, resulting in a longer lifetime. This is further supported by the data in Fig. S6† that show that the fluorescence quantum yields of MTPS in all solvents are higher than that of MPPS. The lifetime values of MTPS calculated in the native protein, BSA, and the BSA in the presence of different concentrations of 12-6-12,2Br^−^ are given in Table S7.† The intensity decays are biexponential in these systems. The lifetimes of both the components and their corresponding weightings are pretty different and much longer than those of MPPS in native BSA and the non-cooperative binding isotherm region of 12-6-12,2Br^−^. These differences are more significant in the native BSA. The average lifetime values are significantly longer, which infers more restricted rotations in MTPS in all systems. The lifetime values are distinctly different from those in the simple HEPES buffer medium. The docking results predict one binding site of MTPS in BSA (discussed below). As per theoretical calculations, one species exists in the excited state in the gas phase and homogeneous solvents that support a monoexponential decay, as noted in the buffer medium mentioned above. Thus, based on all these results, the bi-exponential decay components in BSA and BSA-surfactant complexes are ascribed to two different conformational isomers that might exist in the excited states in these systems.

The data in Table S7† show that in the non-cooperative binding isotherm region, the lifetime of the fast component of MTPS is reduced with an increase in the weighting, and that of the slow component almost remains constant with a decrease in the weighting up to 0.05 mM concentration of the surfactant. At 0.06 mM of the surfactant, when the protein gets unfolded and exposed to a polar environment, the lifetimes of both fast and slow components are reduced drastically, with 96% and 4% weightings, respectively. The fast component's lifetime is 651 ps, which is close to that in pure buffer (726 ps). It indicates the fluorescence from the same conformer. However, a minor component with only 4% weighting could be due to some other conformer formed in the excited state. Beyond 0.06 mM surfactant concentration, in the cooperative binding region, the lifetimes of both components start increasing, reaching maximum values of the fast and slow components as 1378 and 3638 ps with 96 and 4% weightings, respectively, at 0.13 mM of surfactant. Like MPPS, we ascribe the major fast component to the conformer present in the Stern layer of the micelle and the minor slow component to another conformer that might be formed from the MTPS present in the core of micelles. To support this, the lifetime of MTPS has been measured in the presence of 12-6-12,2Br^−^ of concentration fifteen times its cmc. These values are as follows: *τ*_1_ = 1419 ps, *a*_1_ = 0.97, *τ*_2_ = 4012 ps, *a*_2_ = 0.03 (*χ*^2^ = 1.02); and, *τ*_average_ = 1497 ps. These are similar to those found in BSA with 0.13 mM of the surfactant, supporting our hypothesis of the presence of MTPS in the micelles of 12-6-12,2Br^−^ bind along the BSA chain. A very low % of MTPS in the micelles' core compared to MPPS could be due to its structural rigidity/size. Again, the decrease in the lifetime of MTPS in the massive binding region with a close lifetime value of the fast component (∼700–800 ps) as that in the pure buffer supports the denaturation of the protein. Thus like, MPPS, MTPS successfully probes the binding isotherm of 12-6-12,2Br^−^ with BSA, supporting necklace-bead model in the cooperating binding region.

#### 3.5.4. Fluorescence anisotropy decays and rotational relaxation parameters of MPPS

This study gives information about the rotational motions of the probe molecule and also the motions of the biomolecules themselves that are responsible for the depolarization.^5,11,103,104^ The rigidity/flexibility of the microenvironment around the probe molecule has a role in the rates of rotational diffusion processes and, therefore, the rotational relaxation time. In this work, the time-resolved fluorescence anisotropy, *r*(*t*) measurements of MPPS have been carried out on the same TCSPC instrument using polarizers, and *r*(*t*) values have been calculated using eqn S7 (ref. 81) in Note S1.† All anisotropy decays are found to be monoexponential and the anisotropy decay function is described by eqn S8 (ref. 81) in Note S1.†

**Fig. 13:**
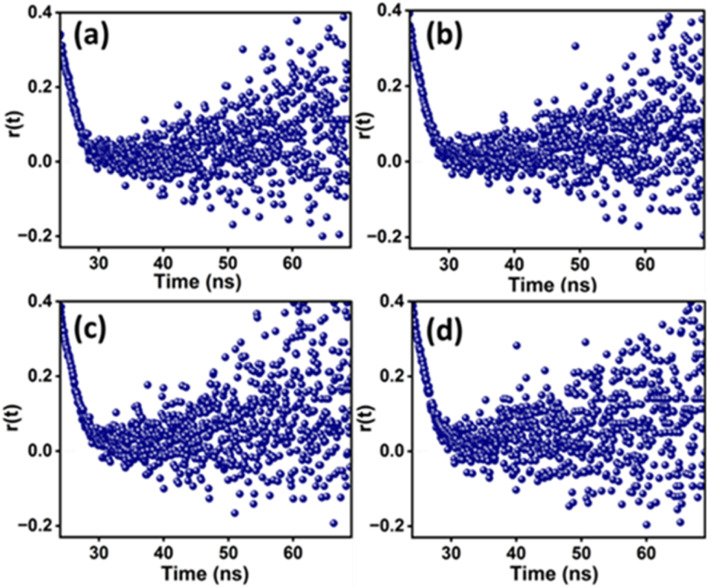
Fluorescence anisotropy decays of MPPS at (a) 0 mM (native BSA), (b) 0.06 mM (non-cooperative binding), (c) 0.13 mM (cooperative binding), and (d) 0.25 mM (massive binding) concentrations of surfactant, 12-6-12,2Br^−^. [MPPS] = 5.0 µM, [BSA] = 5.0 µM, *λ*_ex_ = 330 nm, *λ*_em_ = 395 nm.

**Fig. 14:**
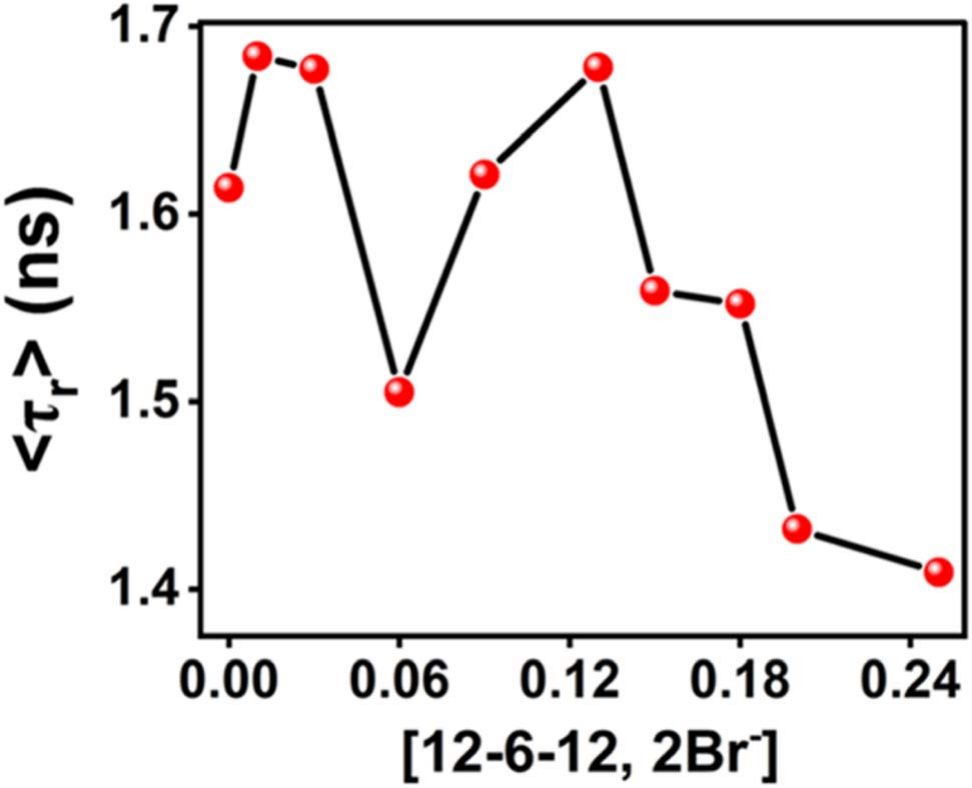
Rotational relaxation time (*τ*_r_) of MPPS in the presence of BSA with increasing concentration of 12-6-12,2Br^−^. [MPPS] = 5.0 µM, [BSA] = 5.0 µM. *λ*_ex_ = 340 nm, *λ*_em_ = 395 nm.

The anisotropy decays of MPPS in 5.0 µM of BSA in the presence of different concentrations of 12-6-12,2Br^−^ in 10 mM HEPES buffer solution have been recorded. The monoexponential decay supports only one kind of rotation and/or location of MPPS. The docking results show only one binding site of MPPS in BSA (discussed below). The anisotropy decays of MPPS at some representative concentrations in different regions of binding isotherm have been displayed in Fig. 13. The rotational relaxation parameters such as rotational relaxation times (*τ*_r_), corresponding relative amplitudes (*a*_r_), and limiting anisotropies (*r*_o_) (all explained in Note S1†) calculated based on recorded anisotropy decays are tabulated in Table 6. *τ*_r_ of MPPS in the presence of BSA with increasing concentration of 12-6-12,2Br^−^ are shown in Fig. 14. The data show that although the rotational relaxation times at all concentrations of the surfactant are quite long (∼1400 ps to ∼1680 ps), the limiting values of anisotropy (*r*_o_) are quite low and much away from the possible highest value of *r*_o_ = 0.40. These low values of *r*_o_ indicate that the decay component responsible for the depolarization should occur quite fast. It could be that we missed the fast component due to the limitation of our instrument. We ascribe this fast decay to the internal motions of MPPS.^103,104^ It is to be noted that this motion is mainly responsible for the depolarization of the MPPS molecules in the BSA, which can be stated based on the low value of *r*_o_. On the other hand, the comparatively longer rotational relaxation times that are tabulated in Table 6 could be due to the protein's flexible segmental motions around MPPS inside the hydrophobic pocket. The low values of *r*_o_ with expected major amplitudes for the fast decay components are in line with the assignment of S_0_–S_1_ transition as the *n*–π* transition with a change in transition dipole moments upon excitation. *τ*_r_ of MPPS in BSA at different concentrations of 12-6-12,2Br^−^ calculated after fitting the data to eqn S8 (ref. 81) in Note S1† are given in Table 6 and plotted in Fig. 14. As expected *τ*_r_ increases in the specific binding region due to the compaction of BSA, decreases in the non-cooperative region due to the unfolding of BSA, and once again increases in the cooperative region as a result of micelles formation leading to compaction of protein, and lastly decreases in the massive binding region for the open structure of the BSA.

**Table 6:** Rotational relaxation parameters obtained from the anisotropy decays of MPPS in different systems of BSA with increasing concentrations of 12-6-12,2Br^−^. [MPPS] = 5 µM, [BSA] = 5 µM, *λ*_ex_ = 330 nm, *λ*_em_ = 395 nm

[12-6-12,2Br^−^] (mM)	*α*_r_	*τ*_r_ (ps)	*χ* ^2^	*r* ^o^
0.00	1.00 ± 0.03	1614 ± 742	1.11	0.18
0.01	1.00 ± 0.04	1684 ± 762	0.98	0.16
0.03	1.00 ± 0.03	1677 ± 765	1.01	0.18
0.06	1.00 ± 0.03	1505 ± 683	0.95	0.18
0.09	1.00 ± 0.04	1621 ± 726	1.02	0.17
0.13	1.00 ± 0.02	1678 ± 764	1.09	0.18
0.15	1.00 ± 0.04	1550 ± 698	1.08	0.18
0.18	1.00 ± 0.04	1552 ± 691	0.99	0.18
0.20	1.00 ± 0.03	1432 ± 626	1.12	0.18
0.25	1.00 ± 0.04	1409 ± 606	1.05	0.15

### 3.6. Molecular docking study

**Fig. 15:**
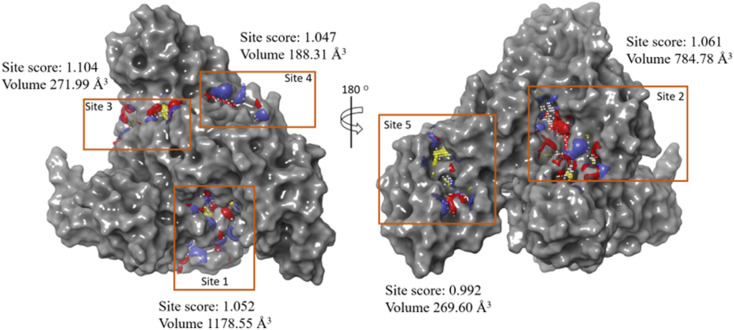
Sitemap analysis results show various predicted sites on BSA protein (PDB ID: 4F5S).

**Fig. 16:**
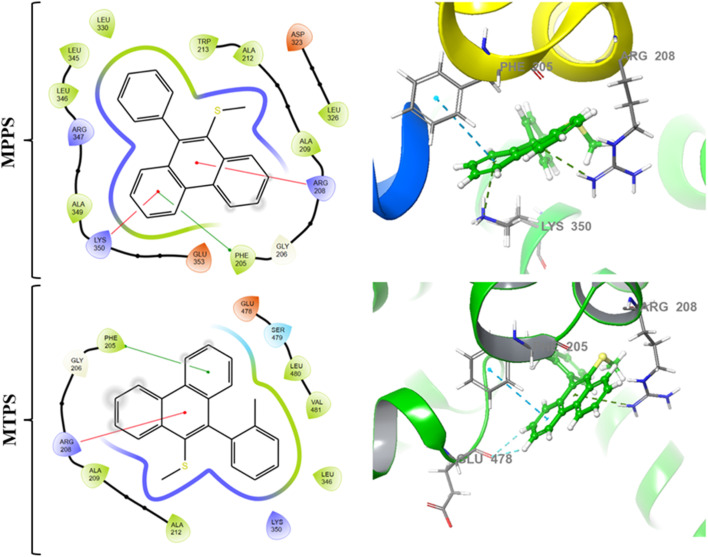
Molecular docking interactions [2D (left) and 3D (right)] exhibited by the test compounds MPPS and MTPS at the active site of the target protein (PDB ID: 4F5S).

In order to determine a plausible binding site on the BSA protein (PDB: 4F5S), site map analysis was carried out, which resulted in a total of five druggable sites (Fig. 15). Among these, the site with a large volume (site 1) is selected for generating a grid box, and the same is used for docking MPPS and MTPS molecules. From the docking results, as shown in Fig. 16, the glide score for MPPS was found to be −3.102 kcal mol^−1^ with a total of three interactions with the fused tricyclic system forming either π–π stacking or π–cation interaction. The three interactions include a π–π stacking interaction with Phe-205 with a bond length of 5.14 Å, and two π–cation interactions each with Arg-208 and Lys-350 with bond lengths of 3.8 Å and 3.29 Å, respectively. There were no H-bond interactions observed in our analysis. Notably, Trp-213 residue of BSA is found [Fig. 16 (2D representation)] nearby, whose fluorescence is quenched by MPPS, as discussed above. In the case of the MTPS molecule, the docking score was observed to be −2.438 kcal mol^−1^ with a total of four interactions, which include a π–π stacking with Phe-205 (5.07 Å), π–cation with Arg-208 (4.23 Å) and two with Glu4-78 *via* aromatic H-bonds (2.73 Å and 2.74 Å).

## 4. Conclusion

A molecule, methyl(10-phenylphenanthren-9-yl)sulfane (MPPS), with a simple chemical structure, has been synthesized, characterized and explored as a new fluorescent probe for microheterogeneous systems. Experimental and theoretical solvatochromic studies have been done to explore the photophysical properties of MPPS. As per theoretical calculations, the freely rotating phenyl ring forms a 94° dihedral angle with the phenanthrene ring in the ground state. Experimentally found two absorption bands are characterized as n → π^*^ and π → π^*^ transitions as supported by the frontier molecular orbital calculations. Theoretically, two excited state minima are ascribed to two conformers at the E-1 and E-2 states (the former being less stable than the latter in a polar medium). They exist in the gas phase with dihedral angles between the phenyl and phenanthrene rings as 142° and 133°, respectively. Conformers in these two states are also characterized as emitting species in a condensed medium, and their emissions are supported by the steady-state fluorescence and fluorescence intensity decay data. The dihedral angle between the phenyl and phenanthrene rings of the conformer of MPPS in the E-1 state decreases, and that of the conformer in the E-2 state increases with increasing polarity of the solvents. The changes in the dihedral angles with polarity affect emission energies, fluorescence quantum yield, excited singlet state lifetimes, and radiative and nonradiative processes. If the coplanarity between the phenyl and phenanthrene rings is enhanced, the fluorescence quantum yield and lifetime increase due to lesser rates of nonradiative and higher rates of radiative processes. Out of the two intensity decay components, the fast component contributes majorly. The fast major component is ascribed to the conformer in the E-1 state. To support that free rotation about the C–C bond has a role in fluorescence quantum yields and lifetimes, another molecule, MTPS, with comparatively restricted free rotation of the *o*-tolyl (substituted phenyl) ring, has been synthesized and characterized. The fluorescence quantum yields and lifetimes of MTPS are longer than MPPS due to a more restricted rotation of the benzene ring in the former than in the latter. The cmc of four different surfactants have been determined using MPPS as a polarity probe. All cmc values agree well with the literature reports. The fluorescence quenching of BSA by MPPS reveals the location of Trp residues of BSA. Different regions of binding isotherm (specific, non-cooperative, cooperative, and massive binding) of a gemini surfactant, 12-6-12,2Br^−^ with BSA have been successfully demonstrated by the steady-state and time-resolved fluorescence and fluorescence anisotropic properties of MPPS. Docking results show that MPPS resides in the hydrophobic pocket of BSA. Time-resolved fluorescence anisotropy results suggest an internal motion of MPPS and motions of the protein's flexible segment around MPPS in the hydrophobic pocket of BSA. Steady-state fluorescence anisotropy and rotational relaxation time of MPPS nicely change with changing the microenvironment's rigidity during the unfolding of the protein by the surfactant. So, MPPS has proved to be an excellent microrigidity probe as well. Thus, a polarity and molecular rigidity-sensitive fluorescent molecule, MPPS has been presented here that can potentially be used to monitor the changes in the microenvironment of biomolecules in different processes.

## Data availability

(1) Data for this article, including the synthetic methods for MPPS and MPTS probes are available at https://doi.org/10.1021/acs.joc.1c00861. (2) The data supporting this article have been included as a part of the ESI.† (3) No software or code has been included as a part of this paper.

## Author contributions

The manuscript was written through the contributions of all authors. All authors have approved the final version of the manuscript.

## Conflicts of interest

There are no conflicts of interest to declare.

## Supplementary Material

RA-014-D4RA05565A-s001
