# Supplementary material for: Presenting a new fluorescent probe, methyl(10-phenylphenanthren-9-yl)sulfane sensitive to the polarity and rigidity of the microenvironment: applications toward microheterogeneous systems
Source: RSC Adv. 2024 Aug 16;14(35):25865–88. doi: 10.1039/d4ra05565a (PMC11328280; doi:10.1039/d4ra05565a)
Supplement: RA-014-D4RA05565A-s001 [file RA-014-D4RA05565A-s001.pdf]

Supplementary Information (SI) for RSC Advances.
This journal is © The Royal Society of Chemistry 2024

Supplementary Information

Presenting a new fluorescent probe, methyl(10-phenylphenanthren-9-yl)sulfane sensitive to the polarity and rigidity of microenvironment: Applications toward microheterogeneous systems

Shalini Dyagala[a], Nilanjana Mukherjee[a,b#], Sayantan Halder[a,c#], Heena Charaya[d#], Mohammed Muzaffar-Ur-Rehman[e], Sankaranarayanan Murugesan[e], Shamik Chakraborty[d*], Tanmay Chatterjee[a*], Subit Kumar Saha[a*]

[a] *Department of Chemistry, Birla Institute of Technology & Science (BITS) Pilani, Hyderabad Campus, Hyderabad, Telangana 500078, India*

[b] *Present address: Department of Chemistry, Indian Institute of Technology Roorkee, Uttarakhand 247667, India*

[c] *Present address: Interdisciplinary Nanoscience Center (iNANO), Aarhus University, Gustav Wieds Vej 14,8000 Aarhus C, Denmark*

[d] *Department of Chemistry, Birla Institute of Technology & Science (BITS) Pilani, Pilani Campus, Rajasthan, 333031, India*

[e] *Department of Pharmacy, Birla Institute of Technology & Science (BITS) Pilani, Pilani Campus, Rajasthan, 333031, India*

[#]Authors with equal contribution

SECTION S1:

General Experimental Information. All reagents and solvents, including DMSO were purchased from BLD Pharma, TCI, and other local chemical companies. Flash column chromatography was performed using silica gel (100−200 mesh). The synthesized products were characterized by ^{1}H, ^{13}C, and ^{19}F NMR. NMR spectra were recorded on a Bruker 400 MHz instrument (400 MHz for ^{1}H NMR and 100 MHz for ^{13}C NMR). ^{1}H NMR experiments are reported in units, parts per million (ppm), and were measured relative to residual chloroform (7.26 ppm) in the deuterated solvent. ^{13}C NMR spectra are reported in ppm relative to deuterochloroform (77.00 ppm), and all were obtained with ^{1}H decoupling. Coupling constants are reported in hertz. Reactions were monitored by thin layer chromatography (TLC) and ^{1}H NMR of the crude reaction mixture using 1,3,5-trimethoxybenzene as the internal standard. Mass spectral data of unknown compounds were obtained on a high-resolution mass

spectrometer, HRMS (6546 Q-TOF LC/MS, Agilent). Melting points of unknown compounds were recorded on a KRUSS Optronic M3000 apparatus.

General Experimental Procedure for the Synthesis of MPPS and MTPS.

The designed 9-methylsulfenylphenanthrene derivatives *i.e.*, MPPS and MTPS were synthesized by following one[60] of our recently developed synthetic methods to access chalcogenyl phenanthrenes[61-63] using DMSO as the source of methylsulfenyl group (SMe). The synthetic route to MPPS and MTPS is presented in Scheme S1 given below. Both MPPS and MTPS were synthesized starting from the commercially available [1,1'-biphenyl]-2-amine **1** in three steps following literature protocols. At first **1** was converted to 2-iodo-1,1'-biphenyl **2** in 85% yield by *in situ* diazotization followed by the iodination of the diazonium salt (Step-1). Next, Pd-catalysed Sonogashira cross-coupling of **2** with two aryl alkynes, *i.e.*, phenylacetylene and 1-ethynyl-2-methylbenzene furnished 2-(phenylethynyl)-1,1'-biphenyl **3** in 85% and 2-(*o*-tolylethynyl)-1,1'-biphenyl **4** in 67% yield respectively. Finally, iodine-catalyzed methylsulfenylative annulation of **3** and **4** with DMSO in the presence of trifluoroacetic anhydride (3 equiv) at 120 °C furnished the desired products MPPS **5** in 88% and MTPS **6** in 63%, respectively.

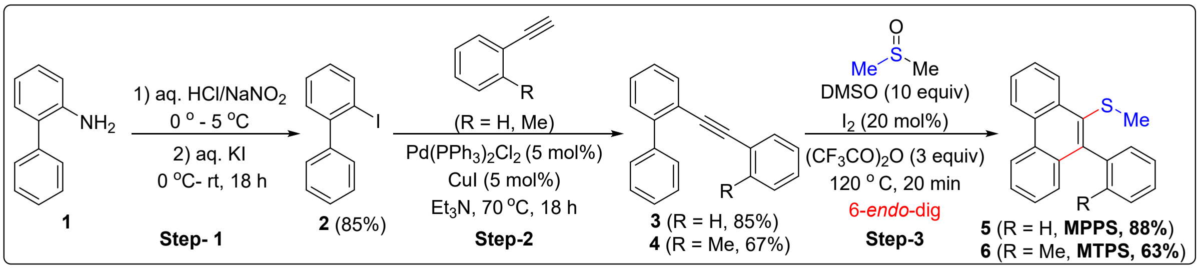

Scheme S1. Synthesis of **MPPS** and **MTPS** from commercially available [1,1'-biphenyl]-2-amine **1** in three steps.

The structures of MPPS[60] and MTPS were confirmed by ^{1}H and ^{13}C Nuclear Magnetic Resonance (NMR) (Figure S1) spectroscopy and High-Resolution Mass Spectrometry (HRMS).[37,60,64]

Experimental Procedure for the Synthesis of Methyl(10-(*o*-tolyl)phenanthren-9-yl)sulfane (MTPS) (6):

Step-1: [1,1'-biphenyl]-2-amine **1** (0.85 g, 5 mmol) was taken to aqueous HCl (2.6 mL, H_2O 8.3 mL) solution. The reaction mixture was cooled to 0-5 °C. After 5 min aqueous $NaNO_2$ solution (0.42 g in 8.3 mL H_2O) was added dropwise at 0-5 °C. The resulting solution turns

yellow after the addition of an aqueous KI solution (1.25 g in 8.3 mL H_2O). The reaction mixture was stirred vigorously for 18 h. After the completion of the reaction, the solution was quenched by the addition of aqueous $Na_2S_2O_3$ solution. The resulting solution was extracted by ethyl acetate (30 x 3 mL) and washed with water (30 mL). The combined organic layer was dried over anhydrous Na_2SO_4. The solvent was evaporated under reduced pressure to get the crude product which was purified by flash column chromatography on silica gel to afford 2-iodo-1,1'-biphenyl **2** (1.3 g, 4.3 mmol) in 85% yield.

The product was characterized fully by the ^{1}H and ^{13}C-NMR spectroscopy and the corresponding analytical data also matched with the reported literature.[37]

Step-2: In an RBF, $PdCl_2(PPh_3)_2$ (0.150 g, 0.22 mmol) and CuI (0.041 g, 0.22 mmol) were added to a solution of 2-iodo-1,1'-biphenyl (1.3 g, 4.3 mmol) in Et_3N (13 mL) under nitrogen atmosphere in a standard Schlenk-line process. The reaction mixture was stirred for 5 min. Then, 1-ethynyl-3-methylbenzene (566 µL, 5.16 mmol, 1.2 equiv) was added to the RBF. The resulting mixture was then heated under a nitrogen atmosphere at 70 °C for 18 h. The reaction mixture was allowed to cool to room temperature. Then, the solvent was evaporated under reduced pressure. The crude reaction mixture was extracted with ethyl acetate thrice (3 x 30 mL). The combined organic layer was washed with water three times (3 x 30 mL) and concentrated under reduced pressure to get the crude product which was purified by column chromatography through silica gel to afford 2-(*o*-tolylethynyl)-1,1'-biphenyl **4** (0.72 g, 2.7 mmol) in 67% yield.

The product was characterized fully by the ^{1}H and ^{13}C-NMR spectroscopy and the corresponding analytical data also matched with the reported literature.[64]

The other compound, 2-(phenylethynyl)-1,1'-biphenyl **3** was also synthesized by following the above-mentioned protocol and characterized fully by the ^{1}H and ^{13}C-NMR spectroscopy.[60]

Step-3: To a solution of 2-(o-tolylethynyl)-1,1'-biphenyl **4** (0.134 g, 0.5 mmol, 1 equiv) in DMSO **2a** (0.36 mL, 5 mmol, 1.4 M), I_2 (0.0254 g, 0.1 mmol) was added in a flame-dried RBF. Then, TFAA (0.212 mL, 1.5 mmol) was added to the RBF and the reaction mixture was stirred at 120 °C under an aerobic atmosphere. The progress of the reaction was monitored by TLC. After completion of the reaction, the resulting solution was extracted with ethyl acetate thrice (3 x 10 mL) and the combined organic layer was washed with water (3 x 10 mL). The solvent was evaporated under reduced pressure to afford the crude product which was purified by flash

column chromatography through silica gel to afford the product, methyl(10-(*o*-tolyl)phenanthren-9-yl)sulfane (**MTPS**) **6** (0.099 g, 0.31 mmol) in 63% yield.

The other product, methyl(10-phenylphenanthren-9-yl)sulfane[60] (MPPS) **5** was also synthesized by following the above-mentioned protocol and characterized fully by the ^{1}H and ^{13}C-NMR spectroscopy. [60]

Analytical Data

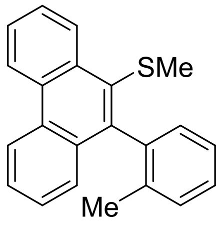

Methyl(10-(o-tolyl)phenanthren-9-yl)sulfane (MTPS, 6): White solid; mp = 82−84 °C; **^{1}H NMR (400 MHz, $CDCl_3$)** δ 8.87 – 8.85 (m, 1H), 8.81 – 8.79 (m, 1H), 8.76 (d, *J* = 8.3 Hz, 1H), 7.76 – 7.73 (m, 2H), 7.66 (td, *J* = 7.0, 3.5 Hz, 1H), 7.45 – 7.41 (m, 3H), 7.35 (dd, *J* = 7.0, 4.8 Hz, 1H), 7.31 (dd, *J* = 8.3, 0.9 Hz, 1H), 7.19 (d, *J* = 8.0 Hz, 1H), 2.23 (s, 3H), 2.01 (s, 3H); **^{13}C NMR (100 MHz, $CDCl_3$)** δ 144.53, 140.26, 136.60, 132.05, 131.58, 131.27, 130.85, 130.64, 129.83, 129.68, 127.75, 127.64, 127.59, 127.34, 127.19, 126.87, 126.79, 125.53, 123.02, 122.56, 19.93, 19.46. HRMS (ESI), m/z calcd for $C_{22}H_{19}S$ $[M + H]^{+}$: 315.1202; found: 315.1210.

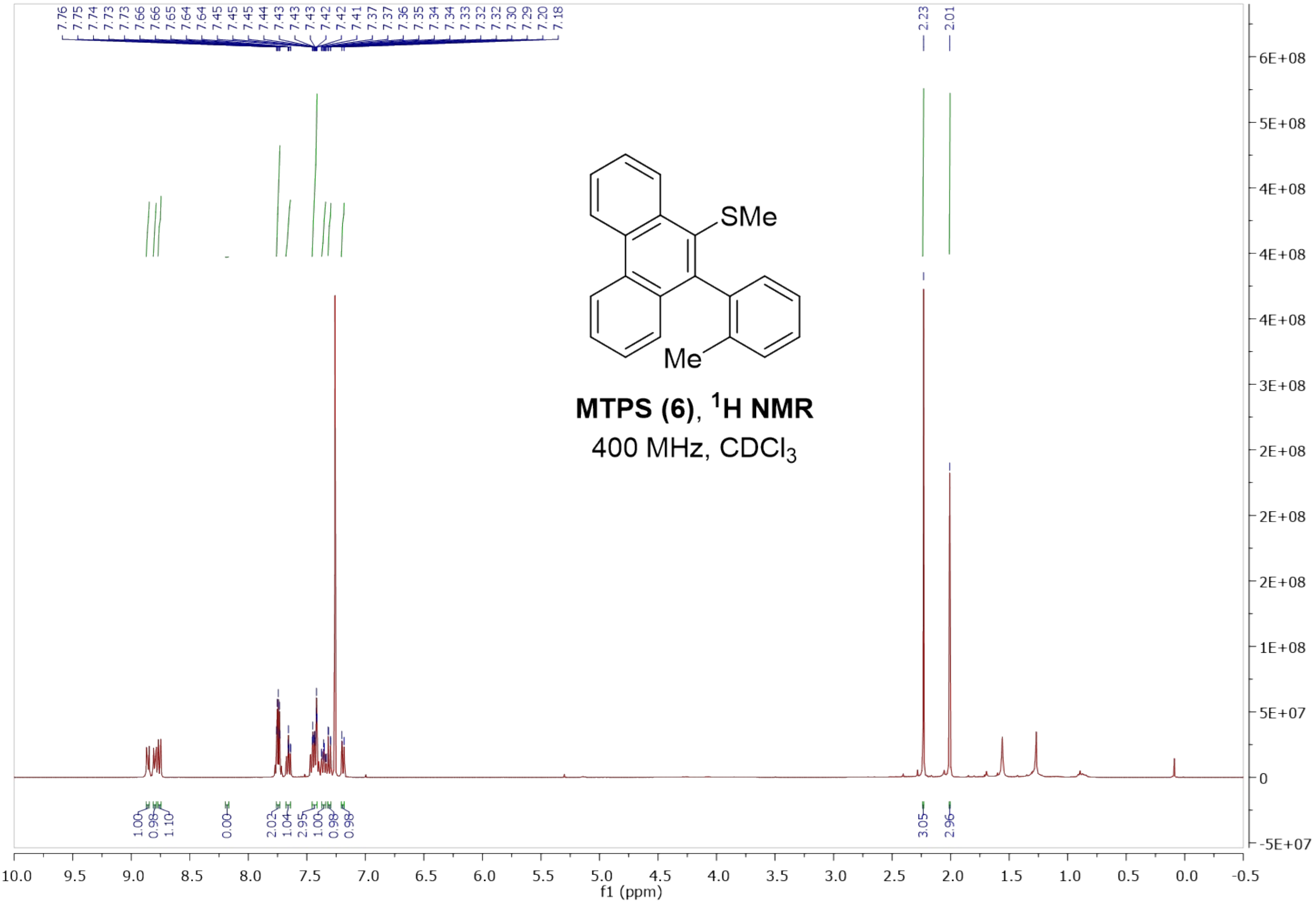

SMe
Me
MTPS (6), 1H NMR
400 MHz, CDCl3
f1 (ppm)

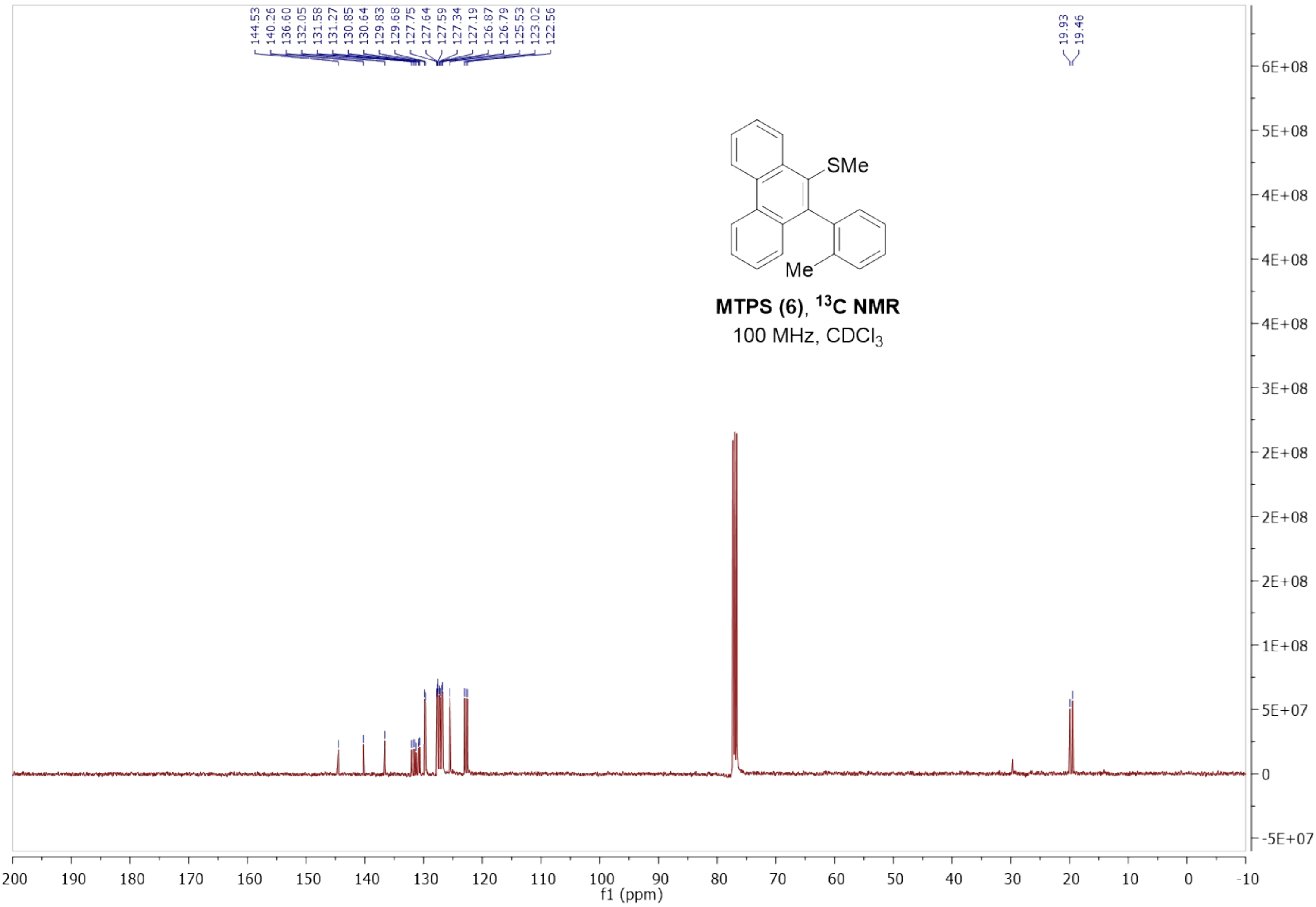

Figure S1: ^{1}H and ^{13}C NMR Spectra of MTPS (6)

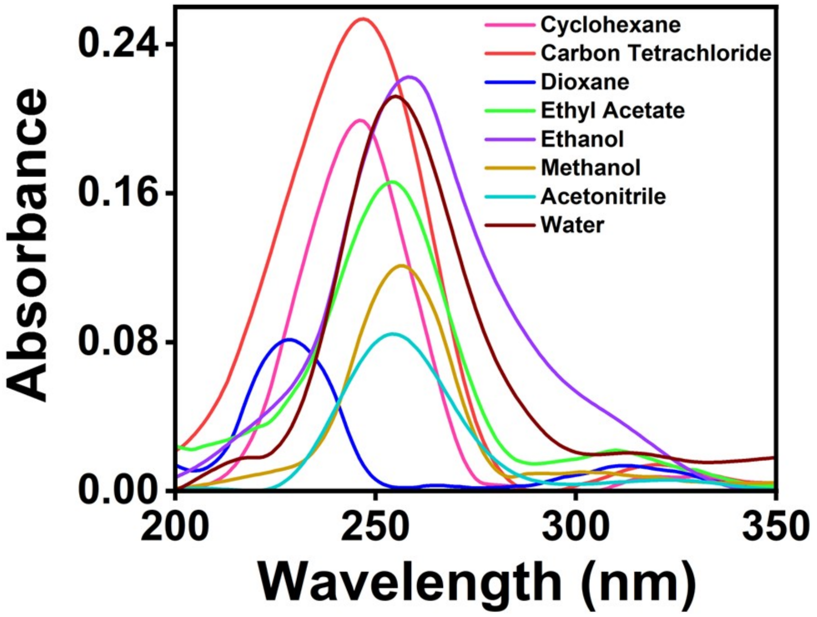

Figure S2: Absorption spectra of MPPS in different solvents. [MPPS] = 5.0 µM

Table S1. Energy difference of E-1 and E-2 in the gas phase and PCM, and relative energy of each of E-1 and E-2 of MPPS under solvation with respect to that in the gas phase at the CAM-B3LYP/6-311++g(d,p) level.

**Energy change ΔE (kJ mol^{-1})**	
E2(gas)-E1(gas)	4.0
E2(CCl_4)-E1(CCl_4)	-0.4
E2(MeOH)-E1(MeOH)	-5.2
E2(ACN)-E1(ACN)	-5.3
E2(H_2O)-E1(H_2O)	-5.3
E1(CCl_4)-E1(gas)	-9.0
E1(MeOH)-E1(gas)	-25.0
E1(ACN)-E1(gas)	-25.0
E1(H_2O)-E1(gas)	-26.0
E2(CCl_4)-E2(gas)	-13.0
E2(MeOH)-E2(gas)	-35.0
E2(ACN)-E2(gas)	-35.0
E2(H_2O)-E2(gas)	-36.0

Table S2. Dihedral angles for MPPS at the excited state (conformers at E-1 and E-2 states) in the gas phase and using PCM in MeOH solvent obtained using TD-DFT method at the CAM-B3LYP/6-311++g(d,p) level.

Medium	E-1		E-2	
	**Dihedral angle (∠C10-C1-C2-C5)**	**Dihedral angle (∠C8-C9-C10-C11)**	**Dihedral angle (∠C10-C1-C2-C5)**	**Dihedral angle (∠C8-C9-C10-C11)**
Gas phase	1°	3°	10°	9°
PCM (MeOH)	-4°	8°	10°	10°

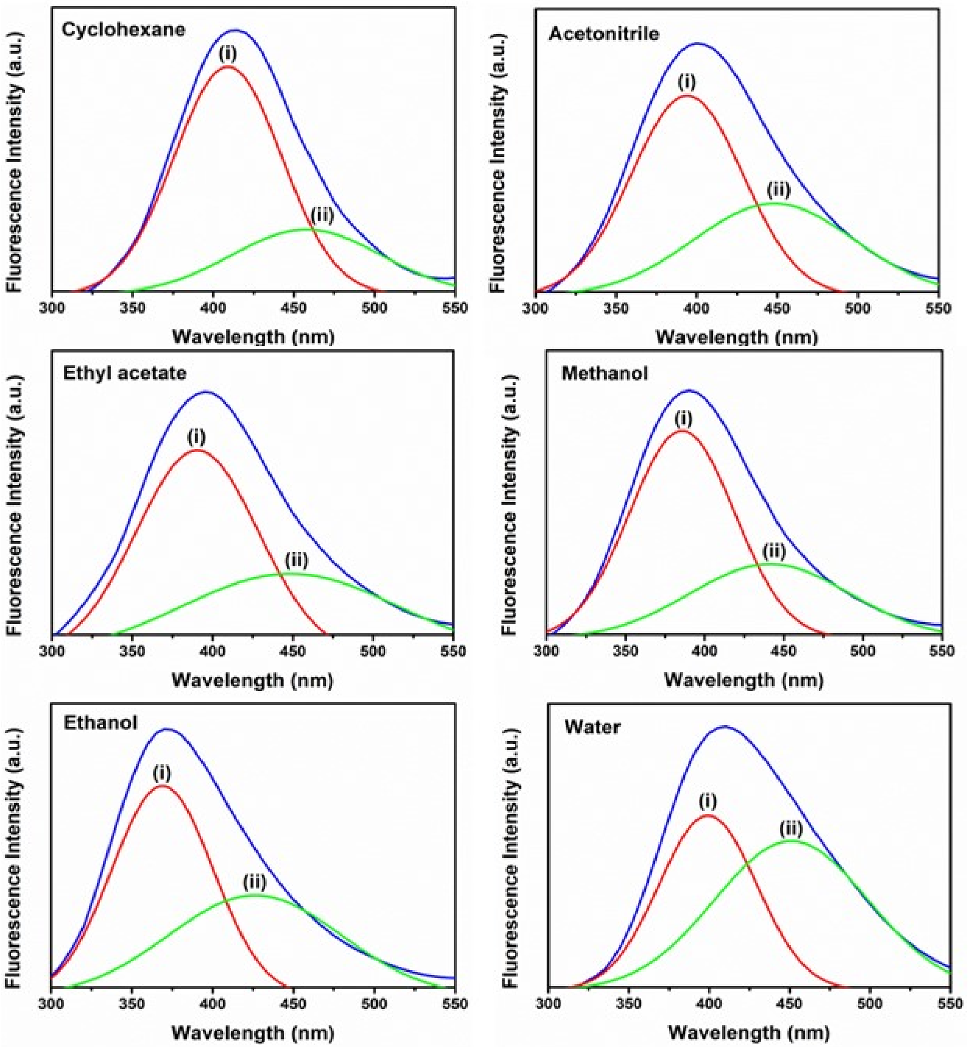

Figure S3: Deconvoluted fluorescence spectra of MPPS in solvents of different polarities. λ_{ex} for fluorescence measurements of MPPS in a particular solvent corresponds to a longer wavelength absorption peak maximum of MPPS (Given in Table 1).

Table S3: Fluorescence peak maxima of Deconvoluted bands of MPPS in solvents of different polarities.

Solvent	λ_{short} (nm)	λ_{long} (nm)
Cyclohexane	409	460
Ethyl acetate	390	448
Ethanol	369	426
Methanol	386	442
Acetonitrile	394	450
Water	399	451

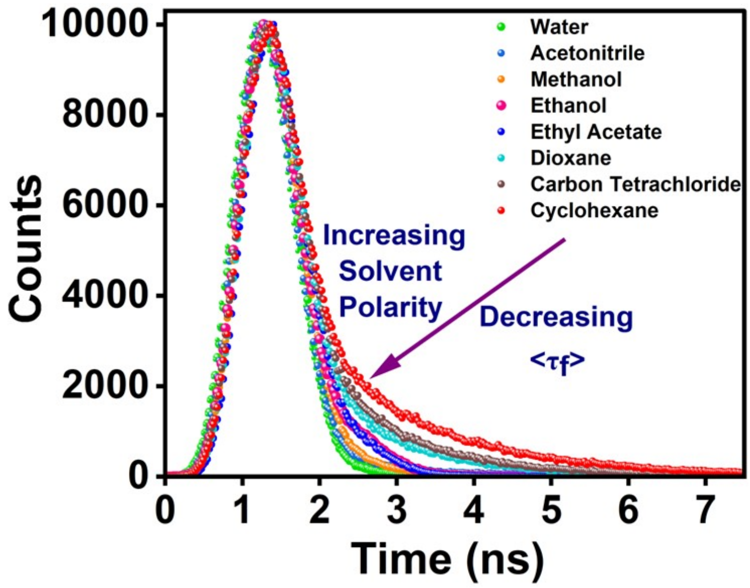

Figure S4: Fluorescence Intensity decays of MPPS in different solvents; λ_{ex} = 330 nm, and λ_{em} = 395 nm

Note S1:

Average lifetimes and radiative (k_r) and non-radiative (k_{nr}) rate constants of MPPS

The fluorescence intensity decays of MPPS in solvents of different polarities are bi-exponential in nature. The average lifetime values have been calculated using Equation S1[81]:

$$<\tau> \ = a_1\tau_1 + a_2\tau_2 \tag{S1}$$

where <τ> represents the average lifetime, a_1 and a_2 denote the pre-exponential factors of the fast and the slow components, respectively, while τ_1 and τ_2 signify the corresponding lifetimes of the fast and the slow components, respectively. k_r and k_{nr} are calculated using Equations S2[81]

and S3[81], respectively, using the values of fluorescence quantum yield (ϕ_f) and average lifetime (<τ>) given in Table 1, and are tabulated in the same.

$$k_r = \phi_f / <\tau> \quad \text{(S2)}$$

$$k_{nr} = (1 / <\tau>) - k_r \quad \text{(S3)}$$

Modified Stern-Volmer equation and the calculation of the fraction of Trp residues accessible to MPPS (f_a) and the Stern-Volmer constant (K_a)

To quantify the fractional accessibility of Trp residues to MPPS in native BSA, and to calculate the values of the fraction of Trp residues accessible to MPPS (f_a) and the Stern-Volmer constant (K_a) for the quenching of accessible fractions in the native protein, the modified Stern-Volmer plot based on Equation S4[81] has been used:

$$\frac{F_o}{(F_o - F)} = \frac{1}{f_a K_a [MPPS]} + \frac{1}{f_a} \quad \text{(S4)}$$

where F_o and F are the total fluorescence intensities of Trp residues in BSA in the absence and presence of MPPS at its concentration, [MPPS], respectively. K_a is the Stern-Volmer constant for the quenching of accessible fractions. f_a is the fraction of Trp residues accessible to MPPS. f_a can be written as (Equation S5[81]):

$$f_a = \frac{F_{0a}}{F_{0b} + F_{0a}} \quad \text{(S5)}$$

where the subscript 0 refers to the fluorescence intensity in the absence of the quencher. Thus, F_{0a} denotes the fluorescence intensity from the accessible fraction, while F_{0b} refers to the fluorescence intensity from the inaccessible fraction in BSA without the quencher.

Binding constant (K') of MPPS and the number of binding sites (n)

The binding constant (K') of MPPS and the number of binding sites (n) for both native and denatured BSA have been calculated from the linear fitting of the data in Figures S8a and S8b based on Equation S6 (given below), respectively. For a static quenching mechanism with the assumption that the protein has the same and independent binding sites, the following

equation (Equation S6) can be used to determine the binding constant (K') and the number of binding sites (n).[94]

$$\log[(F_o - F)/F] = n \log K' - n \log(1/([\text{MPPS}] - (F_o - F)[\text{P}]/F_o)) \qquad \text{(S6)}$$

where F_o and F are fluorescence intensities of BSA in the absence and presence of quencher at a concentration [MPPS], respectively, and [P] is the total concentration of the protein.

Fluorescence anisotropy decay, decay function, and rotational relaxation parameters

In this work, the time-resolved fluorescence anisotropy, $r(t)$ measurements of MPPS have been carried out on the same TCSPC instrument using polarizers, and $r(t)$ values have been calculated using Equation S7[81]:

$$r(t) = \frac{[I_{\parallel}(t) - GI_{\perp}(t)]}{[I_{\parallel}(t) + 2GI_{\perp}(t)]}$$

(S7)

where G indicates the correction factor corresponding to detector sensitivity to the emission's polarization detection, and $I_{\parallel}(t)$ and $I_{\perp}(t)$ represent fluorescence decays polarized parallelly and perpendicularly to the excitation light polarization, respectively. The G-factor is ~ 0.6 for our instrument. All anisotropy decays are found to be monoexponential, and the anisotropy decay function is described by Equation S8[81]:

$$r(t) = r_o a_r \exp(-t/\tau_r) \qquad \text{(S8)}$$

where, limiting anisotropy, r_o which deals with the inherent depolarization of the probe molecule, τ_r stands for rotational relaxation time for the rotational motion of the probe molecule, and a_r refers to a relative amplitude, where $a_r = 1$ for a mono exponential anisotropy decay.

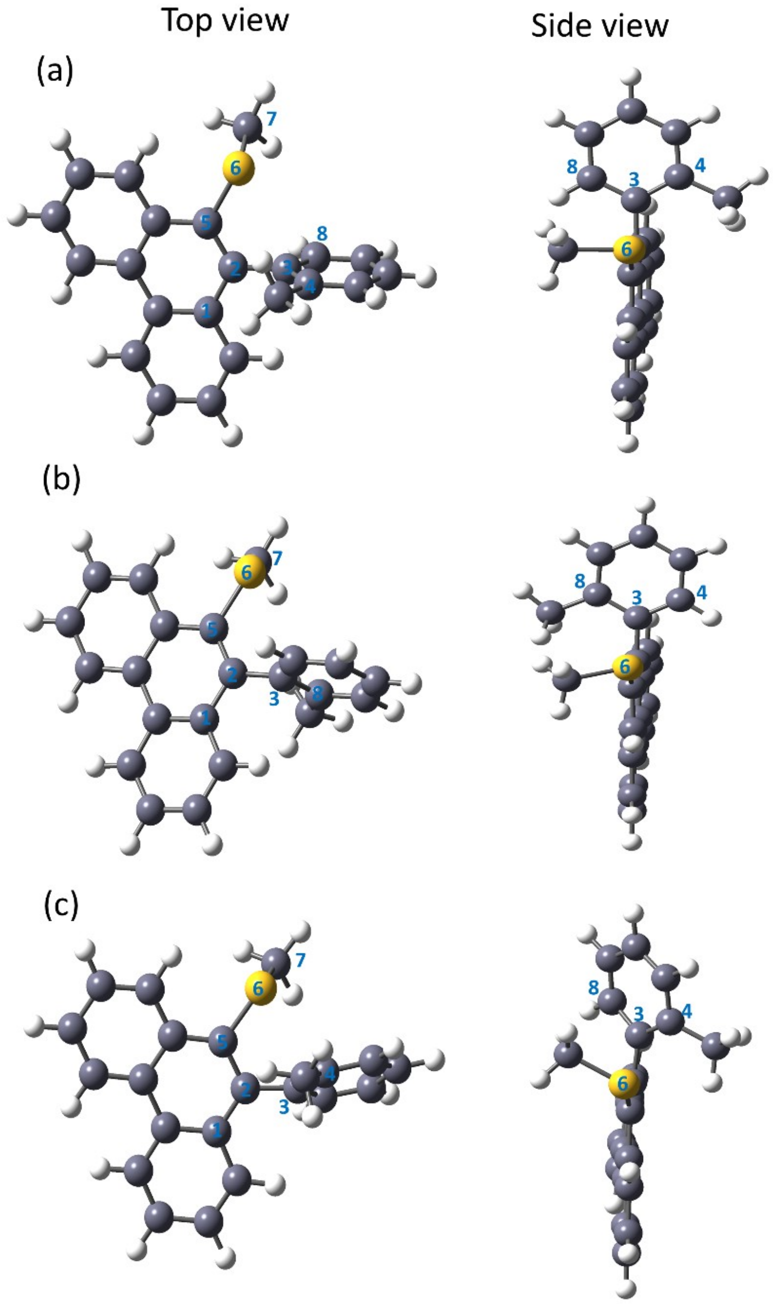

Figure S5. Optimized geometry of the molecule, MTPS : (a) ground state G-1, (b) ground state G-2, and (c) excited state (E-1) in the gas phase obtained at CAM-B3LYP/6-311++g(d,p) level of theory.

Table S4. Dihedral angles ∠C1-C2-C3-C4 (φ_1) and ∠C2-C5-C6-C7 (φ_2) of MTPS at the G-1, G-2, and E-1 in the gas phase and using PCM in CCl_4, MOH, ACN, and Water solvents obtained using CAM-B3LYP/6-311++g(d,p) level.

Medium		∠C1-C2-C3-C4 (φ_1)			∠C2-C5-C6-C7 (φ_2)		
		**G-1**	**G-2**	**E-1**	**G-1**	**G-2**	**E-1**
Gas		94°	97°	140°	96°	109°	62°
PCM	CCl_4	92°	97°	139°	99°	110°	62°
	ACN	91°	96°	74°	102°	111°	64°
	MeOH	91°	96°	74°	102°	111°	64°
	Water	90°	96°	74°	99°	111°	64°

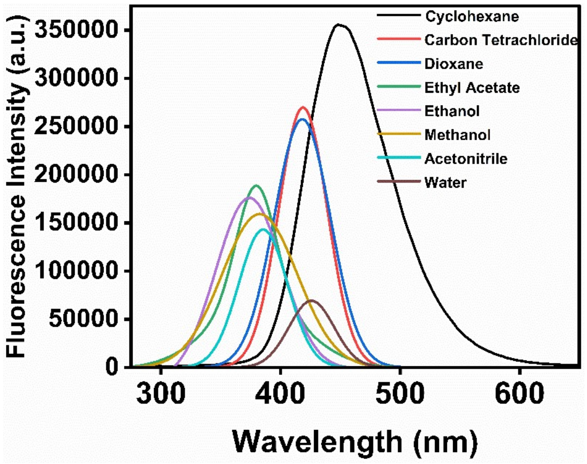

Solvents	ε	λ_{ab}^{max} (nm)	λ_{fl}^{max} (nm)	ϕ
Cyclohexane	2.02	258, 315	452	0.2256
Carbon Tetrachloride	2.24	251, 313	419	0.2103
Dioxane	2.25	257, 308	417	0.2036
Ethyl Acetate	6.02	256, 307	379	0.1816
Ethanol	24.55	258, 309	373	0.1656
Methanol	32.66	252, 309	381	0.1403
Acetonitrile	37.50	256, 306	385	0.1262
Water	78.36	255, 307	426	0.1002

Figure S6: Fluorescence spectra of MTPS in the solvents of different polarities. (Table) Absorption peak maxima, fluorescence peak maxima and fluorescence quantum yields of MTPS in the solvents of different polarities. λ_{ex} for steady-state fluorescence and quantum yield measurements of MTPS in a particular solvent corresponds to a longer wavelength absorption peak maximum of MTPS.

Table S5. Theoretical excitation energies for G-1 and G-2 conformer*s* and emission energies (E) of MTPS in the gas phase and using PCM in CCl_4, ACN, MOH, and water solvent obtained using the TD-DFT method at the CAM-B3LYP/6-311++g(d,p) level

Medium		Excitation (eV)		Emission (eV)
		G-1	G-2	E
Gas	S_1 State	4.26	4.25	3.82
PCM	CCl_4	4.26	4.25	3.82
	ACN	4.27	4.26	3.71
	MOH	4.27	4.26	3.71
	H_2O	4.27	4.26	3.71

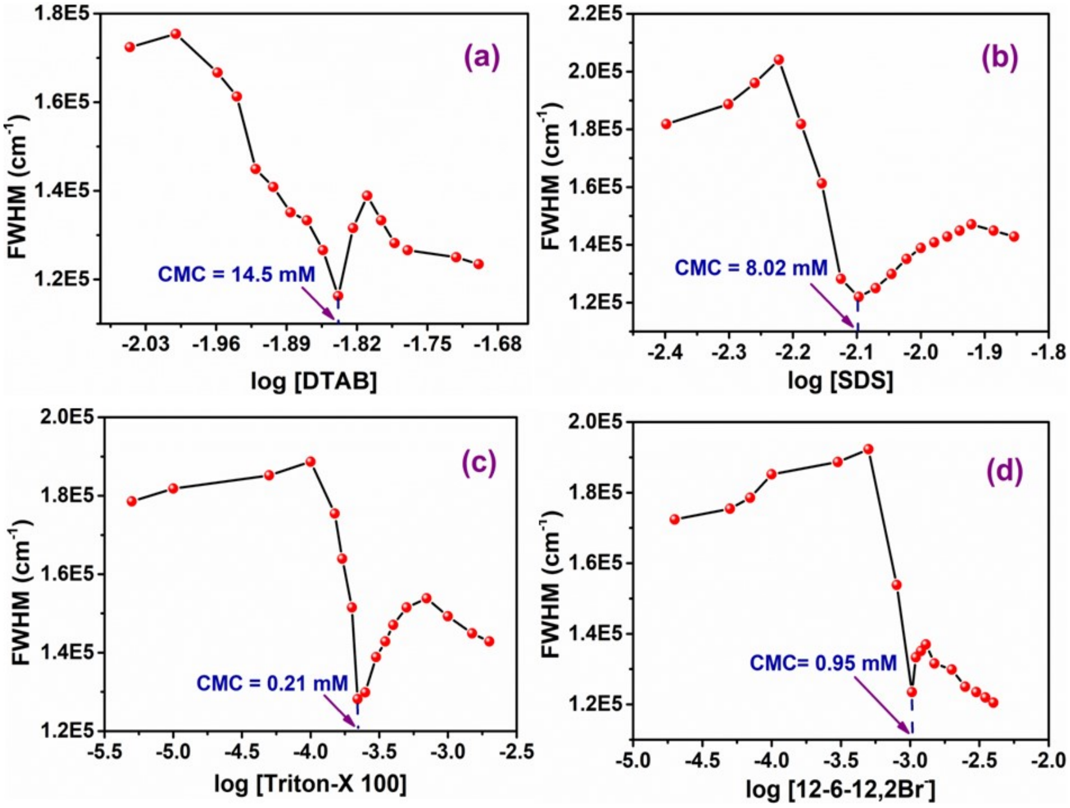

Figure S7: FWHM of fluorescence bands of MPPS versus concentrations of (a) DTAB, (b) SDS, (c) Triton-X 100, and (d) 12-6-12,2Br$^-$ surfactants. [MPPS] = 5 μM, λ_{ex} = 330 nm.

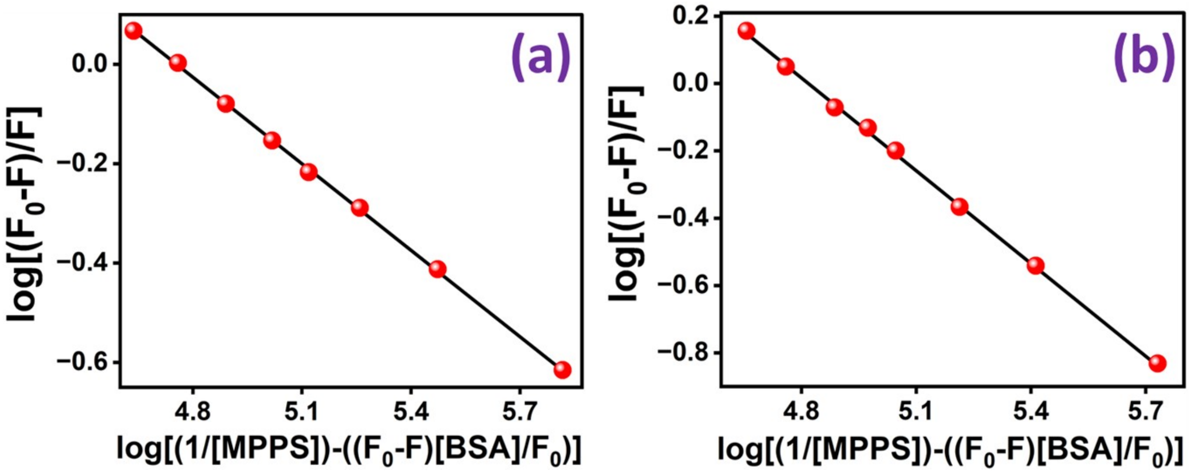

Figure S8: Plots of log[($F_o - F$)/F] versus log(1/([MPPS] – ($F_o - F$)[BSA]/F_o)) in the case of (a) native BSA, and (b) BSA + 0.3 mM 12-6-12,2Br$^-$. [BSA] = 5.0 μM. λ_{ex} = 295 nm. λ_{em} = 337 nm.

Table S6: % of various elements of secondary structures of BSA in the presence of different concentrations of 12-6-12,2Br$^-$.

[12-6-12] mM	*α*-Helix	*β*-Sheet		*β*-turn	Random coil
		Anti-parallel	Parallel		
0	60.7	3.5	4.7	12.6	21.1
0.008	62.3	3.6	4.9	10.7	16.4
0.01	64.8	2.2	3.4	13.6	23.7
0.03	21.5	4.5	5.1	14.1	23.4
0.06	3.36	11.3	11.6	18.9	40.6
0.09	16.81	7.6	8.3	16.4	32.7
0.11	34.4	4.2	5.1	13.1	22.2
0.13	49.4	3.3	4.4	12.1	20.5
0.15	44.8	3.4	4.7	12.3	23.4
0.18	43.1	3.6	6.9	11.7	48.1
0.2	47.8	3.1	6.3	11.9	49.3
0.25	46.5	4.2	7.4	12.3	50.4
0.3	42.9	4.9	8.2	14.5	55.2
0.4	44.4	5.6	8.4	14.9	58.4
0.5	44.2	6.3	9.2	15.6	60.9
0.7	43.9	7.9	10.9	16.9	65.3
0.9	42.4	9.6	14.6	17.2	68.2
1.00	39.9	16.1	40.4	19.6	79.6
5.00	31.8	16.7	42.6	17.8	88.4

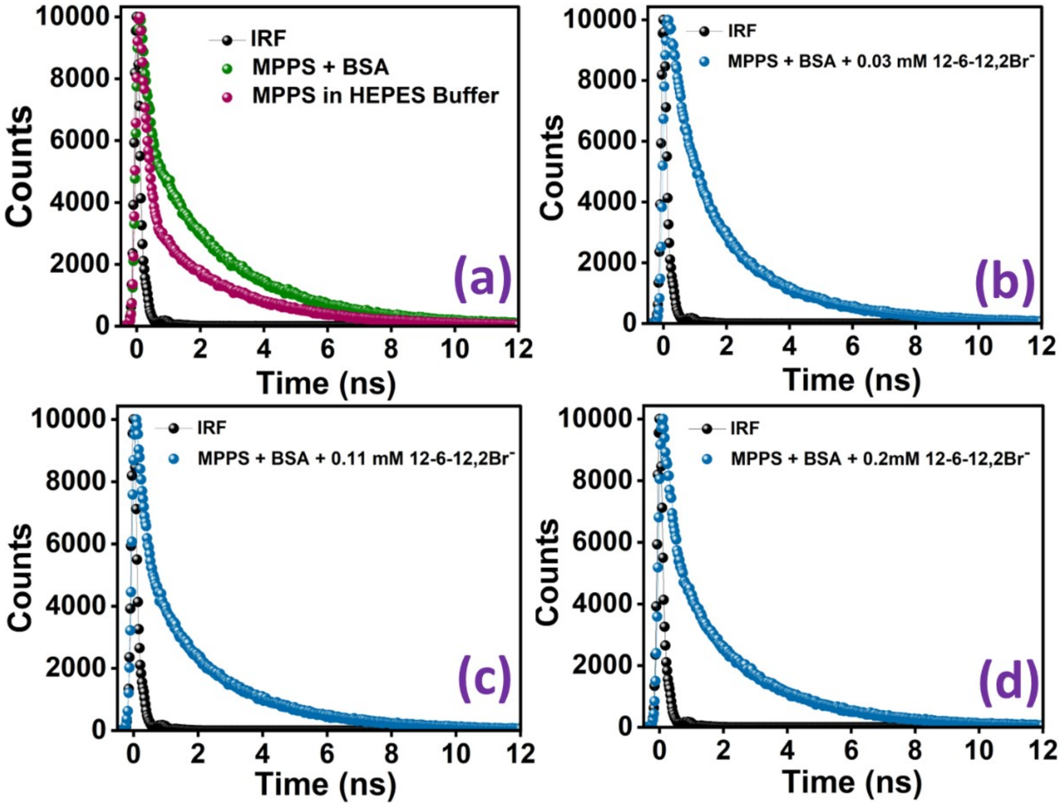

Figure S9: Fluorescence intensity decays of MPPS in (a) HEPES buffer, and HEPES buffer with 5.0 μM BSA, (b) HEPES buffer with BSA and 0.03 mM 12-6-12,2Br$^-$ (non-cooperative), (c) HEPES buffer with BSA and 0.11 mM 12-6-12,2Br$^-$ (cooperative), and (d) HEPES buffer with BSA and 0.2 mM 12-6-12,2Br$^-$ (massive binding). [BSA] = 5.0 μM, [HEPES buffer] = 10 mM, pH = 7.4; λ_{ex} = 330 nm, λ_{em} = 395 nm

Table S7: Excied singlet state lifetime values of MTPS in different systems of BSA with increasing concentrations of 12-6-12,2Br$^-$. [MTPS] = 5.0 μM, [BSA] = 5.0 μM, λ_{ex} = 330 nm, λ_{em} = 365 nm

[12-6-12,2Br$^-$] (mM)	a_1	τ_1 (ps)	a_2	τ_2 (ps)	<τ_f> (ps)	χ2
0.000	0.64±0.04	2562± 690	0.36± 0.02	6130± 291	3853	1.12
0.008	0.77± 0.03	2159 ± 289	0.23± 0.03	5989± 512	3035	1.05
0.010	0.85± 0.03	1953± 209	0.15± 0.04	6133± 232	2579	1.04
0.030	0.88± 0.03	2004± 216	0.12± 0.03	6086± 294	2481	1.16
0.050	0.85± 0.03	1909± 277	0.15± 0.04	5736± 288	2466	1.12
0.060	0.96± 0.02	651± 202	0.04± 0.04	1655± 247	691	1.06
0.011	0.97± 0.02	716± 263	0.03± 0.02	3854± 206	825	0.99
0.130	0.96± 0.03	1378± 634	0.04± 0.04	3638± 299	1467	0.98
0.150	0.96± 0.03	718± 512	0.04± 0.03	3846± 297	840	1.00
0.180	0.94± 0.02	791± 403	0.06± 0.04	3004± 201	920	1.09
0.200	0.94± 0.02	781± 317	0.06± 0.03	2927± 577	910	1.05
0.300	0.95± 0.03	765± 202	0.05± 0.04	3897± 211	915	1.19

Table S8: Cartesian coordinates of the optimized geometries of the MPPS and MPTS molecules obtained at the CAM-B3LYP level using 6-311++G(d,p) basis set.

	MPPS- G-1 (gas phase)		
Atom	X	Y	Z
C	0.099573	3.830417	0.172044
C	0.677235	2.591027	0.057065
C	-0.107892	1.420307	-0.005786
C	-1.514122	1.532887	0.044052
C	-2.077809	2.817885	0.166144
C	-1.293604	3.943383	0.229959
C	0.515178	0.116353	-0.11367
C	-2.3283	0.332279	-0.041558
C	-1.699703	-0.925299	-0.179373
C	-0.25073	-1.006152	-0.192071
C	-2.502681	-2.077453	-0.300039
H	-2.015263	-3.03236	-0.439474
C	-3.872415	-2.001487	-0.267442
C	-4.496355	-0.759163	-0.113328
C	-3.735955	0.378866	-0.00782
H	0.720791	4.716713	0.217359
H	1.753921	2.503566	0.012013
H	-3.151295	2.934894	0.208698
H	-1.756755	4.91849	0.322276
H	-4.46727	-2.901526	-0.366467
H	-5.57725	-0.691548	-0.084615
H	-4.241096	1.328144	0.100143
C	2.007306	0.045475	-0.117066
C	2.712626	0.033701	1.083222
C	2.715968	0.022941	-1.314824
C	4.099789	-0.02018	1.086801
H	2.16942	0.067798	2.020875
C	4.102504	-0.027507	-1.313057
H	2.174691	0.036748	-2.253616
C	4.798633	-0.051934	-0.112018
H	4.635423	-0.035322	2.02874
H	4.640656	-0.050929	-2.253285
H	5.881269	-0.093612	-0.11056
S	0.541463	-2.608296	-0.336897
C	0.391875	-3.19336	1.378759
H	-0.649772	-3.229418	1.69555
H	0.807562	-4.200589	1.40177
H	0.964855	-2.556229	2.050988

	MPPS- E-1 (gas phase)		
Atom	X	Y	Z
C	-0.735416	3.873937	-0.032086
C	0.090398	2.778974	-0.143093
C	-0.400799	1.458293	-0.038827
C	-1.807933	1.278111	0.054023
C	-2.61817	2.41242	0.184471
C	-2.102432	3.693067	0.163915
C	0.471716	0.313366	-0.104241
C	-2.393003	-0.068855	-0.043173
C	-1.541582	-1.201462	-0.122992
C	-0.122332	-0.993464	-0.098996
C	-2.131559	-2.482537	-0.194414
H	-1.496996	-3.358073	-0.237115
C	-3.497104	-2.648163	-0.216111
C	-4.333875	-1.535894	-0.156647
C	-3.776627	-0.274464	-0.070047
H	-0.320162	4.871799	-0.11019
H	1.140206	2.938807	-0.337554
H	-3.686876	2.297387	0.29633
H	-2.762452	4.545378	0.268837
H	-3.916337	-3.64547	-0.279342
H	-5.410078	-1.653983	-0.175332
H	-4.445521	0.573316	-0.025192
C	1.899174	0.383575	-0.257037
C	2.739729	1.251227	0.486371
C	2.553976	-0.587682	-1.082625
C	4.105079	1.102348	0.474747
H	2.294675	1.983299	1.146528
C	3.947865	-0.716448	-1.086219
H	1.984272	-1.065944	-1.868017
C	4.727769	0.104943	-0.305145
H	4.713206	1.7478	1.098081
H	4.409065	-1.44952	-1.738302
H	5.806098	0.007096	-0.30342
S	0.93118	-2.350733	0.096366
C	1.764071	-2.123682	1.704117
H	1.057478	-2.407884	2.484245
H	2.627327	-2.787604	1.714281
H	2.073543	-1.090337	1.828371

	E-2 - MPPS (gas phase)		
Atom	X	Y	Z
C	0.028119	3.882681	0.363304
C	0.687959	2.685693	0.221092
C	-0.009867	1.452549	0.125264
C	-1.440853	1.506142	0.05155
C	-2.074726	2.735299	0.24196

C	-1.365618	3.910473	0.414037
C	0.653352	0.196135	0.001715
C	-2.216457	0.305642	-0.303683
C	-1.567958	-0.958024	-0.288521
C	-0.185694	-0.98864	0.036075
C	-2.315703	-2.116866	-0.578497
H	-1.808615	-3.074317	-0.608872
C	-3.659678	-2.038989	-0.884944
C	-4.290755	-0.799942	-0.926033
C	-3.567071	0.352322	-0.645582
H	0.595624	4.802271	0.442623
H	1.765435	2.68602	0.168907
H	-3.155324	2.781752	0.246005
H	-1.892506	4.845049	0.562435
H	-4.213999	-2.940849	-1.116849
H	-5.339731	-0.72795	-1.185097
H	-4.078171	1.304196	-0.697954
C	2.086	0.016005	-0.234265
C	3.091884	0.67255	0.49823
C	2.500853	-0.894675	-1.226115
C	4.431638	0.445403	0.23522
H	2.818923	1.319816	1.321015
C	3.841229	-1.107607	-1.492588
H	1.746876	-1.413598	-1.806589
C	4.81962	-0.438987	-0.764983
H	5.183309	0.952098	0.829699
H	4.125914	-1.799392	-2.27698
H	5.869422	-0.61195	-0.96749
S	0.536825	-2.270172	0.952178
C	-0.690616	-2.715775	2.223958
H	-1.604193	-3.10668	1.784494
H	-0.221425	-3.473642	2.85074
H	-0.914701	-1.828406	2.815888

	G-1 - MPPS PCM (ACN)		
Atom	X	Y	Z
C	0.128246	3.830439	0.208671
C	0.698326	2.587251	0.087016
C	-0.094473	1.42209	0.006899
C	-1.5005	1.54446	0.04997
C	-2.057562	2.832686	0.177298
C	-1.265393	3.95279	0.255655
C	0.520923	0.114324	-0.105788
C	-2.322778	0.349291	-0.044813
C	-1.701867	-0.913099	-0.180589
C	-0.25268	-1.002836	-0.187844
C	-2.513804	-2.059225	-0.306306
H	-2.038111	-3.021155	-0.436582
C	-3.883957	-1.972776	-0.283448
C	-4.499744	-0.725061	-0.133407
C	-3.730982	0.407742	-0.020876
H	0.755015	4.711882	0.268572
H	1.774966	2.494502	0.052377
H	-3.130252	2.958132	0.213921
H	-1.722477	4.930157	0.352585
H	-4.484868	-2.868454	-0.384393
H	-5.580125	-0.649136	-0.111841
H	-4.230734	1.360078	0.085311
C	2.013007	0.037347	-0.113143
C	2.719342	-0.057933	1.083372
C	2.719306	0.093449	-1.312105
C	4.107104	-0.116263	1.080393
H	2.179345	-0.089847	2.022812
C	4.10689	0.037066	-1.316483
H	2.178505	0.175898	-2.247939
C	4.804819	-0.070215	-0.119961
H	4.643797	-0.196693	2.018346
H	4.643546	0.076549	-2.25707
H	5.887351	-0.114838	-0.12288
S	0.528703	-2.60965	-0.34256
C	0.310187	-3.257866	1.343631
H	-0.743767	-3.323355	1.608466
H	0.743042	-4.257683	1.348631
H	0.83906	-2.633622	2.0618

	E-1 - MPPS PCM (ACN)		
Atom	X	Y	Z
C	-0.461692	3.863713	0.10336
C	0.293864	2.722538	-0.034311
C	-0.282123	1.4235	0.04175
C	-1.710444	1.347179	0.088264
C	-2.442948	2.52058	0.257746
C	-1.839623	3.76957	0.292637
C	0.496921	0.231882	-0.001108
C	-2.384552	0.052352	-0.117568
C	-1.615558	-1.14535	-0.090158
C	-0.202599	-1.052957	0.090585
C	-2.301488	-2.379412	-0.21075
H	-1.742331	-3.303642	-0.1563
C	-3.665647	-2.432102	-0.402698
C	-4.406487	-1.255778	-0.473483
C	-3.75934	-0.035048	-0.326765
H	0.019209	4.833651	0.058251
H	1.353306	2.824112	-0.209691
H	-3.518909	2.471967	0.352703
H	-2.44157	4.659435	0.429123
H	-4.155592	-3.393605	-0.501613
H	-5.476928	-1.28564	-0.634368
H	-4.354731	0.866158	-0.377223
C	1.925741	0.225194	-0.240672
C	2.830084	1.066949	0.453447
C	2.489327	-0.702405	-1.154525
C	4.190104	0.977764	0.254128
H	2.453655	1.740474	1.211582
C	3.861958	-0.770562	-1.35968
H	1.83424	-1.303856	-1.768268
C	4.721992	0.060039	-0.660733
H	4.854477	1.611521	0.829988
H	4.255155	-1.474868	-2.083631
H	5.792313	0.00103	-0.815792
S	0.685501	-2.500675	0.416514
C	1.684685	-2.143929	1.898166
H	1.012941	-2.014917	2.746089
H	2.311765	-3.021176	2.048979
H	2.301945	-1.262161	1.760145

	E-2 - MPPS PCM (ACN)		
Atom	X	Y	Z
C	0.044949	3.915634	0.352172
C	0.701999	2.718435	0.191505
C	0.001764	1.479437	0.13079
C	-1.433514	1.539907	0.09085
C	-2.061973	2.764925	0.296863
C	-1.346532	3.944445	0.449199
C	0.650436	0.222187	0.024259
C	-2.213451	0.338808	-0.26757
C	-1.570841	-0.930706	-0.243518
C	-0.198562	-0.969797	0.110241
C	-2.321913	-2.082124	-0.566577
H	-1.826746	-3.043742	-0.601192
C	-3.660074	-1.991325	-0.897087
C	-4.284445	-0.74901	-0.930878
C	-3.555515	0.398843	-0.628321
H	0.614768	4.835439	0.410249
H	1.777347	2.724718	0.108056
H	-3.14234	2.813324	0.324841
H	-1.869212	4.87884	0.612239
H	-4.213932	-2.88761	-1.150418
H	-5.329462	-0.667048	-1.202908
H	-4.060514	1.35415	-0.679503
C	2.076207	0.026984	-0.214317
C	3.091186	0.720195	0.476931
C	2.487884	-0.929244	-1.168975
C	4.428881	0.487227	0.208901
H	2.828497	1.401934	1.274314
C	3.826896	-1.145601	-1.442389
H	1.735206	-1.479869	-1.720443
C	4.810986	-0.438694	-0.75782
H	5.183882	1.023135	0.772666
H	4.10579	-1.87086	-2.198074
H	5.85938	-0.614282	-0.965694
S	0.549764	-2.283163	0.948151
C	-0.734737	-3.045657	1.988236
H	-1.313095	-3.788209	1.446317
H	-0.194294	-3.528191	2.801451
H	-1.389281	-2.270336	2.382722

	G-1 - MPPS PCM (CCL4)

Atom	X	Y	Z
C	0.111568	3.830265	0.191285
C	0.686107	2.589268	0.073802
C	-0.102265	1.420955	0.002109
C	-1.508445	1.537739	0.048012
C	-2.069425	2.824086	0.1719
C	-1.281902	3.9473	0.242804
C	0.517642	0.115473	-0.108363
C	-2.32598	0.339466	-0.042347
C	-1.700519	-0.920023	-0.179579
C	-0.251434	-1.004811	-0.189121
C	-2.507302	-2.069396	-0.30434
H	-2.024605	-3.026968	-0.441366
C	-3.877255	-1.989008	-0.276854
C	-4.497876	-0.744542	-0.123605
C	-3.733913	0.391114	-0.01369
H	0.735184	4.714472	0.243704
H	1.762827	2.499467	0.034359
H	-3.142657	2.944513	0.210678
H	-1.742527	4.923435	0.336437
H	-4.474639	-2.887047	-0.378589
H	-5.578604	-0.67342	-0.098792
H	-4.236627	1.341763	0.093594
C	2.009812	0.042247	-0.114584
C	2.716573	-0.014591	1.083717
C	2.716247	0.063075	-1.31398
C	4.103957	-0.070059	1.082858
H	2.175452	-0.016307	2.023126
C	4.103213	0.010319	-1.316444
H	2.17409	0.113263	-2.251143
C	4.801197	-0.058997	-0.11781
H	4.640944	-0.120574	2.022783
H	4.639789	0.020772	-2.257826
H	5.883795	-0.102251	-0.119529
S	0.536521	-2.608427	-0.340663
C	0.357013	-3.223611	1.361867
H	-0.690073	-3.270402	1.657771
H	0.777658	-4.22879	1.374601
H	0.913592	-2.594705	2.054975

	E-1 - MPPS PCM (CCL4)		
Atom	X	Y	Z
C	-0.678323	3.875292	-0.010907
C	0.132679	2.768643	-0.119195
C	-0.377401	1.45288	-0.021022
C	-1.789685	1.295237	0.056899
C	-2.584184	2.439888	0.185932
C	-2.049496	3.714302	0.174852
C	0.477723	0.296699	-0.079721
C	-2.394871	-0.042543	-0.055153
C	-1.558386	-1.189052	-0.112562
C	-0.13791	-1.006763	-0.0635
C	-2.169403	-2.461937	-0.183447
H	-1.548472	-3.347954	-0.199876
C	-3.537262	-2.604932	-0.234827
C	-4.356868	-1.478555	-0.204373
C	-3.780101	-0.224577	-0.112334
H	-0.247529	4.867074	-0.082262
H	1.186123	2.915349	-0.302922
H	-3.655448	2.340255	0.28864
H	-2.697857	4.575665	0.27866
H	-3.971404	-3.595925	-0.297498
H	-5.434249	-1.578734	-0.247227
H	-4.435989	0.634208	-0.086991
C	1.90557	0.353762	-0.245717
C	2.754573	1.215364	0.496881
C	2.549541	-0.601948	-1.093331
C	4.120807	1.081569	0.452889
H	2.317328	1.930205	1.180784
C	3.942106	-0.715994	-1.131571
H	1.964169	-1.102604	-1.851958
C	4.733313	0.104978	-0.358717
H	4.736385	1.721579	1.07446
H	4.394916	-1.438662	-1.80074
H	5.81237	0.01784	-0.384756
S	0.885546	-2.386834	0.125825
C	1.745966	-2.162621	1.718377
H	1.045469	-2.423313	2.511908
H	2.592491	-2.847435	1.720769
H	2.082631	-1.13637	1.829103

	E-2 - MPPS PCM (CCL_4)		
Atom	X	Y	Z

C	0.027783	3.90173	0.356417
C	0.688833	2.706523	0.203443
C	-0.007288	1.468731	0.127599
C	-1.440665	1.52164	0.074144
C	-2.074619	2.747249	0.273876
C	-1.365022	3.926117	0.434317
C	0.651714	0.213695	0.011876
C	-2.215798	0.3196	-0.283193
C	-1.566393	-0.945049	-0.265469
C	-0.189912	-0.975971	0.076269
C	-2.311068	-2.101707	-0.577854
H	-1.804556	-3.058362	-0.616425
C	-3.652105	-2.020979	-0.896921
C	-4.284466	-0.782416	-0.929463
C	-3.562277	0.369343	-0.634438
H	0.59411	4.8231	0.422575
H	1.765048	2.711959	0.130746
H	-3.155286	2.791597	0.292571
H	-1.891806	4.859291	0.591413
H	-4.202856	-2.92074	-1.144932
H	-5.331653	-0.708404	-1.195283
H	-4.072978	1.321587	-0.684413
C	2.080331	0.027234	-0.225319
C	3.090595	0.706328	0.48354
C	2.494032	-0.911643	-1.193776
C	4.429095	0.474916	0.219539
H	2.822246	1.373997	1.2911
C	3.833674	-1.127161	-1.462466
H	1.740792	-1.449436	-1.757403
C	4.814537	-0.435378	-0.759517
H	5.182463	0.998803	0.796729
H	4.115669	-1.839885	-2.22888
H	5.863751	-0.610625	-0.963346
S	0.5498	-2.276913	0.945993
C	-0.709706	-2.888522	2.111371
H	-1.483626	-3.467988	1.61658
H	-0.172007	-3.519666	2.818122
H	-1.153771	-2.040297	2.63111

	G-1 - MPPS PCM (MeOH)		
Atom	X	Y	Z
C	0.128164	3.830419	0.20864
C	0.698263	2.587244	0.086991
C	-0.094516	1.422072	0.006892
C	-1.500542	1.544415	0.049977
C	-2.057618	2.832631	0.177302
C	-1.265472	3.952747	0.255638
C	0.520899	0.114318	-0.105802
C	-2.322797	0.349234	-0.0448
C	-1.701866	-0.913138	-0.180606
C	-0.252681	-1.002856	-0.187868
C	-2.513776	-2.059275	-0.30635
H	-2.038034	-3.021171	-0.436711
C	-3.883926	-1.972857	-0.283464
C	-4.499736	-0.725164	-0.133371
C	-3.730997	0.407649	-0.020833
H	0.754921	4.711873	0.26852
H	1.774902	2.4945	0.052333
H	-3.130312	2.958051	0.213924
H	-1.722572	4.930109	0.352558
H	-4.484822	-2.868543	-0.38445
H	-5.580119	-0.649263	-0.111789
H	-4.230759	1.359977	0.085377
C	2.012984	0.037359	-0.113158
C	2.71933	-0.057873	1.08335
C	2.719276	0.093439	-1.312119
C	4.107087	-0.116177	1.080368
H	2.179329	-0.089751	2.02279
C	4.106854	0.037092	-1.316501
H	2.178458	0.175826	-2.247946
C	4.804792	-0.070142	-0.119985
H	4.643789	-0.196578	2.018319
H	4.643504	0.076539	-2.257093
H	5.887324	-0.114756	-0.122911
S	0.52874	-2.609654	-0.342551
C	0.310478	-3.257585	1.343775
H	-0.743431	-3.322841	1.60886
H	0.743159	-4.257479	1.34884
H	0.839624	-2.633346	2.06175

	E-1 - MPPS PCM (MeOH)		
Atom	X	Y	Z
C	-0.462637	3.863876	0.102198

C	0.293077	2.722747	-0.035139
C	-0.28269	1.423717	0.041156
C	-1.710909	1.347087	0.087827
C	-2.443619	2.520489	0.256895
C	-1.840543	3.769563	0.29138
C	0.496772	0.232196	-0.001673
C	-2.384787	0.051995	-0.116968
Z	-1.61544	-1.145485	-0.089999
C	-0.202297	-1.052759	0.089147
C	-2.301162	-2.379772	-0.209453
H	-1.74169	-3.30381	-0.15491
C	-3.665416	-2.432943	-0.400271
C	-4.406672	-1.256835	-0.470838
C	-3.759776	-0.035946	-0.324963
H	0.01814	4.833866	0.056857
H	1.352509	2.824418	-0.210561
H	-3.519556	2.471754	0.351944
H	-2.442661	4.659365	0.427528
H	-4.155169	-3.394627	-0.498384
H	-5.477235	-1.287064	-0.630842
H	-4.355484	0.865055	-0.375187
C	1.925667	0.226022	-0.240343
C	2.829317	1.067743	0.454808
C	2.490263	-0.701353	-1.153896
C	4.189502	0.978483	0.257045
H	2.452016	1.741212	1.212563
C	3.863206	-0.769601	-1.357416
H	1.83586	-1.301951	-1.76918
C	4.72243	0.060748	-0.657331
H	4.85327	1.612098	0.833759
H	4.257184	-1.473703	-2.081141
H	5.792926	0.001733	-0.811181
S	0.687	-2.500384	0.412155
C	1.684507	-2.146078	1.895546
H	1.011885	-2.023749	2.743778
H	2.315633	-3.021024	2.042736
H	2.29761	-1.260789	1.761466

	E-2 - MPPS PCM (MeOH)		
Atom	X	Y	Z
C	0.041921	3.915639	0.352995
C	0.699908	2.719069	0.191261
C	0.000576	1.479626	0.130201
C	-1.434763	1.539012	0.090409
C	-2.064211	2.763373	0.29741
C	-1.349568	3.943297	0.450532
C	0.650104	0.222944	0.023581
C	-2.213728	0.337304	-0.268375
C	-1.570246	-0.931778	-0.242794
C	-0.198027	-0.969476	0.110775
C	-2.320245	-2.084025	-0.565082
H	-1.824229	-3.045271	-0.598055
C	-3.657998	-1.994316	-0.89748
C	-4.283096	-0.752355	-0.933515
C	-3.555349	0.396278	-0.630874
H	0.611031	4.835852	0.411381
H	1.775249	2.726088	0.107217
H	-3.14462	2.810823	0.325746
H	-1.872823	4.877191	0.614533
H	-4.211055	-2.891168	-1.150464
H	-5.327818	-0.671336	-1.206928
H	-4.060539	1.351385	-0.683394
C	2.075956	0.028402	-0.215321
C	3.090873	0.722052	0.47546
C	2.487475	-0.928063	-1.169865
C	4.428552	0.488641	0.207648
H	2.828104	1.404269	1.272402
C	3.826429	-1.144835	-1.443044
H	1.734739	-1.47874	-1.721166
C	4.810527	-0.437947	-0.758505
H	5.183682	1.024623	0.771145
H	4.105296	-1.870484	-2.198367
H	5.858894	-0.614052	-0.966037
S	0.551714	-2.281801	0.949152
C	-0.731354	-3.042083	1.992764
H	-1.311756	-3.784727	1.453121
H	-0.189622	-3.524366	2.805271
H	-1.384365	-2.265829	2.388077

	G-1 - MPPS PCM (Water)

Atom	X	Y	Z
C	0.128716	3.830565	0.208771
C	0.698692	2.587304	0.087092
C	-0.094218	1.422202	0.006911
C	-1.500253	1.544723	0.049917
C	-2.057239	2.833008	0.177256
C	-1.264937	3.953047	0.255698
C	0.521061	0.114362	-0.105721
C	-2.322668	0.349621	-0.044887
C	-1.701871	-0.912873	-0.180483
C	-0.252673	-1.002717	-0.187703
C	-2.513959	-2.058935	-0.306031
H	-2.038554	-3.02107	-0.435799
C	-3.884129	-1.97231	-0.283333
C	-4.499786	-0.724472	-0.133594
C	-3.730891	0.408283	-0.021116
H	0.755551	4.711948	0.268774
H	1.775332	2.494528	0.052547
H	-3.129905	2.958599	0.213892
H	-1.721931	4.930442	0.352688
H	-4.485131	-2.867948	-0.384026
H	-5.580152	-0.648412	-0.112116
H	-4.230586	1.360663	0.084932
C	2.013141	0.037282	-0.113059
C	2.719414	-0.058072	1.083517
C	2.719487	0.093302	-1.312036
C	4.107201	-0.116562	1.080571
H	2.179441	-0.090027	2.022963
C	4.107101	0.036703	-1.316379
H	2.178793	0.175957	-2.247921
C	4.804982	-0.070652	-0.119797
H	4.643841	-0.196999	2.018549
H	4.643801	0.076234	-2.256938
H	5.88751	-0.115332	-0.122665
S	0.528491	-2.609626	-0.342574
C	0.308463	-3.259496	1.342828
H	-0.745752	-3.326397	1.606159
H	0.742387	-4.25884	1.347471
H	0.835684	-2.635193	2.062137

	E-1 - MPPS PCM (Water)		
Atom	X	Y	Z
C	-0.457339	3.862758	0.112448
C	0.297822	2.721738	-0.027857
C	-0.278891	1.42235	0.046048
C	-1.707875	1.347359	0.090916
C	-2.439653	2.520262	0.263975
C	-1.835341	3.768994	0.302346
C	0.497758	0.230299	0.003302
C	-2.382698	0.054032	-0.122612
C	-1.615717	-1.14485	-0.089785
C	-0.20438	-1.0539	0.102113
C	-2.30232	-2.37789	-0.216639
H	-1.744907	-3.303052	-0.159916
C	-3.665365	-2.428182	-0.418656
C	-4.403598	-1.250725	-0.494879
C	-3.755592	-0.030633	-0.343005
H	0.024	4.832572	0.06958
H	1.357299	2.823472	-0.202639
H	-3.515707	2.471755	0.358548
H	-2.436595	4.658873	0.441693
H	-4.156093	-3.388801	-0.522093
H	-5.472762	-1.278814	-0.664343
H	-4.348857	0.871753	-0.398076
C	1.925884	0.220077	-0.243414
C	2.835465	1.062713	0.442176
C	2.481863	-0.710241	-1.158414
C	4.194123	0.973841	0.231394
H	2.465497	1.737162	1.202612
C	3.852274	-0.778038	-1.375553
H	1.821876	-1.317944	-1.760744
C	4.718221	0.055122	-0.686149
H	4.862992	1.609078	0.800363
H	4.239674	-1.484575	-2.100449
H	5.787177	-0.003991	-0.850351
S	0.676464	-2.501503	0.448085
C	1.687327	-2.126178	1.917101
H	1.022046	-1.950936	2.761708
H	2.287663	-3.017142	2.094287
H	2.331465	-1.268812	1.751307

	E-2 - MPPS PCM (Water)		
Atom	X	Y	Z
C	0.042681	3.916287	0.352709
C	0.70052	2.719639	0.190506
C	0.001014	1.480079	0.130417
C	-1.434459	1.539829	0.091379
C	-2.063653	2.764118	0.298806
C	-1.348721	3.944115	0.451444
C	0.649989	0.223301	0.02438
C	-2.213609	0.338154	-0.267581
C	-1.570487	-0.931176	-0.241647
C	-0.198488	-0.969277	0.112758
C	-2.320711	-2.083119	-0.564708
H	-1.82531	-3.044637	-0.59735
C	-3.65827	-1.992883	-0.897977
C	-4.282948	-0.750738	-0.934115
C	-3.554932	0.39769	-0.630849
H	0.61194	4.83644	0.410622
H	1.775817	2.726779	0.105865
H	-3.144044	2.811732	0.327723
H	-1.871775	4.878043	0.615861
H	-4.211422	-2.889566	-1.151315
H	-5.327514	-0.669292	-1.207989
H	-4.059827	1.352957	-0.683412
C	2.075735	0.028345	-0.214796
C	3.090952	0.722682	0.47498
C	2.487019	-0.928861	-1.168818
C	4.428587	0.489226	0.206766
H	2.828576	1.405648	1.271405
C	3.825914	-1.145678	-1.442403
H	1.734256	-1.480173	-1.719436
C	4.810262	-0.438095	-0.758842
H	5.183859	1.025833	0.769465
H	4.104542	-1.871896	-2.197265
H	5.858556	-0.614227	-0.966717
S	0.551631	-2.281854	0.950043
C	-0.73223	-3.049633	1.986996
H	-1.303534	-3.797421	1.444746
H	-0.191843	-3.526811	2.803372
H	-1.393378	-2.277789	2.3773

	G-1 - MPTS (gas phase)		
Atom	X	Y	Z
C	0.037867	3.824279	-0.282307
C	-0.550429	2.587249	-0.200069
C	0.225408	1.412044	-0.110554
C	1.633202	1.516822	-0.104249
C	2.208052	2.799619	-0.194306
C	1.432916	3.929839	-0.281382
C	-0.409864	0.112474	-0.022046
C	2.436931	0.310767	0.000362
C	1.79625	-0.943428	0.114001
C	0.347172	-1.012551	0.091952
C	2.587965	-2.102859	0.239895
H	2.090209	-3.055917	0.352445
C	3.958623	-2.036274	0.239787
C	4.594795	-0.796817	0.11555
C	3.845233	0.347675	0.002098
H	-0.576187	4.71427	-0.348259
H	-1.628626	2.503943	-0.204021
H	3.282983	2.911123	-0.192029
H	1.90458	4.903	-0.347568
H	4.544748	-2.941907	0.339944
H	5.676526	-0.736821	0.113341
H	4.359518	1.294003	-0.087119
C	-1.902731	0.049991	-0.087764
C	-2.520488	0.037877	-1.335109
C	-2.683351	0.045595	1.07483
C	-3.90272	0.001417	-1.449009
H	-1.905063	0.063954	-2.227578
C	-4.068889	0.013556	0.94396
C	-4.680311	-0.011667	-0.301512
H	-4.366889	-0.011283	-2.427901
H	-4.680157	0.006917	1.839846
H	-5.76124	-0.037065	-0.373156
S	-0.461827	-2.604718	0.256145
C	-0.435429	-3.165955	-1.473007
H	0.583223	-3.20097	-1.857525
H	-0.855451	-4.171602	-1.481437
H	-1.050211	-2.517306	-2.095122
C	-2.048164	0.049661	2.438202
H	-1.550797	-0.9034	2.637133
H	-1.293123	0.833253	2.529308
H	-2.797022	0.20541	3.215449

	E-1 - MPTS (gas phase)		
Atom	X	Y	Z
C	1.100757	3.879497	0.079549
C	0.197411	2.842846	0.13541

	G-2 -MPTS (gas phase)		
Atom	X	Y	Z
C	0.117577	3.820188	-0.010497
C	0.6569	2.56421	-0.131496
C	-0.158707	1.412932	-0.128968
C	-1.557417	1.56394	-0.018748
C	-2.082026	2.864916	0.109238
C	-1.267612	3.970515	0.116582
C	0.42983	0.09118	-0.223076
C	-2.405549	0.384921	-0.065085
C	-1.814919	-0.889197	-0.216358
C	-0.368681	-1.01174	-0.251262
C	-2.65339	-2.015863	-0.337066
H	-2.198346	-2.982041	-0.504948
C	-4.0196	-1.901694	-0.277241
C	-4.604656	-0.644748	-0.092826
C	-3.810078	0.470506	0.00344
H	0.762263	4.690766	-0.015828
H	1.727238	2.446911	-0.234714
H	-3.14906	3.01051	0.199862
H	-1.700824	4.958706	0.214815
H	-4.641723	-2.782749	-0.379416
H	-5.682276	-0.547193	-0.038873
H	-4.285586	1.433206	0.126287
C	1.919966	-0.01345	-0.288691
C	2.701133	0.051928	0.872435
C	2.536533	-0.148335	-1.52881
C	4.085206	-0.038519	0.747686
C	3.916426	-0.231131	-1.636922
H	1.920454	-0.193953	-2.419507
C	4.694577	-0.178239	-0.490841
H	4.696336	0.001401	1.642898
H	4.378592	-0.340529	-2.610598
H	5.773945	-0.24619	-0.558173
S	0.375373	-2.638144	-0.377703
C	0.052698	-3.298097	1.287147
H	-1.011329	-3.303643	1.51868
H	0.422431	-4.323352	1.284968
H	0.5944	-2.730115	2.041252
C	2.075342	0.224995	2.230949
H	2.818917	0.101339	3.01892
H	1.632679	1.218674	2.339798
H	1.274602	-0.496747	2.400288
C	0.600897	1.495696	0.011197
C	1.992369	1.217081	-0.057374
C	2.883642	2.294829	-0.12953
C	2.457529	3.607297	-0.080747
C	-0.347948	0.411331	0.029569
C	2.474694	-0.17257	-0.002845
C	1.54197	-1.24293	0.0013
C	0.144288	-0.929473	-0.036601
C	2.034483	-2.566266	0.014585
H	1.334431	-3.391279	-0.009441
C	3.382977	-2.832568	0.061224
C	4.299652	-1.782712	0.084219
C	3.838841	-0.480948	0.048955
H	0.75444	4.902196	0.171638
H	-0.846296	3.068568	0.299249
H	3.944361	2.107954	-0.219604
H	3.17808	4.413742	-0.140368
H	3.727897	-3.859792	0.07869
H	5.363432	-1.981136	0.124261
H	4.568372	0.316539	0.065481
C	-1.773809	0.560452	0.142214
C	-2.512984	-0.382329	0.962339
C	-2.52477	1.446706	-0.666301
C	-3.900092	-0.518428	0.755596
C	-3.884006	1.306391	-0.808093
H	-2.002459	2.174134	-1.273423
C	-4.58237	0.289129	-0.12145
H	-4.441557	-1.240529	1.358106
H	-4.419071	1.958295	-1.488654
H	-5.650194	0.173676	-0.259303
S	-1.028568	-2.180594	-0.290475
C	-1.776426	-1.856894	-1.921873
H	-1.03478	-2.091748	-2.684656
H	-2.632966	-2.523809	-2.012717
H	-2.092824	-0.821042	-2.004465
C	-1.953626	-0.837291	2.286061
H	-2.497804	-1.704554	2.663515
H	-0.890561	-1.076542	2.23971
H	-2.066291	-0.028254	3.015161

	G-1 - MPTS PCM (ACN)		
Atom	X	Y	Z
C	-0.02352	3.818619	-0.343777
C	-0.595114	2.57433	-0.242225
C	0.196434	1.411091	-0.130413
C	1.60278	1.535965	-0.122388
C	2.16173	2.825086	-0.231897
C	1.370766	3.943542	-0.340188
C	-0.421699	0.103546	-0.029348
C	2.4233	0.342396	0.002893
C	1.79963	-0.919981	0.12649
C	0.351107	-1.009992	0.094379
C	2.608473	-2.065677	0.274217
H	2.129536	-3.027774	0.390823
C	3.978743	-1.978423	0.286663
C	4.597627	-0.730414	0.152285
C	3.831577	0.401793	0.016976
H	-0.64925	4.698878	-0.427302
H	-1.672292	2.478163	-0.248487
H	3.234832	2.952556	-0.229686
H	1.829212	4.921686	-0.421699
H	4.577399	-2.873794	0.402735
H	5.678174	-0.653973	0.159078
H	4.333494	1.354094	-0.07911
C	-1.913875	0.022049	-0.091586
C	-2.532835	-0.081184	-1.334404
C	-2.691733	0.084067	1.071977
C	-3.915513	-0.142303	-1.441269
H	-1.920744	-0.113958	-2.228757
C	-4.078131	0.024705	0.948292
C	-4.691507	-0.089628	-0.292422
H	-4.381138	-0.227198	-2.415867
H	-4.687701	0.069135	1.844251
H	-5.772201	-0.133564	-0.359439
S	-0.434961	-2.613016	0.263574
C	-0.243488	-3.268326	-1.422803
H	0.806914	-3.330235	-1.702568
H	-0.671844	-4.270063	-1.416747
H	-0.785934	-2.649235	-2.135194
C	-2.053986	0.192325	2.430758
H	-1.485858	-0.710132	2.671532
H	-1.358025	1.032641	2.481711
H	-2.80978	0.329514	3.204283

	G-2 - MPTS PCM (ACN)		
Atom	X	Y	Z
C	0.121142	3.824075	0.012412
C	0.660441	2.567509	-0.110939
C	-0.156094	1.416318	-0.119217
C	-1.555684	1.568329	-0.015549
C	-2.081528	2.869489	0.114233
C	-1.265566	3.974932	0.130905
C	0.431966	0.094068	-0.215332
C	-2.404911	0.389655	-0.068853
C	-1.814407	-0.886035	-0.21413
C	-0.367583	-1.008356	-0.243365
C	-2.654709	-2.012048	-0.336393
H	-2.205591	-2.983113	-0.490261
C	-4.021966	-1.895275	-0.28797
C	-4.606597	-0.636054	-0.112413
C	-3.810507	0.478819	-0.011659
H	0.766376	4.694155	0.017648
H	1.73187	2.452023	-0.203376
H	-3.148645	3.016928	0.200106
H	-1.69859	4.962847	0.231269
H	-4.644503	-2.776	-0.3893
H	-5.684312	-0.536503	-0.067177
H	-4.286418	1.441903	0.106124
C	1.922165	-0.010572	-0.288621
C	2.71054	0.039236	0.869071
C	2.530342	-0.130572	-1.535424
C	4.09457	-0.054182	0.734197
C	3.910315	-0.215982	-1.653699
H	1.909549	-0.161437	-2.423733
C	4.69612	-0.180418	-0.510705
H	4.711496	-0.026366	1.625761
H	4.366284	-0.312231	-2.631782
H	5.774969	-0.249663	-0.585499
S	0.375905	-2.635956	-0.366293
C	0.018726	-3.328397	1.279069
H	-1.050997	-3.370127	1.474187
H	0.420194	-4.341227	1.273463
H	0.517896	-2.752757	2.055659
C	2.094977	0.198837	2.233737
H	2.843366	0.063362	3.014679
H	1.659779	1.194339	2.355863
H	1.292268	-0.521097	2.400905

	G-1 - MPTS PCM (CCL_4)		
Atom	X	Y	Z
C	0.01032	3.822346	-0.305533

	E-1 - MPTS PCM (ACN)		
Atom	X	Y	Z
C	0.261528	3.809634	0.018865
C	-0.39794	2.606722	-0.049341
C	0.304505	1.361538	-0.012305
C	1.742094	1.411056	0.006189
C	2.368756	2.647323	0.104977
C	1.653837	3.841471	0.125718
C	-0.371456	0.131748	-0.073062
C	2.508865	0.160743	-0.150497
C	1.820686	-1.08485	-0.103049
C	0.402139	-1.099144	0.055957
C	2.573866	-2.276732	-0.207571
H	2.051847	-3.225103	-0.208465
C	3.947394	-2.251646	-0.355728
C	4.613087	-1.033775	-0.42686
C	3.888907	0.151942	-0.330416
H	-0.303359	4.734349	-0.003631
H	-1.475859	2.594002	-0.137053
H	3.448055	2.700915	0.154903
H	2.179122	4.785206	0.205176
H	4.496755	-3.181837	-0.439039
H	5.68684	-0.999817	-0.563301
H	4.431795	1.085528	-0.390519
C	-1.832091	0.072907	-0.330161
C	-2.796463	0.415793	0.634813
C	-2.252507	-0.34109	-1.597576
C	-4.144975	0.342752	0.284521
C	-3.596045	-0.401116	-1.930341
H	-1.50253	-0.613056	-2.331863
C	-4.550012	-0.056206	-0.981149
H	-4.892509	0.59819	1.027672
H	-3.896106	-0.715194	-2.923061
H	-5.605393	-0.101395	-1.222698
S	-0.211048	-2.386116	1.04507
C	-1.760827	-3.006901	0.329676
H	-1.625206	-3.189162	-0.734528
H	-1.952313	-3.944336	0.851845
H	-2.585433	-2.318528	0.495223
C	-2.409869	0.806094	2.035605
H	-3.293207	0.888432	2.669451
H	-1.73917	0.065018	2.477109
H	-1.887456	1.765059	2.062063

C	-0.570442	2.58187	-0.216325
C	0.212484	1.411866	-0.118376
C	1.61965	1.525748	-0.11131
C	2.18728	2.811548	-0.20836
C	1.405041	3.936682	-0.303274
C	-0.415104	0.108555	-0.025361
C	2.430976	0.325164	0.001069
C	1.7979	-0.932667	0.118654
C	0.34903	-1.011401	0.092416
C	2.597286	-2.086039	0.253754
H	2.107359	-3.042807	0.368923
C	3.967818	-2.01039	0.258786
C	4.596319	-0.767092	0.130034
C	3.839313	0.372007	0.007687
H	-0.609115	4.708072	-0.378073
H	-1.648178	2.49266	-0.221412
H	3.261449	2.930035	-0.205806
H	1.870741	4.912285	-0.374848
H	4.559561	-2.911516	0.366152
H	5.677586	-0.699838	0.131578
H	4.347884	1.321188	-0.084137
C	-1.907732	0.037877	-0.089838
C	-2.52617	-0.008807	-1.336039
C	-2.686941	0.058117	1.073761
C	-3.90861	-0.054591	-1.447314
H	-1.9122	-0.005042	-2.229801
C	-4.072882	0.016133	0.945584
C	-4.685371	-0.042608	-0.298712
H	-4.373588	-0.094843	-2.425096
H	-4.683264	0.028444	1.84203
H	-5.766265	-0.075151	-0.368637
S	-0.44989	-2.608114	0.259066
C	-0.350507	-3.211653	-1.453557
H	0.683252	-3.258999	-1.793297
H	-0.774024	-4.215695	-1.454892
H	-0.935093	-2.57599	-2.116613
C	-2.050198	0.100314	2.436112
H	-1.526084	-0.834861	2.650506
H	-1.316538	0.90559	2.513401
H	-2.802009	0.248424	3.211817

	E-1 - MPTS PCM (CCL_4)

	G-2 MPTS PCM (CCL_4)		
Atom	X	Y	Z
C	0.119606	3.821551	-0.001624
C	0.658674	2.565344	-0.124939
C	-0.157306	1.414122	-0.125671
C	-1.556199	1.565616	-0.016746
C	-2.081067	2.866555	0.113767
C	-1.265983	3.972073	0.124521
C	0.430938	0.092048	-0.219825
C	-2.404964	0.386986	-0.06695
C	-1.814596	-0.887829	-0.215452
C	-0.368182	-1.010606	-0.24643
C	-2.653949	-2.013888	-0.338825
H	-2.201204	-2.982009	-0.501382
C	-4.020648	-1.898237	-0.28596
C	-4.605412	-0.640253	-0.10534
C	-3.80996	0.474474	-0.005259
H	0.764602	4.691911	-0.00354
H	1.72928	2.448618	-0.22564
H	-3.148084	3.012759	0.204035
H	-1.698885	4.960156	0.22496
H	-4.64314	-2.778776	-0.390101
H	-5.683179	-0.541503	-0.05726
H	-4.285289	1.43763	0.1147
C	1.921035	-0.0132	-0.28955
C	2.706775	0.049261	0.868894
C	2.532345	-0.146942	-1.532755
C	4.090588	-0.043201	0.738512
C	3.912066	-0.231635	-1.646545
H	1.912965	-0.189857	-2.421388
C	4.694962	-0.181783	-0.503037
H	4.705279	-0.005823	1.631379
H	4.370286	-0.339926	-2.622269
H	5.774021	-0.251055	-0.574706
S	0.37601	-2.637802	-0.365432
C	0.032129	-3.307205	1.291665
H	-1.035479	-3.330413	1.50338
H	0.418447	-4.32617	1.292699
H	0.55084	-2.73215	2.056206
C	2.086957	0.220512	2.230332
H	2.833408	0.093543	3.014893
H	1.647616	1.215289	2.342917
H	1.285487	-0.499974	2.401328

Atom	X	Y	Z
C	1.076401	3.883651	0.066282
C	0.178549	2.84142	0.117895
C	0.590436	1.495329	0.000797
C	1.985035	1.226022	-0.056416
C	2.870681	2.308462	-0.123071
C	2.436425	3.6195	-0.081269
C	-0.351726	0.405764	0.018175
C	2.475843	-0.161357	0.003463
C	1.548239	-1.23746	-0.003565
C	0.149726	-0.935151	-0.051453
C	2.050224	-2.558606	0.005749
H	1.35556	-3.387685	-0.032433
C	3.400304	-2.816442	0.064332
C	4.31038	-1.760765	0.101592
C	3.841189	-0.4608	0.066284
H	0.722698	4.904438	0.151665
H	-0.867754	3.062445	0.270621
H	3.93344	2.128173	-0.202607
H	3.152865	4.429938	-0.136584
H	3.75161	-3.841608	0.078751
H	5.375092	-1.952372	0.150611
H	4.566096	0.340813	0.090488
C	-1.777649	0.549668	0.136813
C	-2.512387	-0.381428	0.971757
C	-2.532238	1.430152	-0.676328
C	-3.901027	-0.512905	0.780917
C	-3.893049	1.295277	-0.803731
H	-2.012553	2.146674	-1.298517
C	-4.588732	0.290027	-0.097185
H	-4.439731	-1.22711	1.395008
H	-4.431364	1.940433	-1.488085
H	-5.658576	0.178321	-0.222456
S	-1.01152	-2.198299	-0.298575
C	-1.770706	-1.880959	-1.925557
H	-1.036499	-2.122176	-2.693392
H	-2.628862	-2.547009	-2.004834
H	-2.085711	-0.845044	-2.011053
C	-1.938333	-0.841277	2.286834
H	-2.480575	-1.707503	2.668932
H	-0.876631	-1.083238	2.228158
H	-2.040301	-0.033433	3.019033

	G-1 - MPTS PCM (MeOH)		
Atom	X	Y	Z
C	-0.023374	3.818625	-0.343571
C	-0.595003	2.57435	-0.242087
C	0.19651	1.411083	-0.130347
C	1.602857	1.535912	-0.122329
C	2.161838	2.82502	-0.231768
C	1.370912	3.943505	-0.339985
C	-0.421663	0.103554	-0.029325
C	2.42334	0.342314	0.002884
C	1.799631	-0.920042	0.126459
C	0.351108	-1.01001	0.09437
C	2.608433	-2.065768	0.274142
H	2.129432	-3.02783	0.390786
C	3.978702	-1.978566	0.286547
C	4.597627	-0.73058	0.152171
C	3.831615	0.401652	0.016921
H	-0.64908	4.698906	-0.427039
H	-1.672184	2.478203	-0.248349
H	3.234945	2.952451	-0.229549
H	1.829387	4.921641	-0.421439
H	4.577329	-2.873959	0.402602
H	5.678177	-0.65418	0.158936
H	4.333556	1.353942	-0.079155
C	-1.913843	0.022106	-0.091566
C	-2.532821	-0.080807	-1.334397
C	-2.691692	0.083889	1.07201
C	-3.915498	-0.141812	-1.441272
H	-1.92073	-0.113378	-2.228759
C	-4.078088	0.024663	0.948315
C	-4.691479	-0.08934	-0.292415
H	-4.381135	-0.226456	-2.415887
H	-4.687647	0.068914	1.844291
H	-5.772176	-0.133191	-0.359436
S	-0.435015	-2.613011	0.263498
C	-0.244065	-3.267901	-1.423092
H	0.806245	-3.329555	-1.703271
H	-0.672246	-4.269716	-1.417117
H	-0.786897	-2.648757	-2.135145
C	-2.053924	0.191712	2.430811
H	-1.486214	-0.71104	2.671465
H	-1.357584	1.031704	2.48189
H	-2.809673	0.329123	3.204343

	G-2 - MPTS PCM (MeOH)		
Atom	X	Y	Z
C	0.121171	3.824044	0.012327
C	0.660454	2.567476	-0.111041
C	-0.156087	1.416293	-0.119256
C	-1.555669	1.568314	-0.015539
C	-2.081491	2.869476	0.114272
C	-1.265525	3.974911	0.130902
C	0.43196	0.094038	-0.215348
C	-2.404905	0.389653	-0.068842
C	-1.814415	-0.886037	-0.214133
C	-0.367596	-1.00838	-0.243351
C	-2.65472	-2.012037	-0.336444
H	-2.205577	-2.98308	-0.490388
C	-4.021972	-1.895255	-0.288032
C	-4.606594	-0.636037	-0.112444
C	-3.810496	0.478822	-0.01166
H	0.766412	4.69412	0.017501
H	1.731873	2.451969	-0.203564
H	-3.148606	3.016911	0.200193
H	-1.698537	4.962832	0.231282
H	-4.644519	-2.775967	-0.389427
H	-5.68431	-0.536477	-0.067231
H	-4.286389	1.441916	0.106119
C	1.922157	-0.010622	-0.288638
C	2.710546	0.039314	0.869034
C	2.53032	-0.130772	-1.535427
C	4.09457	-0.054087	0.734157
C	3.910287	-0.216173	-1.653705
H	1.90951	-0.161766	-2.423717
C	4.696103	-0.180449	-0.510734
H	4.711505	-0.026175	1.625713
H	4.366242	-0.312556	-2.63178
H	5.774953	-0.249695	-0.585535
S	0.375892	-2.635983	-0.366179
C	0.018709	-3.328191	1.279274
H	-1.051013	-3.369771	1.474463
H	0.420064	-4.341068	1.273778
H	0.517985	-2.75253	2.055784
C	2.094983	0.199013	2.23369
H	2.843389	0.06367	3.014642
H	1.659688	1.194483	2.355729
H	1.292336	-0.520975	2.40093

	E-1 - MPTS PCM (MeOH)		
Atom	X	Y	Z
C	0.261651	3.809617	0.01859
C	-0.397864	2.606728	-0.049545
C	0.30453	1.361545	-0.012397
C	1.742099	1.411009	0.006116
C	2.36882	2.647255	0.104856
C	1.65395	3.841413	0.125489
C	-0.371469	0.131747	-0.073134
C	2.508822	0.160694	-0.150489
C	1.820636	-1.084889	-0.102952
C	0.402072	-1.099135	0.055928
C	2.573847	-2.276764	-0.207316
H	2.051855	-3.225149	-0.208107
C	3.947369	-2.251669	-0.355439
C	4.613058	-1.033802	-0.426718
C	3.888864	0.151899	-0.330408
H	-0.303197	4.734352	-0.004029
H	-1.475775	2.594035	-0.13735
H	3.448123	2.700777	0.154832
H	2.179262	4.785136	0.204896
H	4.496746	-3.181864	-0.438626
H	5.68681	-0.999853	-0.563167
H	4.431711	1.085505	-0.390626
C	-1.832111	0.072946	-0.330163
C	-2.796414	0.415754	0.634908
C	-2.252628	-0.340891	-1.597593
C	-4.144951	0.342782	0.2847
C	-3.596185	-0.400851	-1.930271
H	-1.50269	-0.612717	-2.331971
C	-4.550084	-0.056036	-0.980979
H	-4.892427	0.598177	1.027925
H	-3.896321	-0.714767	-2.92302
H	-5.605482	-0.101145	-1.222474
S	-0.211272	-2.385909	1.045204
C	-1.760542	-3.007304	0.329228
H	-1.624359	-3.189963	-0.734839
H	-1.952122	-3.944569	0.851677
H	-2.58537	-2.319022	0.494058
C	-2.409684	0.805912	2.0357
H	-3.292963	0.888232	2.669634
H	-1.738965	0.064777	2.477077
H	-1.887209	1.764841	2.062199

	G-1 - MPTS PCM (Water)		
Atom	X	Y	Z
C	-0.02404	3.818682	-0.344712
C	-0.595522	2.574336	-0.242914
C	0.196144	1.411196	-0.130729
C	1.60249	1.536225	-0.122621
C	2.161361	2.825391	-0.23234
C	1.370259	3.943751	-0.340984
C	-0.421869	0.103599	-0.029462
C	2.423137	0.342731	0.002917
C	1.799578	-0.919718	0.126625
C	0.351046	-1.009855	0.094447
C	2.608555	-2.065324	0.274489
H	2.129912	-3.027592	0.390864
C	3.978842	-1.977912	0.287048
C	4.597603	-0.72982	0.152624
C	3.831433	0.40232	0.017108
H	-0.649833	4.698862	-0.428539
H	-1.672697	2.47813	-0.249231
H	3.234437	2.953023	-0.230066
H	1.828606	4.921918	-0.422677
H	4.577587	-2.87322	0.403089
H	5.678133	-0.653261	0.159445
H	4.333297	1.354636	-0.079085
C	-1.914021	0.021937	-0.091762
C	-2.532821	-0.082882	-1.334556
C	-2.691986	0.085102	1.071709
C	-3.915504	-0.144647	-1.441438
H	-1.920691	-0.116654	-2.228837
C	-4.078396	0.024968	0.948006
C	-4.691629	-0.091052	-0.292664
H	-4.381017	-0.230796	-2.415976
H	-4.688073	0.070316	1.843842
H	-5.772299	-0.135446	-0.35972
S	-0.434829	-2.612925	0.264183
C	-0.240816	-3.270763	-1.420982
H	0.810024	-3.334179	-1.698659
H	-0.670195	-4.272046	-1.414274
H	-0.781233	-2.652004	-2.135184
C	-2.054437	0.195414	2.430442
H	-1.485743	-0.706361	2.672503
H	-1.359011	1.036227	2.480383
H	-2.810383	0.333184	3.203695

	G-2 - MPTS PCM (Water)		
Atom	X	Y	Z
C	0.121263	3.824166	0.012262
C	0.660559	2.567557	-0.110957
C	-0.156014	1.416368	-0.119298
C	-1.555635	1.568437	-0.015778
C	-2.081502	2.86962	0.113919
C	-1.26548	3.975058	0.130614
C	0.432018	0.094083	-0.215235
C	-2.404906	0.38978	-0.069066
C	-1.814424	-0.886005	-0.213929
C	-0.367566	-1.00834	-0.24304
C	-2.654809	-2.012026	-0.335918
H	-2.20588	-2.983279	-0.489079
C	-4.022093	-1.895146	-0.287766
C	-4.60668	-0.635801	-0.112779
C	-3.810532	0.479088	-0.012202
H	0.76651	4.694228	0.017619
H	1.732017	2.452111	-0.203076
H	-3.148602	3.017127	0.199826
H	-1.698488	4.962967	0.231001
H	-4.644652	-2.775892	-0.388701
H	-5.684385	-0.53616	-0.067729
H	-4.28647	1.442207	0.105208
C	1.922223	-0.010538	-0.288619
C	2.710696	0.039179	0.869051
C	2.530306	-0.130365	-1.53553
C	4.094738	-0.054461	0.734044
C	3.910301	-0.215956	-1.653941
H	1.909484	-0.16094	-2.42384
C	4.696201	-0.180698	-0.510945
H	4.711741	-0.026674	1.625544
H	4.366205	-0.31198	-2.632079
H	5.77504	-0.249974	-0.585819
S	0.375797	-2.636053	-0.365726
C	0.018171	-3.329168	1.279279
H	-1.051616	-3.371762	1.473698
H	0.420362	-4.341695	1.27366
H	0.516447	-2.753258	2.056225
C	2.095312	0.198945	2.233769
H	2.84384	0.063755	3.014603
H	1.66009	1.194455	2.355712
H	1.292718	-0.52105	2.401245

	E-1 - MPTS PCM (Water)		
Atom	X	Y	Z
C	0.261141	3.809838	0.017867
C	-0.398265	2.606931	-0.050455
C	0.304252	1.361483	-0.012715
C	1.74207	1.411306	0.006463
C	2.368526	2.647522	0.105163
C	1.653462	3.841797	0.12537
C	-0.371442	0.131983	-0.073006
C	2.509008	0.160918	-0.149932
C	1.820723	-1.084728	-0.102906
C	0.402242	-1.099155	0.056797
C	2.573933	-2.276567	-0.208384
H	2.051922	-3.22493	-0.210241
C	3.947571	-2.251381	-0.356365
C	4.613307	-1.033534	-0.426286
C	3.889025	0.152253	-0.329354
H	-0.303771	4.734529	-0.004994
H	-1.47616	2.594206	-0.138414
H	3.44781	2.701337	0.155305
H	2.178704	4.785549	0.20487
H	4.496868	-3.181541	-0.440427
H	5.68711	-0.999434	-0.562272
H	4.432028	1.085827	-0.388648
C	-1.832101	0.072839	-0.330093
C	-2.796475	0.416192	0.63466
C	-2.252399	-0.341961	-1.597307
C	-4.144988	0.342964	0.28433
C	-3.595931	-0.402131	-1.930139
H	-1.50249	-0.614754	-2.331369
C	-4.549945	-0.056633	-0.981179
H	-4.892582	0.598632	1.027332
H	-3.895917	-0.716993	-2.922629
H	-5.605319	-0.102054	-1.222713
S	-0.210036	-2.387593	1.044323
C	-1.761931	-3.006251	0.33174
H	-1.628727	-3.187931	-0.732844
H	-1.952743	-3.943975	0.853597
H	-2.585663	-2.317514	0.499831
C	-2.410059	0.807464	2.035254
H	-3.29312	0.885987	2.669942
H	-1.735968	0.06896	2.475832
H	-1.89175	1.768694	2.061532
